# Family networks and infant health promotion: a mixed-methods evaluation from a cluster randomised controlled trial in rural Malawi

**DOI:** 10.1136/bmjopen-2017-019380

**Published:** 2018-06-07

**Authors:** Molly Scott, Bansi Malde, Carina King, Tambosi Phiri, Hilda Chapota, Esther Kainja, Florida Banda, Marcos Vera-Hernandez

**Affiliations:** 1 Oxford Policy Management, Oxford, UK; 2 Centre for Evaluation of Development Policies, Institute for Fiscal Studies, London, UK; 3 School of Economics, University of Kent, London, UK; 4 Institute for Global Health, University College London, London, UK; 5 MaiMwana Project, Mchinji, Malawi; 6 Department of Economics, University College London, London, UK

**Keywords:** nutrition, extended family, health promotion, sub-saharan africa, child health

## Abstract

**Objective:**

Parents may rely on information provided by extended family members when making decisions concerning the health of their children. We evaluate whether extended family members affected the success of an information intervention promoting infant health.

**Methods:**

This is a secondary, sequential mixed-methods study based on a cluster randomised controlled trial of a peer-led home-education intervention conducted in Mchinji District, Malawi. We used linear multivariate regression to test whether the intervention impact on child height-for-age z-scores (HAZ) was influenced by extended family members. 12 of 24 clusters were assigned to the intervention, in which all pregnant women and new mothers were eligible to receive 5 home visits from a trained peer counsellor to discuss infant care and nutrition. We conducted focus group discussions with mothers, grandmothers and peer counsellors, and key-informant interviews with husbands, chiefs and community health workers to better understand the roles of extended family members in infant feeding.

**Results:**

Exposure to the intervention increased child HAZ scores by 0.296 SD (95% CI 0.116 to 0.484). However, this effect is smaller in the presence of paternal grandmothers. Compared with an effect size of 0.441 to 0.467 SD (95% CI −0.344 to 1.050) if neither grandmother is alive, the effect size was 0.235 (95% CI −0.493 to 0.039) to 0.253 (95% CI −0.529 to 0.029) SD lower if the paternal grandmother was alive. There was no evidence of an effect of parents’ siblings. Maternal grandmothers did not affect intervention impact, but were associated with a lower HAZ score in the control group. Qualitative analysis suggested that grandmothers, who act as secondary caregivers and provide resources for infants, were slower to dismiss traditionally held practices and adopt intervention messages.

**Conclusion:**

The results indicate that the intervention impacts are diminished by paternal grandmothers. Intervention success could be increased by integrating senior women.

**Strengths and limitations of this study**

- Uses mixed-methods to understand how extended family members affected the success of an infant feeding promotion programme in Malawi.
- Quantitative analysis, using linear multivariate regression, allows estimation of the size of effect that different extended family members—including paternal and maternal grandmothers—have on the impact of the intervention.
- Focus group discussions and key informant interviews help shed light on the mechanisms through which extended family members might have affected the intervention’s success.
- The interval between qualitative and quantitative data collection is a limitation, with potential changes to infant feeding practices over time and recall bias.
- Interventions on infant feeding would benefit from understanding and addressing extended family dynamics to improve reach and impact.

## Introduction

Child health outcomes are influenced by individuals besides the mother and father, with a rich literature devoted to the contribution of extended family members.^1–5^ In low-income settings, where risk of poor health is high and social welfare nets are minimal, the support of the extended family may be crucial in child-rearing.^6^ Relatives can assist by acting as secondary caregivers,^7^ supplying labour, donating money and providing other in-kind resources to the household.^1 8–11^ Older women in the family can also affect child outcomes by dispensing infant health and nutrition advice to new mothers.^4 5 12–14^ Finally, relatives may exert pressure on mothers, impacting their infant nutrition and health practices.^15^

Extended family members may influence the effectiveness of policies and interventions designed to improve child health in low-income settings. On the one hand, they may provide resources and support that complement the intervention making it more effective. On the other hand, they could be resistant to change and reinforce traditional practices, thereby undermining interventions. The latter is particularly relevant in the case of health education and outreach programmes, which are widespread across both developed and developing country settings.

Educational programmes have the potential to improve child health outcomes by changing widespread misconceptions and traditional behaviours around child feeding and care in low-income settings. In Malawi, for instance, although most infants are breast fed for at least a year, only 40.5% of infants are still exclusively breast fed at 5 months,^16^ while diets of children aged over 6 months usually lack sufficient diversity.^17 18^ Beliefs surrounding feeding practices for older infants include the view that the broth of a soup is more nourishing than the vegetables or meat inside and that eggs are harmful for children aged 9 months.

Education campaigns promoting better infant feeding and care to caregivers of infants have shown mixed success in developing country settings. In some cases, they have led to sustained improvements in feeding practices^14 19^ and child physical growth.^20–22^ In others, they have had a negligible effect on child physical growth.^23–25^ Given these mixed findings, there is a need for greater understanding of the factors shaping responses to such interventions across contexts.

Little attention has been paid to the role of extended family members in influencing the success of caregiver focused education interventions in the existing literature, despite their important role in shaping child health. Much of this existing literature is qualitative, with small samples.^26 27^ The small number of quantitative evaluations of education interventions to improve infant feeding that seek to involve extended family members find mixed evidence of effectiveness. Counselling sessions for new adolescent mothers and coresident grandmothers reduced the unnecessary intake of water and herbal teas within the first 6 months of the child’s life in Brazil^28^ but failed to maintain breast feeding of infants at age 2 years,^29^ while a behavioural change communication programme delivered through older female leaders in Burkina Faso improved infant feeding knowledge but failed to improve child health outcomes.^14^

A cluster randomised controlled trial (RCT) conducted in Malawi used a peer-led home-education strategy to improve rates of exclusive breast feeding, and reduce infant mortality.^30 31^ The trial achieved a 36% reduction in infant mortality and an improvement in children’s height-for-age z-score (HAZ) (increased by 0.271 SD; p=0.022).^17 31^ This paper uses mixed-methods to investigate whether members of the extended family influenced the success of this peer home-visiting intervention, and the possible mechanisms through which their influence might work.

## Methods

This is a secondary, sequential mixed-methods study based on a cluster RCT of a peer-led home-education intervention conducted in Mchinji District, Malawi. We investigated family member roles in the success of the intervention, which provided information on healthy infant feeding practices, with quantitative data collected between November 2008 and January 2010, and qualitative data collected in December 2015. Full details of the original trial and methods have previously been published.^30 31^ The quantitative data were collected and analysed first, and used to design the qualitative aspect of the study. The overall interpretation of our findings was integrated following analysis of the qualitative data.

### Setting

Mchinji is a rural district in central Malawi with a population of about 455 000.^32^ Maternal and infant healthcare is delivered at one district hospital, four rural hospitals, nine health centres, private clinics and in the community through government employed community health workers (CHW—known locally as Health Surveillance Assistants). Much of the healthcare received by pregnant women and infants is in the community setting by CHWs, or at home by kin and other social contacts. However, Malawi has medical pluralism, with traditional practices, beliefs and behaviours such as witchcraft and herbal medicine being commonly used alongside Western medicine. The 2010 Malawi Demographic and Health Survey reported 24% of births in the region occurred in the woman’s own home and 2% in someone else’s home, and many births are not attended by medically trained healthcare personnel but by traditional birth attendants (14.4%), friends and relatives (8.7%) or no one (2.6%).^16^ Without access to a skilled birth attendant, women are more vulnerable to infection and complications during birth; the infant mortality rate in 2010 was of 66 per 1000 live births.^16^

Traditionally, the main ethnic group in the study area, the Chewa, are a matrilineal and matrilocal group. Matriliny is a system in which land is passed through the female line. Under traditional matrilocal norms, husbands move to their wives’ homes after marriage unless they make a special payment. However, following the influence of patrilineal and patrilocal ethnic groups and British colonialists, there is evidence that matrilocality has waned over time, but not completely.^33 34^ As a result, women often remain in close proximity to their own relatives after marriage.

### Intervention description

For the RCT, Mchinji was divided into 48 approximately equal population clusters based on the 1998 Malawi Population and Housing census (the most recent census at the time of trial planning). Within each cluster of around 8000 people, the 3000 individuals living in villages closest to the geographical centre were enumerated as the eligible study population. Twelve clusters were assigned to the infant feeding intervention only and 12 served as controls. Full details of the trial set-up and methods are described by Lewycka *et al*.^30^ All women living in clusters assigned to the infant feeding intervention who became pregnant during the trial period were eligible to receive five home visits from a trained local woman volunteer (‘peer counsellor’) to discuss maternal and infant healthcare issues; around 60% of eligible women reported having been visited. The visits were timed to coincide with key stages of infant development (the third trimester, and at 1 week, 1 month, 3 and 5 months after birth). Each visit focused on a specific set of topics for discussion, with special attention paid to nutrition practices including exclusive breast feeding, and complementary feeding. Peer counsellors were literate local women aged 23–50 years with breastfeeding experience, who each covered a population of about 1000 people.

The intervention began in December 2004, with an initial establishment period until June 2005. The trial was ongoing at the time of the quantitative data collection. Following the end of the trial period, peer counsellors continued to receive mentorship and supervision support from government CHWs and the local implementing non-governmental organisation (NGO). In 2015, at the time of qualitative data collection, approximately one-third of the volunteer counsellors were still active in delivering the intervention.

### Quantitative analysis

#### Data and sample selection

A baseline census was conducted in all clusters in 2004, prior to the start of the intervention. All women aged between 10 and 49 years were enumerated and a random sample of 104 women aged between 17 and 43 years per cluster was then drawn to be interviewed for two follow-up quantitative surveys as part of this secondary study. Sampled women (‘main respondent’ hereon) were visited to complete the first follow-up in November 2008 to March 2009, and a second follow-up in October 2009 to January 2010.

Each follow-up survey contained questions about the size of the extended family of the main respondent and her husband (those alive and those in the village), the health of all household members, food and liquid intake of children aged under 6 years, knowledge about child nutrition, intervention participation (in treatment clusters) and socioeconomic variables such as adult work. The height of the main respondent and the height and weight of children under 6 years were also collected by trained enumerators.

The main outcome for our analysis is the child HAZ score, which is a long-term indicator of health that reflects nutrition and morbidity since birth, and should be sensitive to any effects of intervention exposure in early life. It is calculated by comparing the height of the child with the median height in the WHO reference population of children of the same gender and age in months.^35^

The sample was balanced between treatment and control clusters along a range of variables collected at baseline^17^ (table 1). The baseline characteristics of the two groups remained similar even after accounting for attrition between the baseline and first endline survey, indicating that randomisation was not jeopardised (table 1).

**Table 1:** Distribution of household and women characteristics in controls and differences with treatment group

	Analysis sample		
	Control group (mean or proportion)	Difference: treatment−control	p values
**Household characteristics**			
Number of members†	5.621	0.114	0.875
Number of sleeping rooms†	2.036	0.232	0.034**
Household has electricity?‡	0.2%	0.0%	0.827
Household has radio?‡	65.1%	0.4%	0.897
Household has bicycle?‡	49.9%	1.9%	0.699
Household has motorbike?‡	0.8%	−0.1%	0.879
Household has paraffin lamp?‡	93.9%	1.7%	0.815
Household has oxcart?‡	5.1%	−1.9%	0.198
Agricultural household‡	100%	−0.2%	0.422
Main flooring material: dirt, sand or dung‡	92.4%	−1.7%	0.565
Main roofing material: natural material‡	87.6%	−1.7%	0.697
Piped water‡	1.5%	2.2%	0.494
Traditional pit toilet‡	78.3%	4.4%	0.356
Wealth index†	−0.087	0.034	0.897
**Woman characteristics**			
Married‡	71.8%	−4.9%	0.046**
Completed primary education‡	70.9%	2.8%	0.529
Completed secondary education‡	7.6%	−2.2%	0.268
Age†	24.592	−0.993	0.026**
Chewa‡	95.4%	−3.9%	0.452
Christian‡	98.3%	0.5%	0.609
Farmer‡	70.9%	−4.5%	0.316
Student‡	16.4%	2.3%	0.380
Small business owner‡	4.0%	2.1%	0.356
N	411	475	

Household and mother level characteristics in 2004 corresponding to married main respondent mothers present in the second follow-up survey with children born after the intervention began in July 2005.

*p<0.1, **p<0.05, ***p<0.01. P values are calculated using the wild cluster bootstrap t procedure described by Cameron *et al*.^37^

†Continuous variable, for which the mean is reported.

‡ Binary variable, for which proportions are reported.

For this analysis, we use a sample of children who were born since July 2005; and whose mothers are married main respondents in the follow-up surveys (80% of the sample). This sample selection ensures that we measure effects on children whose mothers were eligible to receive visits from a peer counsellor; and allows us to compare effects of the mothers’ relatives with those of her husband. Children in the estimation sample were aged between 0 and 53 months at the time of the endline surveys. Online supplementary appendix 1 provides a timeline of the original trial and the quantitative data collection, and of our sample inclusion criteria.

10.1136/bmjopen-2017-019380.supp1 Supplementary appendix 1

Table 1 presents the means of basic demographic and socioeconomic characteristics for women in the analysis sample living in control clusters at baseline, the differences in the means between the control and treatment groups and the p value of this difference. The last two columns allow us to assess whether the randomisation holds in our selected sample. Women assigned to the control group were 24.6 years on average; 71.8% were married and while 70.1% had completed at least primary education, only 7.6% had completed secondary education. In line with the general profile of communities in Mchinji, 95.4% of sampled women were Chewa ethnicity and 98.3% were Christian. The average household size was 5.6 members and all households were engaged in agricultural activity.

Table 2 displays statistics on the size of extended family networks of the children in our analysis sample. Most children have their grandmothers alive (87.3% have maternal grandmothers alive and 80.7% have paternal grandmothers) and their parents have a relatively large number of siblings, with an average of more than two brothers and two sisters each.

**Table 2:** Distribution of family networks indicators in controls and differences with treatment group for sampled children.

	Control group	Difference: treatment− control	p values	N
Maternal grandmother*	87.3%	1.2%	0.739	2260
Paternal grandmother*	80.7%	6.3%	0.132	2252
Mother’s sisters†	2.835	0.047	0.835	2266
Mother’s brothers†	2.556	0.207	0.180	2263
Father’s sisters†	2.336	0.246	0.290	2266
Father’s brothers†	2.453	0.213	0.288	2267

Sample includes all children born since July 2005, who were aged 0–53 months at the time of interview, and whose mothers were married main respondents to the follow-up surveys in 2008–2009 and 2009–2010. A pooled dataset from both follow-up surveys is used to construct means.

*Binary variable for which percentages are reported.

† Discrete, non-binary variables for which mean values are reported.

#### Model specification and estimation

The quantitative analysis aims to determine how different family members influence the effectiveness of the infant feeding intervention. Before estimating the main model, we study the relationship between baseline characteristics of mothers and their households and measures of the extended family using linear regression. Table 3 reports these results. It indicates that children whose grandmothers are alive have on average younger mothers, who are more likely to have completed at least primary education, less likely to be working as farmers in 2004 and are from more socioeconomically advantaged households, as measured by a composite wealth index constructed using principal components analysis as recommended by Filmer and Pritchett.^36^

**Table 3:** Relationship between baseline characteristics and family network size, with p values

Panel A: household characteristics			
	Number of members	Number of rooms	Wealth index
Maternal grandmother alive (0/1)	0.027	−0.04	0.256**
SE	(0.307)	(0.103)	(0.114)
Cluster wild bootstrap t p value	(0.929)	(0.779)	(0.032)
Paternal grandmother alive (0/1)	−0.209	−0.02	−0.129
SE	(0.276)	(0.094)	(0.186)
Cluster wild bootstrap t p value	(0.478)	(0.919)	(0.549)
Parents siblings alive	0.025	0.01	0.016
SE	(0.037)	(0.011)	(0.011)
Cluster wild bootstrap t p value	(0.627)	(0.400)	(0.144)
N	881	879	881

Panel B: mother characteristics								
	Primary education	Secondary education	Age	Chewa	Christian	Farmer	Student	Small business owner
Maternal grandmother alive	0.124*	−0.028	−4.463***	0.01	−0.007	−0.111*	0.150***	−0.046
SE	(0.057)	(0.033)	(0.687)	(0.039)	(0.010)	(0.054)	(0.033)	(0.029)
Cluster wild bootstrap t p values	(0.056)	(0.396)	(0.002)	(0.863)	(0.657)	(0.056)	(0.002)	(0.160)
Paternal grandmother alive	0.071**	−0.004	−2.845***	0.005	0.009	−0.01	0.04	−0.024
SE	(0.032)	(0.029)	(0.563)	(0.023)	(0.012)	(0.036)	(0.024)	(0.027)
Cluster wild bootstrap t p values	(0.028)	(0.871)	(0.002)	(0.853)	(0.569)	(0.739)	(0.104)	(0.370)
Parents siblings alive	−0.004	0	0.044	0.007	0	0.012**	−0.011***	−0.003*
SE	(0.004)	(0.002)	(0.069)	(0.005)	(0.001)	(0.004)	(0.003)	(0.001)
Cluster wild bootstrap t p values	(0.346)	(0.925)	(0.537)	(0.228)	(0.462)	(0.024)	(0.004)	(0.074)
N	881	881	881	881	881	881	881	881

Ordinary Least Squares regressions with baseline characteristics gathered in 2004 as the dependent variable and family networks as independent variables. Sample contains married main respondent mothers present in the second follow-up survey with children born after the intervention began in July 2005. SEs computed using the cluster-correlated Huber-White estimator are reported in parentheses and p values are also reported in parentheses. P values are calculated using the wild cluster bootstrap t procedure described by Cameron *et al*. 37 The wealth index was calculated using principal components analysis as recommended by Filmer and Pritchett.^36^

*p<0.10, **p<0.05, ***p<0.01.

Our main specification is the following linear multivariate regression:

$$\begin{array}{ll} HAZ_{ij}= & \alpha +\beta T_{j}+\beta_{2}Maternal\_grandmother_{ij}+\beta_{3}Maternal\_grandmother_{ij}*T_{j}\\ & +\;\beta_{4}Paternal\_grandmother_{ij}+\beta_{5}Paternal\_grandmother_{ij}*T_{j}\\ & +\;\beta_{6}Total\_mothers\_siblings_{ij}+\beta_{7}Total\_mothers\_siblings_{ij}*T_{j}\\ & +\;\beta_{8}Total\_fathers\_siblings_{ij}+\beta_{9}Total\_fathers\_siblings_{ij}*T_{j}+X_{ij}\gamma \\ & +\;Z_{j}\gamma_{2}+t+\epsilon_{ij} \end{array}$$

where $HAZ_{ij}$ is the height-for-age z-score of child *i* in cluster *j*. *T_j_* is a treatment exposure indicator, which captures whether the child was born to a mother living in 2004 (pre-intervention) in a cluster that was assigned to receive the programme. We therefore use an intent-to-treat estimator. *Maternal_grandmother_ij_* and *Paternal_grandmother_ij_* are binary variables indicating, respectively, whether the maternal and paternal grandmother is alive. $Total\_mothers\_siblings_{ij}$ ($Total\_fathers\_siblings_{ij}$) captures the total number of siblings of the child’s mother (father) who are alive. We use two definitions of this variable in different specifications of the model: (i) brothers and sisters (separately) of each parent and (ii) the total siblings of each parent. *X_ij_* and *Z_j_* are vectors of control variables at the individual and cluster level, respectively. These include all baseline characteristics where significant differences between households with different extended family members alive were detected, and interview month and year indicators to account for month-year-specific shocks. We do not adjust the data for missing information.

We fitted three models, one crude model with the intervention term only, and two full models as specified in the equation each treating parent siblings differently.

The coefficient β captures the effect of the programme for children whose maternal and paternal grandmothers are dead, and whose parents are only children, while the coefficients $\beta_{2}$, $\beta_{4}$, $\beta_{6}$ and $\beta_{8}$, represent the effects of the extended family members on HAZ scores in the control group. The coefficients $\beta_{3}$, $\beta_{5}$, $\beta_{7}$ and $\beta_{9}$, associated with interaction terms between variables capturing extended family relations and the indicator for programme allocation, estimate the additional effect of the programme for children with different types and numbers of extended family members. A positive (or negative) significant interaction provides evidence that the programme effect is enhanced (or diminished) in the presence of that particular family member.

Errors ε_ij_ are assumed to be uncorrelated between individuals in different clusters but are allowed an unrestricted correlation structure within clusters. To account for correlation within clusters, SEs must be adjusted to prevent downward bias, and incorrect inference. Given the small number of clusters in the study (12 intervention and 12 control clusters), we adopt wild cluster bootstrap methods as recommended by Cameron *et al*.^37^ Associated 95% CIs can be calculated using a computationally intensive method suggested by Colin Cameron and Miller.^38^ The bootstrap adjustment applied here was studied in detail by Fitzsimons *et al* and was found to perform well.^17^ Data from both follow-up surveys are pooled to improve statistical power.

The extended family network is defined according to which members of the family are alive, rather than which ones live in the same village or household. This is in case treatment exposure affected decisions over where to live, which would cause a measure of family network size based on residence to be correlated with the intervention and thereby bias estimates. The benefit of defining the size of the family network according to which members are alive is that this is almost certain to be invariant to programme exposure.

We choose to define *T_j_* by exposure to the intervention rather than actual participation since participation in the programme was voluntary and also relied on the ability of peer counsellors to locate eligible women. Women who peer counsellors did not manage to trace or who chose not to take part in the programme may be different from those who did participate. The existence of such systematic differences would potentially introduce some unobserved correlation between the treatment interaction variables and HAZ scores if *T_j_* were defined on the basis of actual participation. Indeed, Fitzsimons *et al* report that women who received the visits tend to be poorer.^17^ Defining treatment based on residence at baseline rather than at the time of the follow-up interviews also alleviates concerns of bias in case there was purposeful migration into treated areas by control-group assigned households.

### Qualitative analysis

Following the findings from the quantitative analysis, we conducted focus group discussions (FGDs) with grandmothers, mothers and peer counsellors, and semi-structured interviews with fathers, CHWs and village chiefs to gain a more in-depth understanding of family roles and how grandmothers might influence child health.

#### Recruitment

Participants were recruited from 11 of 24 intervention and control clusters across the district in late 2015. Mothers, grandmothers and fathers were purposively selected by CHWs and chiefs to represent those households who had actively received the intervention or had children aged under 5 years in control clusters. Volunteer peer counsellors were contacted directly, to represent a range of ages and years’ experience as counsellors. Chiefs and CHWs were purposively selected and contacted directly to represent clusters with a range of engagement with the intervention. We planned to conduct a total of 5 FGDs and 10 interviews, rather than collecting data until saturation was reached.

#### Data collection

FGDs and interviews used topic guides, based on the quantitative findings and wider literature on infant feeding behaviours, covering: household decision making around feeding, infant feeding practices, feeding knowledge and sources of information about infant feeding. We asked about all household members, and specifically probed about the role of grandmothers. All discussions were facilitated by two local trained qualitative researchers in Chichewa. Participants were reimbursed for their travel expenses and given refreshments. All discussions were audio recorded, and then verbatim transcribed in Chichewa. Transcripts were translated into English as a group, with ambiguous terms or phrases debated until a consensus meaning was reached. Data collection, transcription and translation were conducted by EK, HC, TP and FB—female Malawian researchers who are fluent in English.

#### Analysis

The English transcripts were coded using an inductive framework approach based on the following steps: familiarisation, coding, developing and applying the framework, charting and interpretation.^39^ All transcripts were double-coded, as a group by TP, EK, HC and FB and independently by CK—a female British researcher with 5 years’ work experience in Malawi. Coding was done on paper and the coding matrix developed in Microsoft Excel. A round-table discussion was then conducted by all five researchers to compare the codes and agree on themes; disagreements in coding were discussed until an agreement on the interpretation was reached.

#### Patient and public involvement

The original trial was conducted with extensive community engagement, including initial planning and dissemination meetings with village, healthcare and local government committees. These groups were involved in the recruitment of participants for interviews and focus group discussions. The quantitative survey instruments were pretested on households living in buffer areas.

### Ethics

All participants gave informed written consent.

## Results

### Quantitative analysis

Table 4 displays the results of the quantitative analysis on child HAZ scores. Model 1 shows that overall exposure to the programme raised HAZ scores by 0.296 (95% CI 0.116 to 0.484) SD. Models 2 and 3 present the results for the regressions to test whether different family members influenced the effectiveness of the intervention on child HAZ scores. Model 2 presents the results where we allow for brothers and sisters of the child’s parents to have different effects, while model 3 displays those including (separately) the total siblings of each of the child’s parents.

**Table 4:** Estimated effects on height-for-age z-scores with 95% CIs from three linear regression models

	Model 1		Model 2		Model 3	
	Coefficient	95% CI	Coefficient	95% CI	Coefficient	95% CI
Treatment	0.296***	(0.116 to 0.484)	0.441	(−0.335 to 1.028)	0.467	(−0.344 to 1.050)
Maternal grandmother			−0.265**	(−0.528 to 0.021)	−0.259**	(−0.503 to 0.019)
Maternal grandmother*T			0.168	(−0.296 to 0.615)	0.145	(−0.324 to 0.575)
Paternal grandmother			0.008	(−0.200 to 0.236)	0.006	(−0.192 to 0.238)
Paternal grandmother×*T			−0.253*	(−0.529 to 0.029)	−0.235*	(−0.493 to 0.039)
Mothers sisters			0.031	(−0.052 to 0.103)		
Mothers sisters×*T			−0.056	(−0.163 to 0.057)		
Fathers sisters			0.002	(−0.140 to 0.140)		
Fathers sisters*T			0.042	(−0.110 to 0.188)		
Mothers brothers			−0.001	(−0.116 to 0.082)		
Mothers brothers*T			0.024	(−0.109 to 0.170)		
Fathers brothers			0.016	(−0.064 to 0.115)		
Fathers brothers*T			−0.034	(−0.148 to 0.075)		
Total siblings of mother					0.016	(−0.032 to 0.062)
Total siblings of mother×*T					−0.019	(−0.069 to 0.037)
Total siblings of father					0.01	(−0.038 to 0.054)
Total siblings of father×*T					0.001	(−0.080 to 0.072)
R^2^	0.19		0.195		0.193	
N	2017		2017		2017	

OLS regressions with height-for-age (HAZ) scores as dependent variable. Model 1 estimates the overall effect of exposure to the programme. Models 2 and 3 estimate regressions that allow the programme effect to vary with different extended family members. Inference is conducted using the wild cluster bootstrap t procedure recommended by Cameron *et al* ^37^; 95% CIs calculated according to the method recommended by Colin Cameron and Miller.^38^

All regressions include the following controls: cluster level controls: education and Chewa ethnicity in 2004, household level controls: a wealth index calculated in 2004, mother level controls: whether she had completed primary school, was working as a farmer or was a student in 2004, current age, age^2^ and logarithmic height. Child level controls: month of measurement, age, age^2^, gender, number of older siblings, number of older siblings^2^.

Sample includes all children born after the intervention start date in July 2005 to married main respondent mothers, who were aged 0–53 months at the time of measurement. Column 1 indicates the effect of intervention assignment on HAZ scores, for the sample where family networks information is not missing. Models 2–3 indicate how intervention effects on HAZ scores vary with the presence of different extended family members.

*p<0.10, **p<0.05, ***p<0.01.

HAZ, height-for-age z-scores.

For children whose parents have no living mothers or siblings, the effect of the intervention on HAZ scores is between 0.441 (95% CI −0.335 to 1.028) and 0.467 (95% CI −0.344 to 1.050) SD. However, for those children with a living paternal grandmother, the intervention effect was reduced by between 0.235 (95% CI −0.493 to 0.039) and 0.253 (95% CI −0.529 to 0.029) SD. The results also suggest that, in the control group, children whose maternal grandmothers are alive have HAZ scores that are between 0.259 (95% CI −0.0503 to –0.019) and 0.265 (95% CI −0.528 to –0.021) SD lower. The coefficient on the interaction term between having a living maternal grandmother and the indicator for the intervention allocation is positive, but statistically insignificant at the 10% level of significance (magnitude of between 0.145 (95% CI −0.324 to 0.575) and 0.168 (95% CI −0.296 to 0.615) SDs. Finally, the results uncover no association between the number of parents’ siblings on HAZ scores, or of differential effects of the intervention by these.

### Qualitative analysis

We conducted 5 FGDs, with 37 participants of 48 invited (mothers=16; grandmothers=15; peer counsellors=6), and 10 semi-structured interviews (village chiefs=4, fathers=4; CHW=2). We defined the following emergent themes in relation to grandmothers and their role in infant feeding and growth: decision-making roles, knowledge and information, traditional practices and intervention successes and challenges around behaviour change.

#### Decision-making roles

Across the respondents there was agreement that the father is responsible for resource allocation and mobilisation, while the mother’s role is to manage and prepare food for the household. When there is a lack of food or resources, extended family members or neighbours can provide assistance, for example:

maybe you have found you don’t even have flour, our sister in law or mother in law gives it to you saying that it’s only for the child, prepare porridge so that it should eat. (Mother 5, control)

Within the household, grandmothers, both maternal and paternal, were generally viewed as the secondary caregivers, providing support by cooking for and feeding infants.

#### Information and knowledge

Sources of information about infant feeding included: antenatal clinics and other healthcare, family members, village chiefs and community meetings, the peer counsellor intervention and other NGOs and civil society education programmes. Interestingly, the peer counsellors were reported as a source of information by participants from control areas, likely reflecting contamination following the end of the trial period. Although grandmothers report giving similar advice as that given by healthcare workers:

now we are afraid, so we provide the same advice they give at the clinic, so we tell them the same things. (Grandmother 7, intervention)

However, reports from peer counsellors cast doubt on this. They instead mentioned encountering difficulties with grandmothers when disseminating their advice:

frequently the grandmothers mislead, mislead them as they say what they were doing before in their time. (Peer counsellor 5)

Peer counsellors, however, also noticed a change over time in attitudes among grandmothers, with increased acceptance of the intervention messages:

the group of relatives which gives the most problems is the grandparents because they tell the woman that "aaah [the counselors] are just cheating you, they want this child to be crying" […] but we have seen that the grandmothers now have understood. (Peer counsellor 4)

Despite extended family members not being the target group of the intervention, breast feeding, weaning and complementary feeding messages appear to have disseminated, with fathers

advice about breast feeding, I know a lot; when a child is born he should breast feed exclusively, very frequently (Father 1, intervention)

and grandmothers

so they say breast feed frequently these days, that’s the modern way of childbirth, so you also say breast feed the child (Grandmother 2, intervention)

demonstrating accurate knowledge.

#### Traditional practices

Several different traditional practices and beliefs about infant feeding were mentioned by all respondent types, including: adding medicinal herbs to infants’ porridge; believing children become ‘foolish’ if breastfed for too long and smaller portions making children ‘smart’. However, mothers and grandmothers in both intervention and control areas commented that while these practices and beliefs are known to exist, they are no longer commonplace

most of this generation do not follow (these practices) (Mother 8, control)

or rituals and the giving of herbal medicines is done in hiding. This was confirmed by one of the CHW who commented that:

while the grandmothers and the other people have their own beliefs, our role is to get rid of those beliefs […] little by little people change. (CHW 2, intervention)

#### Behaviour change

Community members reported sustained behaviour change relating to exclusive breast feeding and facility-based deliveries:

behaviour these days has changed in that delivering at home is no longer there […] we say go to the hospital. (Grandmother 5, intervention)

However, CHW and peer counsellors noted that these changes were not seen immediately, and that barriers such as lack of engagement, lack of understanding and cultural issues (eg, urban women ‘looking down’ on the counsellors) were present.

## Discussion

Our mixed-methods evaluation of the effect of extended family members on the impact of a peer-led home-education intervention in rural Malawi suggests that living paternal grandmothers can be a barrier to intervention dissemination and behaviour change. The qualitative findings complement the quantitative results, and suggest the mechanism through which grandmothers may influence the effectiveness of the peer intervention.

The apparently negative influence of paternal grandmothers on intervention success may be due to a conflict between their views on infant feeding from the recommendations of peer counsellors. The qualitative findings offer some support for this hypothesis by providing evidence that grandmothers are proponents of ‘traditional’ views of child feeding that differ from standard recommendations, supported by previous studies.^4 12^ They indicate that grandmothers persist in their traditional beliefs for longer, and as providers of both financial and childcare support, exert influence towards their own beliefs of child feeding rather than towards the information provided by the intervention. Reassuringly though, our qualitative data suggest that grandmothers eventually adjust their practices to be in line with the information provided by the intervention.

Interestingly, the qualitative data were not able to distinguish between paternal and maternal grandmothers, despite other evidence that, at least in Malawi, it is paternal grandmothers who command the most influence.^12^ This may explain why we do not find a similar negative effect on intervention success associated with maternal grandmothers in the quantitative analysis. However, our ability to speculate on different mechanisms of action between maternal and paternal grandmothers is limited.

The delays seen in attitude change among grandmothers from the qualitative data suggest that there may be potential to increase the intervention’s impact further by engaging extended family members in the information exchange process. A growing body of evidence underscores the benefits of more inclusive approaches to health education,^12–14 40^ and cautions against assuming that new information will necessarily be incorporated into knowledge and behaviour. Actual response will, in general, depend on the mode of transmission. Approaches that treat users of information as passive are less likely to be effective than those that foster dialogue within the target communities.

In contexts where older women exert particular influence, there are clear grounds for designing interventions that respect and acknowledge their seniority. The rationale behind the work of organisations like the Grandmother Project is that elder women can act as powerful agents for change if they are mobilised and empowered to support intervention aims.^4 12^ We consider our findings to provide support for this agenda. However, it must be noted that involving senior women in interventions might not be sufficient to improve child health, particularly in contexts where poor nutrition is not the only cause for poor health. Evidence from the evaluation of an integrated agriculture and nutrition and health behaviour change communication programme indicates that senior women can be effective in changing knowledge, but this improved knowledge might still fail to yield improvements in child growth.^14^

We uncover a negative association of maternal grandmothers in the control group. However, this cannot be taken as evidence of a causal effect, because of the presence of confounders such as a higher competition for resources in families with living maternal grandmothers in matrilineal societies.^41^

The qualitative findings also raise the importance of the role of men as key providers and resource mobilisers. Previous quantitative evidence by Fitzsimons *et al* 17 supports the critical role of males in ensuring adoption of the information provided. Therefore, integrating these influential figures with the peer counsellor intervention may help improve uptake and reduce the time to intervention acceptance we currently observe.

In this study there were several limitations. First, it is impossible to ascertain whether the estimated quantitative detrimental effect of grandmothers is due to their presence and not because households in which the paternal grandmother is alive are different in some characteristic that is omitted from the regression and that affects the effectiveness of the intervention. The survey and qualitative data may be subject to social desirability bias, with respondents providing answers which they think will please the researchers. As the qualitative and quantitative data triangulated, and respondents were not aware of our hypothesis, we do not feel this considerably biased our conclusions. Finally, the mixed data were collected sequentially rather than concurrently, with the qualitative data collection conducted 5 years after the quantitative survey. This may have resulted in recall bias in the qualitative data, and the culture and behaviours around infant feeding may have shifted between the two study phases. This is somewhat supported by the FGDs and interviews from control areas being exposed to the peer counsellors and their messages, posing a challenge to integrating the results. However, as the qualitative data were planned to provide a more in-depth understanding and triangulation of the quantitative findings, rather than comment on causality, we do not feel this detracts from our interpretation.

We found that paternal grandmothers play an important role in shaping responses to an information campaign targeting infant health. In order to increase the impact of information campaigns, our findings suggest that excluding influential older women, who act as both important sources of advice and childcare support, can weaken intervention impact by exposing a divergence between traditional views and new information. Inclusive health education approaches that respect the need to tackle existing traditional beliefs and the roles that grandmothers play, may overcome this friction and improve the effectiveness of the intervention.

## Supplementary Material

Reviewer comments

Author's manuscript
