# Supplementary figures and images for: Family networks and infant health promotion: a mixed-methods evaluation from a cluster randomised controlled trial in rural Malawi
Source: BMJ Open. 2018 Jun 7;8(6):e019380. doi: 10.1136/bmjopen-2017-019380 (PMC6009513; doi:10.1136/bmjopen-2017-019380)

## Appendix 1

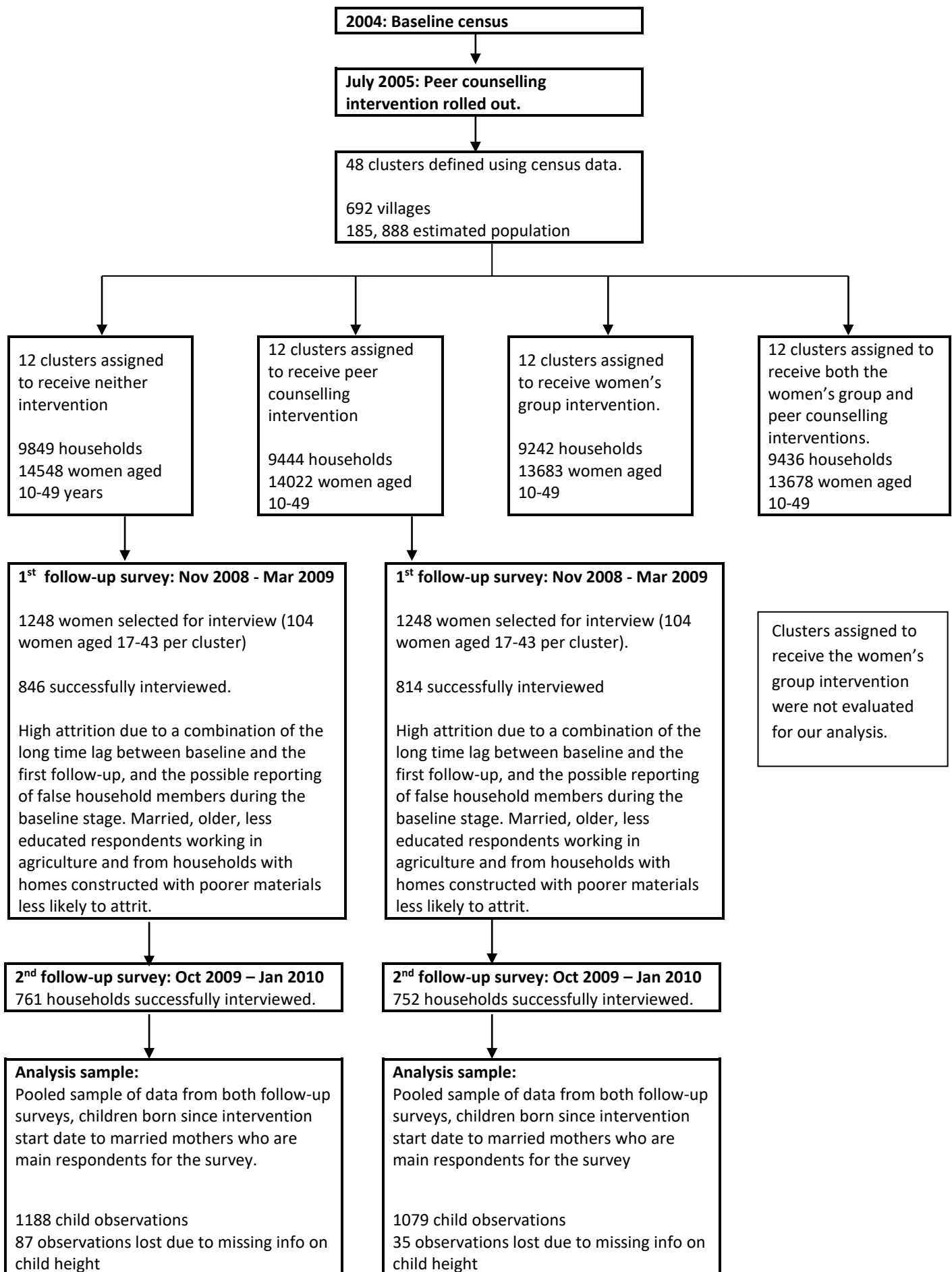

Supplement: Supplementary appendix 1 [file bmjopen-2017-019380supp001.pdf]
