# Supplementary material for: Family networks and infant health promotion: a mixed-methods evaluation from a cluster randomised controlled trial in rural Malawi
Source: BMJ Open. 2018 Jun 7;8(6):e019380. doi: 10.1136/bmjopen-2017-019380 (PMC6009513; doi:10.1136/bmjopen-2017-019380)
Supplement: Author's manuscript [file bmjopen-2017-019380.draft_revisions.pdf]

BMJ Open is committed to open peer review. As part of this commitment we make the peer review history of every article we publish publicly available.

When an article is published we post the peer reviewers' comments and the authors' responses online. We also post the versions of the paper that were used during peer review. These are the versions that the peer review comments apply to.

The versions of the paper that follow are the versions that were submitted during the peer review process. They are not the versions of record or the final published versions. They should not be cited or distributed as the published version of this manuscript.

BMJ Open is an open access journal and the full, final, typeset and author-corrected version of record of the manuscript is available on our site with no access controls, subscription charges or pay-per-view fees (http://bmjopen.bmj.com).

If you have any questions on BMJ Open's open peer review process please email info.bmjopen@bmj.com

Family networks and infant health promotion: a mixed-methods evaluation from a cluster randomised controlled trial in rural Malawi

Journal:	*BMJ Open*
Manuscript ID	bmjopen-2017-019380
Article Type:	Research
Date Submitted by the Author:	29-Aug-2017
Complete List of Authors:	Scott, Molly; Oxford Policy Management<br>Malde, Bansi; University of Kent, School of Economics; Insitute for Fiscal Studies, Centre for Evaluation of Development Policies<br>King, C.; UCL, Institute for Global Health<br>Phiri, Tambosi; MaiMwana Project,<br>Chapota, Hilda; MaiMwana Project,<br>Kainja, Esther; MaiMwana Project,<br>Banda, Florida; MaiMwana Project<br>Vera-Hernandez, Marcos; UCL; Insitute for Fiscal Studies, Centre for Evaluation of Development Policies
**Primary Subject Heading**:	Global health
Secondary Subject Heading:	Health policy
Keywords:	Nutrition < TROPICAL MEDICINE, Extended Family, Health promotion, Sub-Saharan Africa, Child Health

SCHOLARONE™
Manuscripts

For peer review only - http://bmjopen.bmj.com/site/about/guidelines.xhtml

Family networks and infant health promotion: a mixed-methods evaluation from a cluster randomised controlled trial in rural Malawi

Authors:

Molly Scott[1], Bansi Malde[3,6]*, Carina King[4], Tambosi Phiri[5], Hilda Chapota[5], Esther Kainja[5], Florida Banda[5], Marcos Vera-Hernandez[2,3]

Affiliations:

1. Oxford Policy Management
2. Department of Economics, University College London, UK
3. Centre for Evaluation of Development Policies, Institute for Fiscal Studies, London, UK
4. Institute for Global Health, University College London, UK
5. MaiMwana Project, Mchinji, Malawi
6. School of Economics, University of Kent, UK

***Corresponding author:**

School of Economics, University of Kent, Canterbury, Kent CT2 7NZ, UK. E-mail: b.k.malde@kent.ac.uk; Telephone: (+44)(0)1227816464

Keywords: Extended Family; Child Health; Sub-Saharan Africa; Health Promotion; Child Nutrition

For peer review only - http://bmjopen.bmj.com/site/about/guidelines.xhtml

Abstract:

Objective: Parents may rely on information provided by extended family members when making decisions concerning the health of their children. We therefore aimed to evaluate whether the success of an information intervention promoting infant health was affected by extended family members.

Methods: The intervention was implemented as a cluster randomised controlled trial in Mchinji district, Malawi (24 clusters total). 12 clusters were assigned to the intervention, in which all pregnant women and new mothers were eligible to receive 5 home visits from a trained peer counsellor to discuss infant care and nutrition practices; 12 clusters served as controls. We used linear multivariate regression to test whether the intervention impact on child height-for-age z-scores was influenced by the presence of extended family members. We conducted focus group discussions with mothers, grandmothers and peer counsellors, and key-informant interviews with husbands, chiefs and community health workers to gain a better understanding of the roles of extended family members in infant feeding.

Results: Exposure to the intervention increased child height-for-age z-scores by an average of 0.296 standard deviations (p=0.002). However the effect size was between 0.235 (p=0.088) and 0.253 (p=0.058) standard deviations lower if the paternal grandmother is still alive. There was no effect of parents' siblings. Maternal grandmothers did not affect intervention impact, but were associated with a reduction in child height in the absence of the intervention. Qualitative analysis suggested that grandmothers, who act as secondary care-givers and provide resources for infants, were slower to dismiss traditionally held practices of infant feeding and adopt new messages provided by the intervention.

Conclusion: Overall our results point to a highly influential role for grandmothers on child health in this region. We suggest that success of the intervention could be increased if it sought to integrate senior women rather than focusing exclusively on mothers.

For peer review only - http://bmjopen.bmj.com/site/about/guidelines.xhtml

Strengths and Limitations:

- Uses mixed methods to understand how extended family members affected the success of an infant promotion program in Malawi
- Linear multivariate regressions test whether the impact of the intervention, which was implemented as a cluster randomised control trial, is influenced by different extended family members
- Quantitative analysis allows different extended family members – paternal grandmothers, maternal grandmothers, among others – to have different impacts on the program's success
- Focus group discussions and key informant interviews help shed light on the mechanisms through which extended family members might have affected the intervention's success

For peer review only

For peer review only - http://bmjopen.bmj.com/site/about/guidelines.xhtml

Introduction

Child health outcomes are influenced by individuals besides the mother and father, with a rich literature devoted to the contribution of extended family members. In low-income settings, where risk for poor health is high and social welfare nets minimal, the support of the extended family may be crucial in child-rearing [1]. Relatives can assist by acting as secondary caregivers [2], supplying labour, donating money and providing other in-kind resources to the household [3], [4], [5], [6], [7]. Older women in the family can also influence child outcomes by dispensing infant health and nutrition advice to new mothers [8], [9], [10], [11].

Extended family members may impact on the effectiveness of policies and interventions to improve child health in low-income settings. On the one hand, they may provide resources and support that complement the intervention making it more effective, and on the other they could be resistant to change and reinforce traditional practices, thereby undermining interventions. The latter is particularly relevant in the case of health education and outreach programs, which are widespread across both developed and developing country settings.

Educational programmes have the potential to improve child health outcomes by changing widespread misconceptions or traditional behaviours around child feeding and care in low-income settings. In Malawi, for instance, although most infants are breastfed for at least a year, only 40.5% of infants are still exclusively breastfed at 5 months [12]; while diets of children aged over 6 months usually lack sufficient diversity [13],[14].

Education campaigns promoting the adoption of certain health behaviours have shown mixed success in developing country settings. While they have led to sustained increases in targeted health practices, such as exclusive breastfeeding [16], complementary feeding [11], hand washing [17] [18] and the use of a non-contaminated water source [19]; information campaigns on their own have had a negligible effect on behaviour and health in the case of worm infections in Kenya

For peer review only

For peer review only - http://bmjopen.bmj.com/site/about/guidelines.xhtml

[20];and anaemia prevention in China [21]. There is hence a need for greater understanding of the factors shaping responses to health education campaigns across contexts.

Little attention has been paid to the role of extended family members in influencing the success of health education interventions in the existing literature, despite their important role in shaping child health.

A cluster randomised controlled trial conducted in Malawi used a peer home-education strategy to improve rates of exclusive breastfeeding, and reduce infant mortality (see Lewycka et al. 2010, 2013 for further details [26], [25]). The trial demonstrated a 36% reduction in infant mortality and an improvement in children's height-for-age z-score (HAZ) (increased by 0.271 standard deviations; p=0.022) [13] [25]. This paper uses mixed-methods to investigate whether members of the extended family influenced the success of this peer home visiting intervention, and the possible mechanisms through which their influence might work.

Methods:

This is a secondary, sequential mixed-methods study based on a cluster RCT of a peer home-education intervention conducted in Mchinji District, Malawi. We investigate family member roles in the success of the intervention, which provided information on infant feeding, with quantitative data collected between November 2008 – January 2010, and qualitative data collected in December 2015. Full details of the original trial and methods have previously been published [26] [25].

Setting:

Mchinji is a rural district in central Malawi with a population of about 455,000 [27]. Maternal and infant healthcare is delivered at one district hospital, four rural hospitals, nine health centres, private clinics, and in the community through government employed community health workers (CHW – known locally as Health Surveillance Assistants). Much of the healthcare received by

For peer review only - http://bmjopen.bmj.com/site/about/guidelines.xhtml

pregnant women and infants is in the community setting by CHWs, or at home by kin and other social contacts. The 2010 Malawi Demographic and Health Survey reported 24% of births in the region occurred in the woman's own home and 2% in someone else's home, and many births are not attended by a medically trained healthcare personnel but by traditional birth attendants (14.4%), friends and relatives (8.7%) or no one (2.6%) [12]. Without access to a skilled birth attendant, women are more vulnerable to infection and complications during birth; the infant mortality rate in 2010 was of 66 per 1000 livebirths [12].

For the RCT, Mchinji was divided into 48 approximately equal population clusters based on the 1998 Malawi Population and Housing census (the most recent census at the time of trial planning). Within each cluster of around 8000 people, the 3000 individuals living in villages closest to the geographical centre were enumerated as the eligible study population. 12 clusters were assigned to the infant feeding intervention only and 12 served as controls. Full details of the trial set-up and methods are in Lewycka et al., 2010 [26]. All women living in clusters assigned to the infant feeding intervention who became pregnant during the trial period were eligible to receive five home visits from a trained local woman volunteer ('peer counsellor') to discuss maternal and infant healthcare issues; around 60% of eligible women report having been visited. The visits were timed to coincide with key stages of infant development (the third trimester, and at one week, one month, three and five months after birth). Each visit focused on a specific set of topics for discussion, with special attention paid to nutrition practices including exclusive breastfeeding, and complementary feeding. Peer counsellors were literate local women aged 23-50 years with breastfeeding experience, who covered a population of about 1000 people.

The intervention began in December 2004, with an initial establishment period until June 2005. The trial was on-going at the time of the quantitative data collection. Following the end of the trial period, peer counsellors continued to receive mentorship and supervision support from government CHWs and the local implementing NGO. In 2015, at the time of qualitative data

For peer review only - http://bmjopen.bmj.com/site/about/guidelines.xhtml

collection, approximately 1/3 of the volunteer counsellors were still active in delivering the intervention.

Quantitative Analysis:

<u>Data and Sample Selection:</u>

A baseline census was conducted in all clusters in 2004, prior to the start of the intervention. All women aged between 10 and 49 were enumerated and a random sample of 104 women aged between 17 and 43 per cluster was then drawn to be interviewed for two follow-up quantitative surveys as part of this secondary study. Sampled women (`main respondent' hereon) were visited to complete the first follow-up in November 2008-March 2009, and a second follow-up from October 2009 –January 2010.

Each follow-up survey contained questions about the size of the extended family of the main respondent and her husband (those alive and those in the village), the health of all household members, food and liquid intake of children aged under 6 years, knowledge about child nutrition, intervention participation (in treatment clusters), and socio-economic variables such as adult work. The height of the main respondent and the height and weight of children under 6 years was also collected.

The sample was balanced between treatment and control clusters along a range of variables collected at baseline (see Fitzsimons et al (2016), Table 1 [13]). Balance was preserved after accounting for attrition between the baseline and first endline survey, indicating that randomisation was not jeopardised.

For the analysis we use a sample of children who were born since July 2005; and whose mothers are married main respondents in the follow-up surveys (80% of the sample). This sample selection ensures that we measure effects on children whose mothers were eligible to receive visits from a

For peer review only - http://bmjopen.bmj.com/site/about/guidelines.xhtml

peer counsellor; and allows us to compare effects of the mothers' relatives with those of her husband.

Model Specification and Estimation

The quantitative analysis aims to determine how different family members influence the effectiveness of the infant feeding intervention. The main outcome for our analysis is child height-for-age z-score (HAZ score), which is a long term indicator of health that reflects nutrition and morbidity since birth, and should be sensitive to any effects of intervention exposure in early life. It is calculated by comparing the height of the child with the median height in the WHO reference population of children of the same gender and age in months [28].

The main specification is estimated using the following linear multivariate regression:

$$\begin{aligned} HAZ_{ij} = \alpha + \beta T_j + \beta_2 Maternal\_grandmother_{ij} + \beta_3 Maternal\_grandmother_{ij}{}^{*} T_j \\ + \beta_4 Paternal\_grandmother_{ij} + \beta_5 Paternal\_grandmother_{ij}{}^{*} T_j \\ + \beta_6 Total\_mothers\_siblings_{ij} + \beta_7 Total\_mothers\_siblings_{ij}{}^{*} T_j \\ + \beta_8 Total\_fathers\_siblings_{ij} + \beta_9 Total\_fathers\_siblings_{ij}{}^{*} T_j + X_{ij}\gamma \\ + Z_j \gamma_2 + \epsilon_{ij} \end{aligned}$$

T_j is a treatment exposure indicator which captures whether the child was living in a cluster that was assigned in 2004 to receive the program. We therefore use an intent-to-treat estimator. $Maternal\_grandmother_{ij}$ and $Paternal\_grandmother_{ij}$ are binary variables indicating, respectively, whether the maternal and paternal grandmother are alive. $Total\_mothers\_siblings_{ij}$ captures the total number of siblings of the child's mother who are alive. The exact definition of this variable varies across specifications. In the first specification, brothers and sisters of each parent enter the regression equation separately, while the second specification includes the total siblings of each parent. X_{ij} and Z_j are vectors of control variables at the individual (denoted i) and cluster level (denoted j) respectively. Among the controls we include are all variables where significant

For peer review only - http://bmjopen.bmj.com/site/about/guidelines.xhtml

differences between households with different extended family members alive were detected in Table 3. We do not adjust the data for missing information.

Errors ε_{ij} are assumed to be uncorrelated between individuals in different clusters but are allowed an unrestricted correlation structure within clusters. To account for correlation within clusters, standard errors must be adjusted to prevent downward bias. Given the small number of clusters included in the study design (12 intervention and 12 control clusters), we adopt wild cluster bootstrap methods as recommended in Cameron et al. (2008) [29]. The bootstrap adjustment applied here was studied in detail by Fitzsimons et al (2016) and was found to perform well [13]. Data from both follow-up surveys is pooled to improve statistical power.

The extended family network is defined according to which members of the family are alive, rather than which ones live in the same village or household. This is in case treatment exposure affected decisions over where to live. The benefit of defining the size of the family network according to which members are alive is that this is almost certain to be invariant to program exposure.

We choose to define T_j by exposure to the intervention rather than actual participation since participation in the program was voluntary and also relied on the ability of peer counsellors to locate eligible women. Women who peer counsellors did not manage to trace or who chose not to take part in the program may be different from those who did participate, potentially introducing some unobserved correlation between the treatment interaction variables and HAZ scores if T_j were defined on the basis of actual participation. Indeed, Fitzsimons et al. (2016) report that women who received the visits tend to be poorer. Defining treatment based on residence at baseline rather than at the time of the follow-up interviews also alleviates concerns of bias in case there was purposeful migration into treated areas by control-group assigned households.

For peer review only

For peer review only - http://bmjopen.bmj.com/site/about/guidelines.xhtml

Qualitative Analysis

To gain a more in-depth understanding of family roles and how grandmothers might influence child health, we conducted focus group discussions (FGDs) with grandmothers, mothers and peer counsellors, and semi-structured interviews with fathers, CHWs and village chiefs in late 2015.

Recruitment

Participants were recruited from 11 of 24 intervention and control clusters across the district. Mothers, grandmothers and fathers were purposefully selected by CHWs and chiefs to represent those households who had actively received the intervention or had children under-5 years in control clusters. Volunteer peer counsellors were contacted directly, to represent a range of ages and years' experience as counsellors. Chiefs and CHWs were purposefully selected and contacted directly to represent clusters with a range of engagement with the intervention.

Data Collection:

FGDs and interviews covered the following topics: household decision making around feeding, infant feeding practices, feeding knowledge and sources of information about infant feeding. We asked about all household members, and specifically about the role of grandmothers. All discussions were facilitated by two local trained qualitative researchers in Chichewa within the communities. Participants were reimbursed for their travel expenses and given refreshments. All discussions were recorded, and translated and transcribed by TP, EK, HC and FB as a group; final translations agreed between the researchers.

Analysis

The English transcripts were coded using an inductive framework approach based on the following steps: familiarisation, coding, developing and applying the framework, charting and interpretation [30]. The transcripts were coded as a group by TP, EK, HC and FB and

For peer review only - http://bmjopen.bmj.com/site/about/guidelines.xhtml

For peer review only

independently by CK; this was done on paper and the coding matrix developed in Microsoft Excel. The framework and interpretation was agreed through discussion between TP, EK, HC, FB and CK until agreement was reached between the researchers.

Ethics

All participants gave informed written consent. Ethical approval to conduct this study was obtained from the National Health Sciences Research Committee in Malawi [Protocol Numbers: 491; 15/9/1483].

Results

Quantitative Analysis:

Table 1 presents basic demographic and socio-economic information about women included in the quantitative analysis and their households at baseline. Amongst those assigned to the control group, the average age was 24.6 years, 71.8% were married and while 70.1% had completed at least primary education, only 7.6% had completed secondary education. In line with the general profile of communities in Mchinji, 95.4% of women in the sample were Chewa ethnicity and 98.3% were Christian. The average household size was 5.6 members and all households were engaged in agricultural activity.

Table 2 presents information from both follow-up surveys about the size of extended family networks of the children included in our analysis sample. It shows that most children have their grandmothers alive (87.3% have maternal grandmothers alive and 80.7% have paternal grandmothers). Moreover, their parents have a relatively large number of siblings, with an average of more than two brothers and two sisters each.

In Table 3 we report the results of a series of linear multivariate regressions which compare the baseline characteristics of mothers and their households according to which members of the

For peer review only - http://bmjopen.bmj.com/site/about/guidelines.xhtml

extended family are alive. It indicates that children whose grandmothers are alive have on average younger mothers, who are more likely to have completed at least primary education, less likely to be working as farmers in 2004, and are from more socioeconomically advantaged households, as measured by a composite wealth index.

Table 4 displays the results of the quantitative analysis on child HAZ scores. Column 1 shows that overall exposure to the program raised HAZ scores by 0.296 (p=0.002) standard deviations (SD). Columns 2 and 3 present the results for the regressions that test whether family members influenced the effectiveness of the intervention on child HAZ scores. The results show that the effect of the intervention is not heterogeneous according to the number of parents' siblings. However, the effect of the intervention on HAZ scores is between 0.235 (p=0.058) and 0.253 (p=0.088) SD smaller for children whose paternal grandmother is alive. The results suggest that maternal grandmothers are associated with lower child health outcomes in the absence of the intervention.

<u>Qualitative Analysis:</u>

We conducted five FGDs, with 37 participants of 48 invited (mothers=16; grandmothers=15; peer counsellors=6), and ten semi-structured interviews (village chiefs=4, fathers=4; CHW=2). We defined the following emergent themes in relation to grandmothers and their role in infant feeding and growth: decision making roles, knowledge and information, traditional practices and intervention successes and challenges around behaviour change.

Decision-Making Roles: Across the respondents there was agreement that the father is responsible for resource allocation and mobilisation, while the mother's role is to manage and prepare food for the household. When there is a lack of food or resources, extended family members or neighbours can provide assistance, for example "*maybe you have found you don't even have flour, our sister in law or mother in law gives it to you saying that it's only for the child, prepare porridge so that it*

For peer review only - http://bmjopen.bmj.com/site/about/guidelines.xhtml

should eat" (Mother, Control). Within the household, grandmothers, both maternal and paternal, were generally viewed as the secondary caregivers, providing support by cooking for and feeding infants.

Information and Knowledge: Sources of information about infant feeding included: antenatal clinics and other healthcare, family members, village chiefs and community meetings, the peer counsellor intervention and other NGOs and civil society education programmes. Interestingly, the peer counsellors were reported as a source of information by participants from control areas, likely reflecting contamination following the end of the trial period. Although grandmothers report giving similar advice as that given by healthcare workers:

"*now we are afraid, so we provide the same advice they give at the clinic, so we tell them the same things*" (Grandmother, Intervention),

However, reports from peer counselors cast doubt on this. They instead mentioned encountering difficulties with grandmothers when disseminating their advice: "*frequently the grandmothers mislead, mislead them as they say what they were doing before in their time*" (Peer Counselor).

Peer counselors, however, also noticed a change over time in attitudes among grandmothers, with increased acceptance of the intervention messages: "*the group of relatives which gives the most problems is the grandparents because they tell the woman that 'aaah [the counselors] are just cheating you they want this child to be crying' [...] but we have seen that the grandmothers now have understood*" (Peer Counselor).

Despite not being the focus of the intervention, breastfeeding, weaning and complementary feeding messages appear to have disseminated among the extended family, with fathers ("*advice about breast feeding, I know a lot; when a child is born he should breast feed exclusively, very frequently*" - Father, Intervention) and grandmothers ("*so they say breastfeed frequently these days, that's the*

For peer review only - http://bmjopen.bmj.com/site/about/guidelines.xhtml

modern way of childbirth, so you also say breastfeed the child" - Grandmother, Intervention) demonstrating accurate knowledge.

Traditional Practices: Traditional practices, such as adding traditional medicinal herbs to infants' porridge, were mentioned in relation to infant health and feeding. However, mothers and grandmothers in both intervention and control areas commented that while these practices are known, they are no longer commonplace or are done in hiding. This was confirmed by one of the CHW who commented that: "*while the grandmothers and the other people have their own beliefs, our role is to get rid of those beliefs […] little by little people change*" (CHW, Intervention).

Behaviour Change: Community members reported sustained behaviour change relating to exclusive breastfeeding and facility-based deliveries: "*behaviour these days has changed in that delivering at home is no longer there [...] we say go to the hospital*" (Grandmother, Intervention). However, CHW and peer counselors noted that these changes were not seen immediately, and that barriers such as lack of engagement, lack of understanding and cultural issues (e.g. urban women 'looking down' on the counselors) were present.

Discussion:

Our mixed-methods evaluation of the effect of extended family members on the impact of a peer-led home education intervention in rural Malawi suggests that living grandmothers can be a barrier to intervention dissemination and behaviour change. The qualitative findings complement the quantitative results, and suggest the mechanism through which grandmothers may influence the effectiveness of the peer intervention.

The apparently negative influence of paternal grandmothers on intervention success may be due to a conflict between their views on infant feeding from the recommendations of peer counsellors. The qualitative findings offer some support for this hypothesis by providing evidence that grandmothers are proponents of traditional views of child feeding that differ from standard

For peer review only - http://bmjopen.bmj.com/site/about/guidelines.xhtml

recommendations, supported by previous studies [9], [8].They indicate that grandmothers persist in their traditional beliefs for longer, and as providers of both financial and childcare support, exert influence towards their own beliefs of child feeding rather than towards the information provided by the intervention. Reassuringly though, our data suggest that grandmothers eventually adjust their practices to be in line with the information provided by the intervention.

Though the qualitative information did not show any distinction between paternal and maternal grandmothers, there is evidence that, at least in Malawi, it is paternal grandmothers who command the most influence [8]. This may explain the fact that we do not see a similar quantitative result associated with maternal grandmothers.

The delays seen in attitude change amongst grandmothers suggest that there may be potential to increase the intervention's impact further by engaging extended family members in the information exchange process. A growing body of evidence underscores the benefits of more inclusive approaches to health education [31], [8], [10], [11], and cautions against assuming that new information will necessarily be incorporated into knowledge and behaviour. Actual response will, in general, depend on the mode of transmission. Approaches which treat users of information as passive are less likely to be effective than those which foster dialogue within the target communities.

In contexts where older women exert particular influence there are clear grounds for designing interventions that respect and acknowledge their seniority. The rationale behind the work of organisations like the Grandmother Project is that elder women can act as powerful agents for change if they are mobilised and empowered to support intervention aims [9], [8]. We consider our findings to provide support for this agenda. However, it must be noted that involving senior women in interventions might not be sufficient to improve child health, particularly in contexts where poor nutrition is not the only cause for poor health. Evidence from the evaluation of an integrated agriculture and nutrition and health behaviour change communication programme

For peer review only - http://bmjopen.bmj.com/site/about/guidelines.xhtml

indicates that senior women can be effective in changing knowledge, but this improved knowledge might still fail to yield improvements in child growth [11].

The qualitative findings also raise the importance of the role of men as key providers and resource mobilisers. Quantitative evidence found in Fitzsimons et al (2016) supports the critical role of males in ensuring adoption of the information provided. Therefore, integrating these influential figures with the peer counsellor intervention may help improve uptake and reduce the time to intervention acceptance we currently observe.

In this study there were several limitations. Firstly, it is impossible to ascertain whether the estimated quantitative detrimental effect of grandmothers is due to their presence and not because households in which the paternal grandmother is alive are different in some characteristic that is omitted from the regression and that affects the effectiveness of the intervention. The survey and qualitative data may be subject to social desirability bias, with respondents providing answers which they think will please the researchers. As the qualitative and quantitative data triangulated, and respondents were not aware of our hypothesis, we do not feel this considerably biased our conclusions. Finally, the qualitative and quantitative data were collected sequentially rather than concurrently, making interpretation more challenging. The qualitative data was planned to provide a more in-depth understanding of the quantitative findings, rather than drawing conclusions on causality, therefore we do not feel this detracts from the interpretation.

We found that grandmothers play an important role in shaping responses to an information campaign targeting infant health. In order to increase the impact of information campaigns, our findings suggest that excluding influential older women who act as both important sources of advice and childcare support, can weaken intervention impact by exposing a divergence between traditional views and new information. Inclusive health education approaches which respect the

For peer review only - http://bmjopen.bmj.com/site/about/guidelines.xhtml

need to tackle existing traditional beliefs and the roles which grandmothers play, may overcome this friction and improve the effectiveness of the intervention.

For peer review only

For peer review only - http://bmjopen.bmj.com/site/about/guidelines.xhtml

Acknowledgements: We thank Andrew Mganga, Nicholas Mbwana, Christopher Kamphinga, Sonia Lewycka, Mikey Rosato and Julia Behrman for advice and assistance with quantitative data collection.

Funding: Funding from the ESRC/DFID grant ES/J009253/1 if gratefully acknowledged.

Conflict of interest: We declare that we have no conflicts of interest.

Authors contributions: All authors provided final approval of the version to be published. Concept and design of quantitative analysis: MS, BM and MV; analysis and interpretation of quantitative findings: MS, MV and BM; drafting the article and revising it critically: MS, BM, CK and MV. Concept and design of the qualitative study: BM, MVH, CK and TP. Qualitative data collection and analysis: TP, HC, EK, FB. Qualitative analysis was independently checked and coded: CK. CK, TP, EK, HC and FB discussed and agreed the final interpretation.

Data sharing: The raw quantitative data is available for download from the UK Data Archive.

For peer review only

References

[1] S. B. Hrdy, “Mother nature: A history of mothers, infants, and natural selection.,” *New York,* 1999.

[2] P. K. Ivey, “Cooperative Reproduction in Ituri Forest Hunter-Gatherers: Who Cares for Efe Infants? 1.,” *Current Anthropology, 41.5,* pp. 856-866, 2000.

[3] M. A. Gibson and R. Mace, “Helpful Grandmothers in rural Ethiopia: A study of the effect of kin on child survival and growth,” *Evolution and Human Behaviour 26(6),* pp. 469-482, 2005.

[4] R. Lee and K. Kramer, “Children's Economic Roles in the Maya Family Life Cycle: Cain, Caldwell, & Chayanov Revisited,” *Population and Development Review, Vol. 28, No. 3,* pp. 475-499, 2002.

[5] M. Angelucci, G. De Giorgi, M. Rangel and I. Rasul, “Family networks and school enrolment: Evidence from a randomised social experiment.,” *Journal of Public Economics, 94(3),* pp. 197-221, 2010.

[6] D. LaFave and D. Thomas, “Extended Families and Child Well-being,” *NBER Working Paper Series, Working Paper 20702, http://www.nber.org/papers/w20702,* 2014.

[7] E. Duflo, “Grandmothers and Granddaughters: Old-Age Pensions and Intrahousehold Allocation in South Africa,” *World Bank Economic Review,* vol. 17, no. 1, pp. 1-25, 2003.

[8] R. Bezner-Kerr, L. Dakishoni, L. Shumba, R. Msachi and M. Chirwa, “"We grandmothers know plenty": breastfeeding, complementary feeding and the multifaceted role of grandmothers in Malawi.,” *Social Science & Medicine, 66(5),* pp. 1095-1105, 2008.

[9] J. Aubel, I. Touré and M. Diagne, “Senegalese grandmothers promote improved maternal and child nutrition practices: the guardians of traditional are not averse to change.,” *Social Science & Medicine, 59(5),* pp. 945-959, 2004.

[10] J. Aubel and D. Sihalathavong, “Participatory communication to strengthen to role of grandmothers in child health: an alternative paradigm for health education and health communication.,” *Journal of International Communication, 7(2),* pp. 76-97, 2001.

[11] D. K. Olney, A. Pedehombga, M. T. Ruel and A. Dillon, “A 2-Year Integrated Agriculture and Nutrition and Health Behavior Change Communication Program Targeted to Women in Burkina Faso Reduces Anemia, Wasting and Diarrhea in Children 3-12.9 Months of Age at Baseline: A Cluster-Randomized Controlled Trial,” *The Journal of Nutrition,* vol. 145, no. 6, pp. 1317 - 1324, 2015.

[12] National Statistical Office, Malawi, Macro ORC, “Malawi Demographic and Health Survey,” Zomba: Malawi National Statistical Office, Macro ORC, 2004.

For peer review only - http://bmjopen.bmj.com/site/about/guidelines.xhtml

[13] E. Fitzsimons, B. Malde, A. Mesnard and M. Vera-Hernandez, “Nutrition, Information, and Household Behaviour: Experimental Evidence from Malawi,” *Journal of Development Economics,* vol. 122, pp. 113-126, 2016.

[14] R. Haider, A. Ashworth, I. Kabir and S. R. Huttly, “Effect of community-based peer counsellors on exclusive breastfeeding practices in Dhaka, Bangladesh: a randomised controlled trial.,” *The Lancet, 356(9242),* pp. 1643-1647, 2000.

[15] B. F. Kalanda, F. H. Verhoeff and B. J. Brabin, “Breast and complementary feeding practices in relation to morbidity and growth in Malawian infants.,” *European journal of clinical nutrition, 60(3),* pp. 401-407, 2006.

[16] A. L. Morrow, M. L. Guerrero, J. Shults, J. Calva, C. Lutter, J. Bravo, G. Ruiz-Palacios, R. Morrow and F. Butterfoss, “Efficacy of home-based peer counselling to promote exclusive breastfeeding: a randomised controlled trial.,” *The Lancet, 353(9160),,* pp. 1226-1231, 1999.

[17] S. Cairncross, K. Shordt, S. Zacharia and B. K. Govindan, “What causes sustainable changes in hygiene behaviour? A cross-sectional study from Kerala, India.,” *Social Science & Medicine, , 61(10),* pp. 2212-2220, 2005.

[18] A. Bowen, H. Ma, J. Ou, W. Billhimer, T. Long, E. Mintz, R. M. Hoekstra and S. Luby, “A cluster-randomized controlled trial evaluating the effect of a handwashing-promotion program in Chinese primary schools.,” *The American journal of tropical medicine and hygiene, 76(6),* pp. 1166-1173, 2007.

[19] M. Madajewicz, A. Pfaff, A. van Geen, J. Graziano, I. Hussein, H. Momotaj, R. Sylvi and H. Ahsan, “Can information alone change behavior? Response to arsenic contamination of groundwater in Bangladesh.,” *Journal of Development Economics, 84(2),* pp. 731-754, 2007.

[20] E. Miguel and M. Kremer, “The Illusion of Sustainability,” *The Quarterly journal of economics, 122(3),* pp. 1007-1065, 2007.

[21] R. Luo, Y. Shi, Z. L., H. Zhang, G. Miller, A. Medina and S. Rozelle, “he limits of health and nutrition education: evidence from three randomized-controlled trials in rural China,” *CESifo Economic Studies, 58(2),* pp. 385-404, 2012.

[22] P. Dupas, “ Health behavior in developing countries.,” *Annual Review of Economics, 3(1) ,* pp. 425-449, 2011.

[23] J. Meredith, J. Robinson, S. Walker and B. Wydick, “Keeping the doctor away: Experimental evidence on investment in preventative health products,” *Journal of Development Economics, 105,* pp. 196-210, 2013.

[24] H. Moestue, S. Huttly, L. Sarella and S. Galeb, “'The bigger the better' - mothers' social networks and child nutrition in Andhra Pradesh,” *Public health nutrition 10(11),* pp. 1274-1282, 2007.

For peer review only - http://bmjopen.bmj.com/site/about/guidelines.xhtml

[25] S. Lewycka, C. Mwansambo, M. Rosato, P. Kazembe, T. Phiri, A. Mganga, H. Chapota, F. Malamba, E. Kainja, M. Newell, G. Greco, A. Pulkki-Brännström, J. Skordis-Worrall, S. Vergnano, D. Osrin and A. Costello, "Effect of women's groups and volunteer peer counselling on rates of mortality, morbidity, and health behaviours in mothers and children in rural Malawi (MaiMwana): a factorial, cluster-randomised controlled trial.," *The Lancet, 381(9879),* pp. 1721-1735, 2013.

[26] S. Lewycka, C. Mwansambo, P. Kazembe, T. Phiri, A. Mganga, M. Rosato, H. Chapota, F. Malamba, S. Vergnano, M. Newell, D. Orsin and A. Costello, "A cluster randomised controlled trial of the community effectiveness of two interventions in rural Malawi to improve health care and to reduce maternal, newborn and infant mortality," *Trials, 11(1), 88,* 2010.

[27] National Statistical Office, Malawi, "National Population and Housing Census: Analytical Report," 1998.

[28] WHO Anthro for personal computers, version 3.2.2, *Software for assessing growth and development of the world's children.,* Geneva: WHO, 2011.

[29] A. C. Cameron, J. B. Gelbach and D. L. Miller, "Bootstrap-based improvements for inference with clustered errors.," *The Review of Economics and Statistics, 90(3),* pp. 414-427, 2008.

[30] N. K. Gale, G. Heath, E. Cameron, S. Rashid and S. Redwood, "Using the Framework Method for the Analysis of Qualitative Data in Multi-Disciplinary Health Research," *BMC Medical Research Methodology,* vol. 13:117, 2013.

[31] R. G. Lee and T. Garvin, "Moving from information transfer to information exchange in health and health care.," *Social science & medicine, 56(3),* pp. 449-464, 2003.

[32] R. Sear and D. Coall, "How much does family matter? Cooperative breeding and the demographic transition.," *Population and Development Review,* pp. 81-112, 2011.

[33] W. D. Hamilton, "The moulding of senescence by natural selection.," *Journal of theoretical biology, 12(1),* pp. 12-45, 1966.

[34] K. Hawkes, "Grandmothers and the evolution of human longevity.," *American Journal of Human Biology, 15(3),* pp. 380-400, 2003.

[35] R. Sear, "Kin and child survival in rural Malawi," *Human Nature, 19(3),* pp. 277-293, 2008.

[36] G. Reniers and R. Tfaily, "Polygyny and HIV in Malawi.," *Demographic Research, 19(53),* p. 1811, 2008.

[37] T. Takane, "Tobacco and smallholders in Malawi: village case studies in the Mchinji and Mangochi Districts.," *African Research Series, 93-119.,* 2005.

[38] S. A. West, I. Pen and A. S. Griffin, "Cooperation and competition between relatives.," *Science,*

For peer review only

For peer review only - http://bmjopen.bmj.com/site/about/guidelines.xhtml

296(5565), pp. 72-75, 2002.

[39] R. Bezner-Kerr, P. R. Berti and M. Chirwa, “Breastfeeding and mixed feeding practices in Malawi: Timing, reasons, decision makers, and child health consequences.,” *Food & Nutrition Bulletin, 28(1),* pp. 90-99, 2007.

For peer review only

Tables

TABLE 1: Randomisation balance

	Analysis sample		
	Control group	Difference: treatment - control	p value
**Household characteristics**			
Number of members	5.621	0.114	0.875
Number of sleeping rooms	2.036	0.232	0.034**
Household has electricity?	0.002	0.000	0.827
Household has radio?	0.651	0.004	0.897
Household has bicycle?	0.499	0.019	0.699
Household has motorbike?	0.008	-0.001	0.879
Household has paraffin lamp?	0.939	0.017	0.815
Household has oxcart?	0.051	-0.019	0.198
Agricultural	1.000	-0.002	0.422
Main flooring material: dirt, sand or dung	0.924	-0.017	0.565
Main roofing material: natural material	0.876	-0.017	0.697
Piped water	0.015	0.022	0.494
Traditional pit toilet	0.783	0.044	0.356
**Woman characteristics**			
Married	0.718	-0.049	0.046**
Completed primary education	0.709	0.028	0.529
Completed secondary education	0.076	-0.022	0.268
Age	24.592	-0.993	0.026**
Chewa	0.954	-0.039	0.452
Christian	0.983	0.005	0.609
Farmer	0.709	-0.045	0.316
Student	0.164	0.023	0.380
Small business owner	0.040	0.021	0.356
N	411	475	

Notes: Household and mother level characteristics in 2004 corresponding to the sample of children used for analysis. The analysis sample contains children born since July 2005 to married main respondent mothers. The sample is here restricted to one child per household and data is taken from the second follow-up survey only, in which sampled children were aged 0-53 months.

*= p<0.1, **=p<0.05, ***=p<0.01

p values are calculated using the wild cluster bootstrap-t procedure outlined in Cameron et al. (2008).

For peer review only - http://bmjopen.bmj.com/site/about/guidelines.xhtml

<u>TABLE 2:</u> Extended family networks of sampled children.

	Control group	Difference: treatment - control	p value	N
Maternal grandmother (0/1)	0.873	0.012	0.739	2260
Paternal grandmother (0/1)	0.807	0.063	0.132	2252
Mothers sisters	2.835	0.047	0.835	2266
Mothers brothers	2.556	0.207	0.180	2263
Fathers sisters	2.336	0.246	0.290	2266
Fathers brothers	2.453	0.213	0.288	2267

Notes: Mean number of extended family members alive. Sample is all children born since July 2005, who were aged 0-53 months at the time of interview. A pooled dataset from both follow-up surveys is used to construct means.

For peer review only

For peer review only - http://bmjopen.bmj.com/site/about/guidelines.xhtml

Table 3: Relationship between baseline characteristics and family network size

Panel A: Household characteristics

	# members	# rooms	wealth Index
Maternal grandmother alive (0/1)	0.027	-0.04	0.256**
	(0.307)	(0.103)	(0.114)
	{0.929}	{0.779}	{0.032}
Paternal grandmother alive (0/1)	-0.209	-0.02	-0.129
	(0.276)	(0.094)	(0.186)
	{0.478}	{0.919}	{0.549}
Parents siblings alive	0.025	0.01	0.016
	(0.037)	(0.011)	(0.011)
	{0.627}	{0.400}	{0.144}
N	881	879	881

Panel B: Mother characteristics

	Primary education	Secondary education	Age	Chewa	Christian	Farmer	Student	Small business owner
Maternal grandmother alive	0.124*	-0.028	-4.463***	0.01	-0.007	-0.111*	0.150***	-0.046
	(0.057)	(0.033)	(0.687)	(0.039)	(0.010)	(0.054)	(0.033)	(0.029)
	{0.056}	{0.396}	{0.002}	{0.863}	{0.657}	{0.056}	{0.002}	{0.160}
Paternal grandmother alive	0.071**	-0.004	-2.845***	0.005	0.009	-0.01	0.04	-0.024
	(0.032)	(0.029)	(0.563)	(0.023)	(0.012)	(0.036)	(0.024)	(0.027)
	{0.028}	{0.871}	{0.002}	{0.853}	{0.569}	{0.739}	{0.104}	{0.370}
Parents siblings alive	-0.004	0	0.044	0.007	0	0.012**	-0.011***	-0.003*
	(0.004)	(0.002)	(0.069)	(0.005)	(0.001)	(0.004)	(0.003)	(0.001)
	{0.346}	{0.925}	{0.537}	{0.228}	{0.462}	{0.024}	{0.004}	{0.074}
N	881	881	881	881	881	881	881	881

Notes: OLS regressions of with baseline characteristics gathered in 2004 as the dependent variable and family networks as independent variables. The sample contains children born since July 2005 with married, main respondent mothers. Data is from the second follow-up survey only and restricted to one eligible-aged child per household. Standard errors computed using the cluster correlated Huber-White estimator are reported in parentheses and p-values in curly brackets. P values are calculated using the wild cluster bootstrap-t procedure outlined in Cameron et al. (2008).

*= p<0.1, **=p<0.05, ***=p<0.01

For peer review only - http://bmjopen.bmj.com/site/about/guidelines.xhtml

<u>TABLE 4</u>: Height-for-age scores

	(1)	(2)	(3)
Treatment	0.296***	0.441	0.467
	{0.002}	{0.172}	{0.150}
Maternal grandmother		-0.265**	-0.259**
		{0.048}	{0.048}
Maternal grandmother*T		0.168	0.145
		{0.503}	{0.557}
Paternal grandmother		0.008	0.006
		{0.991}	{0.995}
Paternal grandmother*T		-0.253*	-0.235*
		{0.058}	{0.088}
Mothers sisters		0.031	
		{0.394}	
Mothers sisters*T		-0.056	
		{0.296}	
Fathers sisters		0.002	
		{0.995}	
Fathers sisters*T		0.042	
		{0.595}	
Mothers brothers		-0.001	
		{0.949}	
Mothers brothers*T		0.024	
		{0.715}	
Fathers brothers		0.016	
		{0.689}	
Fathers brothers*T		-0.034	
		{0.611}	
Total siblings of mother			0.016
			{0.507}
Total siblings of mother*T			-0.019
			{0.470}
Total siblings of father			0.01
			{0.675}
Total siblings of father*T			0.001
			{0.989}
N	2017	2017	2017

<u>Table notes:</u>

OLS regressions with height for age (HAZ) scores as dependent variable. P-values calculated using the wild cluster bootstrap-t procedure outlined in Cameron et al. (2008) are reported in curly brackets calculated.

All regressions include the following controls:

- Cluster level controls: Education and Chewa ethnicity in 2004
- Household level controls: A wealth index calculated in 2004.
- Mother level controls: Whether she had completed primary school, was working as a farmer or was a student in 2004, current age, age^2 and logarithmic height.
- Child level controls: Month of measurement, age, age^2, gender, number of older siblings, number of older $siblings^2$.

Sample includes all children born after the intervention start date in July 2005 to married main respondent mothers, who were aged 0-53 months at the time of measurement. Column 1 indicates the effect of intervention assignment on HAZ scores, estimated over the sample where family networks information is not missing. Columns 2-6 indicate how intervention effects on HAZ scores vary with the presence of different extended family members.

*= p<0.1, **=p<0.05, ***=p<0.01

For peer review only - http://bmjopen.bmj.com/site/about/guidelines.xhtml

Appendix 1

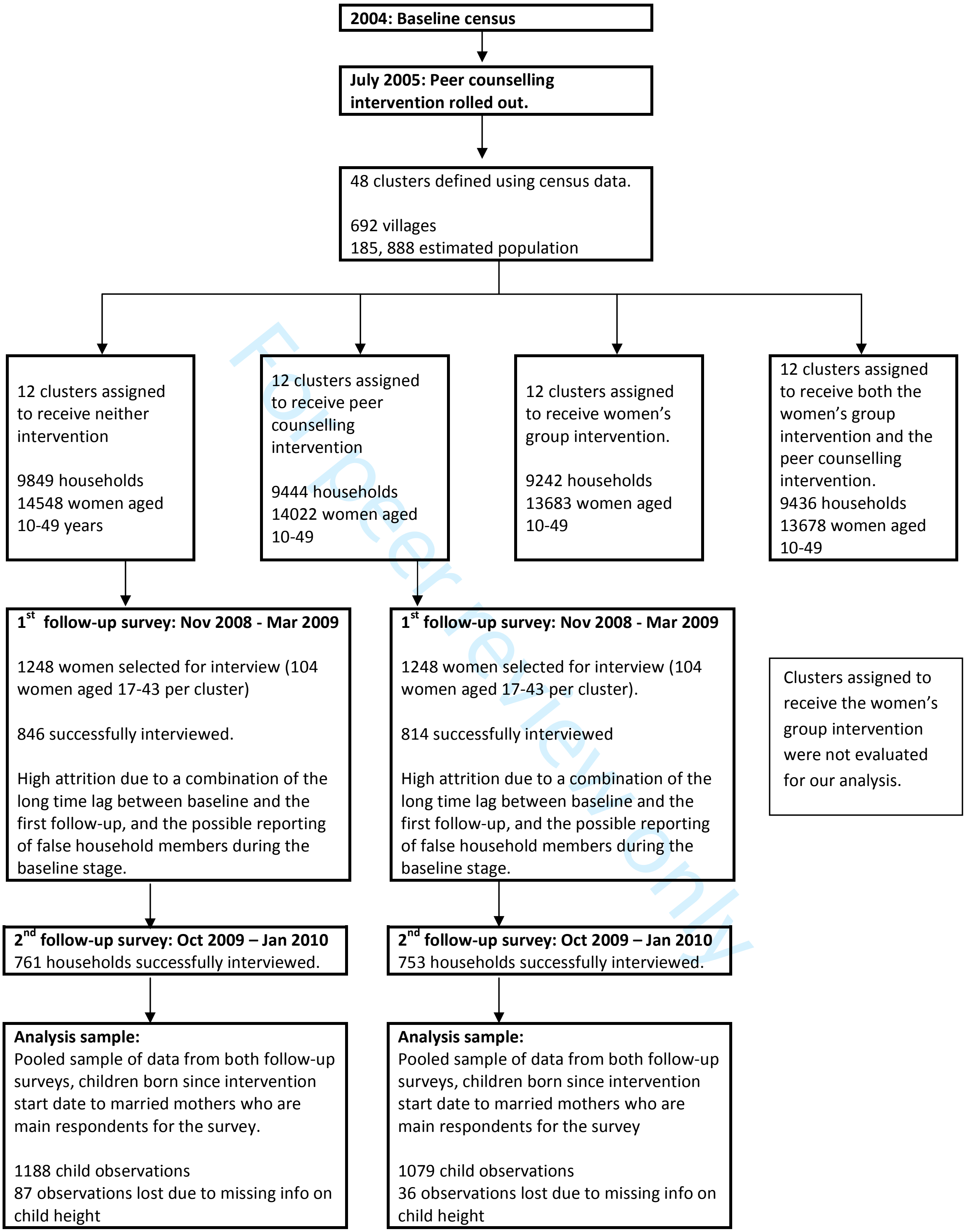

For peer review only

For peer review only - http://bmjopen.bmj.com/site/about/guidelines.xhtml

STROBE Statement—Checklist of items that should be included in reports of ***case-control studies***

	Item No	Recommendation	Complete?
**Title and abstract**	1	(*a*) Indicate the study's design with a commonly used term in the title or the abstract	✓
		(*b*) Provide in the abstract an informative and balanced summary of what was done and what was found	✓ (pg 2)
**Introduction**			
Background/rationale	2	Explain the scientific background and rationale for the investigation being reported	✓ (pg 4)
Objectives	3	State specific objectives, including any prespecified hypotheses	✓ (pg 5)
**Methods**			
Study design	4	Present key elements of study design early in the paper	✓ (pg 5, 7,
Setting	5	Describe the setting, locations, and relevant dates, including periods of recruitment, exposure, follow-up, and data collection	✓ (pg 5 – 7, 10)
Participants	6	(*a*) Give the eligibility criteria, and the sources and methods of case ascertainment and control selection. Give the rationale for the choice of cases and controls	✓ (pg 7-9)
		(*b*) For matched studies, give matching criteria and the number of controls per case	
Variables	7	Clearly define all outcomes, exposures, predictors, potential confounders, and effect modifiers. Give diagnostic criteria, if applicable	✓ (pg 8-9)
Data sources/ measurement	8*	For each variable of interest, give sources of data and details of methods of assessment (measurement). Describe comparability of assessment methods if there is more than one group	✓ (pg 7)
Bias	9	Describe any efforts to address potential sources of bias	✓ (pg 9)
Study size	10	Explain how the study size was arrived at	
Quantitative variables	11	Explain how quantitative variables were handled in the analyses. If applicable, describe which groupings were chosen and why	✓ (pg 8-9)
Statistical methods	12	(*a*) Describe all statistical methods, including those used to control for confounding	✓ (pg 8 -9)
		(*b*) Describe any methods used to examine subgroups and interactions	✓ (pg 8-9)
		(*c*) Explain how missing data were addressed	✓ (pg 9)
		(*d*) If applicable, explain how matching of cases and controls was addressed	
		(*e*) Describe any sensitivity analyses	✓ (pg 8-9)
**Results**			
Participants	13*	(a) Report numbers of individuals at each stage of study—eg numbers potentially eligible, examined for eligibility, confirmed eligible, included in the study, completing follow-up, and analysed	✓ (Appendix 1)
		(b) Give reasons for non-participation at each stage	✓ (Appendix 1)
		(c) Consider use of a flow diagram	✓ (Appendix 1)
Descriptive data	14*	(a) Give characteristics of study participants (eg demographic, clinical,	✓ (Table 1)

For peer review only

For peer review only - http://bmjopen.bmj.com/site/about/guidelines.xhtml

		social) and information on exposures and potential confounders	
		(b) Indicate number of participants with missing data for each variable of interest	✓ (Appendix 1)
Outcome data	15*	Report numbers in each exposure category, or summary measures of exposure	✓ (Table 1)
Main results	16	(*a*) Give unadjusted estimates and, if applicable, confounder-adjusted estimates and their precision (eg, 95% confidence interval). Make clear which confounders were adjusted for and why they were included	✓ (Table 4)
		(*b*) Report category boundaries when continuous variables were categorized	✓
		(*c*) If relevant, consider translating estimates of relative risk into absolute risk for a meaningful time period	
Other analyses	17	Report other analyses done—eg analyses of subgroups and interactions, and sensitivity analyses	✓ (Table 4)
**Discussion**			
Key results	18	Summarise key results with reference to study objectives	✓ (pg 11 - 13)
Limitations	19	Discuss limitations of the study, taking into account sources of potential bias or imprecision. Discuss both direction and magnitude of any potential bias	✓ (pg 16)
Interpretation	20	Give a cautious overall interpretation of results considering objectives, limitations, multiplicity of analyses, results from similar studies, and other relevant evidence	✓ (pg 14 – 17)
Generalisability	21	Discuss the generalisability (external validity) of the study results	✓ (pg 15- 17)
**Other information**			
Funding	22	Give the source of funding and the role of the funders for the present study and, if applicable, for the original study on which the present article is based	✓ (pg 18)

*Give information separately for cases and controls.

Note: An Explanation and Elaboration article discusses each checklist item and gives methodological background and published examples of transparent reporting. The STROBE checklist is best used in conjunction with this article (freely available on the Web sites of PLoS Medicine at http://www.plosmedicine.org/, Annals of Internal Medicine at http://www.annals.org/, and Epidemiology at http://www.epidem.com/). Information on the STROBE Initiative is available at http://www.strobe-statement.org.

For peer review only - http://bmjopen.bmj.com/site/about/guidelines.xhtml

For peer review only

BMJ Open

Family networks and infant health promotion: a mixed-methods evaluation from a cluster randomised controlled trial in rural Malawi

Journal:	*BMJ Open*
Manuscript ID	bmjopen-2017-019380.R1
Article Type:	Research
Date Submitted by the Author:	26-Jan-2018
Complete List of Authors:	Scott, Molly; Oxford Policy Management<br>Malde, Bansi; University of Kent, School of Economics; Insitute for Fiscal Studies, Centre for Evaluation of Development Policies<br>King, C.; UCL, Institute for Global Health<br>Phiri, Tambosi; MaiMwana Project,<br>Chapota, Hilda; MaiMwana Project,<br>Kainja, Esther; MaiMwana Project,<br>Banda, Florida; MaiMwana Project<br>Vera-Hernandez, Marcos; UCL; Insitute for Fiscal Studies, Centre for Evaluation of Development Policies
**Primary Subject Heading**:	Global health
Secondary Subject Heading:	Health policy
Keywords:	Nutrition < TROPICAL MEDICINE, Extended Family, Health promotion, Sub-Saharan Africa, Child Health

SCHOLARONE™ Manuscripts

Family networks and infant health promotion: a mixed-methods evaluation from a cluster randomised controlled trial in rural Malawi

Authors:

Molly Scott[1], Bansi Malde[3,6]*, Carina King[4], Tambosi Phiri[5], Hilda Chapota[5], Esther Kainja[5], Florida Banda[5], Marcos Vera-Hernandez[2,3]

Affiliations:

1. Oxford Policy Management
2. Department of Economics, University College London, UK
3. Centre for Evaluation of Development Policies, Institute for Fiscal Studies, London, UK
4. Institute for Global Health, University College London, UK
5. MaiMwana Project, Mchinji, Malawi
6. School of Economics, University of Kent, UK

***Corresponding author:**

School of Economics, University of Kent, Canterbury, Kent CT2 7NZ, UK. E-mail: b.k.malde@kent.ac.uk; Telephone: (+44)(0)1227816464

Keywords: Extended Family; Child Health; Sub-Saharan Africa; Health Promotion; Child Nutrition

For peer review only - http://bmjopen.bmj.com/site/about/guidelines.xhtml

Abstract:

Objective: Parents may rely on information provided by extended family members when making decisions concerning the health of their children. We evaluate whether extended family members affected the success of an information intervention promoting infant health.

Methods: This is a secondary, sequential mixed-methods study based on a cluster randomised controlled trial of a peer home-education intervention conducted in Mchinji District, Malawi. We used linear multivariate regression to test whether the intervention impact on child height-for-age z-scores (HAZ) was influenced by extended family members. 12 of 24 clusters were assigned to the intervention, in which all pregnant women and new mothers were eligible to receive 5 home visits from a trained peer counsellor to discuss infant care and nutrition. We conducted focus group discussions with mothers, grandmothers and peer counsellors, and key-informant interviews with husbands, chiefs and community health workers to better understand the roles of extended family members in infant feeding.

Results: Exposure to the intervention increased child HAZ scores by 0.296 standard deviations (SD) (95% CI: 0.116, 0.484). However, this effect is smaller in the presence of paternal grandmothers. Compared to an effect size of 0.441 – 0.467 SD (95% CI: -0.344, 1.050) if neither grandmother is alive, the effect size was 0.235 (95% CI: -0.493, 0.039) to 0.253 (95% CI: -0.529, 0.029) SD lower if the paternal grandmother was alive. There was no evidence of an effect of parents' siblings. Maternal grandmothers did not affect intervention impact, but were associated with a lower HAZ score independent of the intervention. Qualitative analysis suggested that grandmothers, who act as secondary caregivers and provide resources for infants, were slower to dismiss traditionally held practices and adopt intervention messages.

Conclusion: The results indicate that the intervention impacts are diminished by paternal grandmothers. Intervention success could be increased by integrating senior women.

Strengths and Limitations:

For peer review only - http://bmjopen.bmj.com/site/about/guidelines.xhtml

- Uses mixed methods to understand how extended family members affected the success of an infant feeding promotion program in Malawi
- Quantitative analysis, using linear multivariate regression, allows estimation of the size of effect different extended family members – including paternal and maternal grandmothers – have on the impact of the intervention
- Focus group discussions and key informant interviews help shed light on the mechanisms through which extended family members might have affected the intervention's success
- The interval between qualitative and quantitative data collection is a limitation, with potential changes to infant feeding practices over time and recall bias.
- Interventions on infant feeding would benefit from understanding and addressing extended family dynamics to improve reach and impact

For peer review only

For peer review only - http://bmjopen.bmj.com/site/about/guidelines.xhtml

Introduction

Child health outcomes are influenced by individuals besides the mother and father, with a rich literature devoted to the contribution of extended family members [1], [2], [3], [4], [5]. In low-income settings, where risk of poor health is high and social welfare nets are minimal, the support of the extended family may be crucial in child-rearing [6]. Relatives can assist by acting as secondary caregivers [7], supplying labour, donating money and providing other in-kind resources to the household [8], [9], [10], [1], [11]. Older women in the family can also affect child outcomes by dispensing infant health and nutrition advice to new mothers [12], [4], [13], [14], [5]. Finally, relatives may exert pressure on mothers, impacting their infant nutrition and health practices [15].

Extended family members may influence the effectiveness of policies and interventions designed to improve child health in low-income settings. On the one hand, they may provide resources and support that complement the intervention making it more effective. On the other hand, they could be resistant to change and reinforce traditional practices, thereby undermining interventions. The latter is particularly relevant in the case of health education and outreach programs, which are widespread across both developed and developing country settings.

Educational programs have the potential to improve child health outcomes by changing widespread misconceptions and traditional behaviours around child feeding and care in low-income settings. In Malawi, for instance, although most infants are breastfed for at least a year, only 40.5% of infants are still exclusively breastfed at 5 months [16]; while diets of children aged over 6 months usually lack sufficient diversity [17], [18]. Beliefs surrounding feeding practices for older infants include the view that the broth of soup is more nourishing than the vegetables or meat inside and that eggs are harmful for children aged 9 months.

For peer review only - http://bmjopen.bmj.com/site/about/guidelines.xhtml

Education campaigns promoting better infant feeding and care to care-givers of infants have shown mixed success in developing country settings. In some cases, they have led to sustained improvements in feeding practices [19], [14] and child physical growth [20], [21], [22]. In others, they have had a negligible effect on child physical growth [23], [24], [25]. Given these mixed findings, there is a need for greater understanding of the factors shaping responses to such interventions across contexts.

Little attention has been paid to the role of extended family members in influencing the success of care-giver focused education interventions in the existing literature, despite their important role in shaping child health. Much of this existing literature is qualitative, with small samples [26], [27]. The small number of quantitative evaluations of education interventions to improve infant feeding that seek to involve extended family members find mixed evidence of effectiveness. Counselling sessions for new adolescent mothers and co-resident grandmothers reduced the un-necessary intake of water and herbal teas within the first 6 months of the child's life in Brazil [28] but failed to maintain breastfeeding of infants at age 2 years [29]; while a behavioural change communication program delivered through older female leaders in Burkina Faso improved infant feeding knowledge but failed to improve child health outcomes [14].

A cluster randomised controlled trial (RCT) conducted in Malawi used a peer home-education strategy to improve rates of exclusive breastfeeding, and reduce infant mortality (see Lewycka et al. 2010, 2013 for further details [30], [31]). The trial achieved a 36% reduction in infant mortality and an improvement in children's height-for-age z-score (HAZ) (increased by 0.271 standard deviations; p=0.022) [17] [31]. This paper uses mixed-methods to investigate whether members of the extended family influenced the success of this peer home visiting intervention, and the possible mechanisms through which their influence might work.

For peer review only - http://bmjopen.bmj.com/site/about/guidelines.xhtml

Methods:

This is a secondary, sequential mixed-methods study based on a cluster RCT of a peer home-education intervention conducted in Mchinji District, Malawi. We investigate family member roles in the success of the intervention, which provided information on healthy infant feeding practices, with quantitative data collected between November 2008 and January 2010, and qualitative data collected in December 2015. Full details of the original trial and methods have previously been published [30] [31]. The quantitative data was collected and analysed first, and used to design the qualitative aspect of the study. The overall interpretation of our findings was integrated following analysis of the qualitative data.

Setting:

Mchinji is a rural district in central Malawi with a population of about 455,000 [32]. Maternal and infant healthcare is delivered at one district hospital, four rural hospitals, nine health centres, private clinics, and in the community through government employed community health workers (CHW – known locally as Health Surveillance Assistants). Much of the healthcare received by pregnant women and infants is in the community setting by CHWs, or at home by kin and other social contacts. However, Malawi has medical pluralism, with traditional practices, beliefs and behaviours such as witchcraft and herbal medicine being commonly used alongside Western medicine. The 2010 Malawi Demographic and Health Survey reported 24% of births in the region occurred in the woman's own home and 2% in someone else's home, and many births are not attended by medically trained healthcare personnel but by traditional birth attendants (14.4%), friends and relatives (8.7%) or no one (2.6%) [16]. Without access to a skilled birth attendant, women are more vulnerable to infection and complications during birth; the infant mortality rate in 2010 was of 66 per 1000 live births [16].

For peer review only - http://bmjopen.bmj.com/site/about/guidelines.xhtml

Traditionally, the main ethnic group in the study area, the Chewa, are a matrilineal and matrilocal group. Matriliny is a system in which land is passed through the female line. Under traditional matrilocal norms, husbands move to their wives' homes after marriage unless they make a special payment. However, following the influence of patrilineal and patrilocal ethnic groups and British colonialists, there is evidence that matrilocality has waned over time, but not completely [33], [34]. As a result, women often remain in close proximity to their own relatives after marriage.

Intervention Description

For the RCT, Mchinji was divided into 48 approximately equal population clusters based on the 1998 Malawi Population and Housing census (the most recent census at the time of trial planning). Within each cluster of around 8000 people, the 3000 individuals living in villages closest to the geographical centre were enumerated as the eligible study population. 12 clusters were assigned to the infant feeding intervention only and 12 served as controls. Full details of the trial set-up and methods are in Lewycka et al., 2010 [30]. All women living in clusters assigned to the infant feeding intervention who became pregnant during the trial period were eligible to receive five home visits from a trained local woman volunteer ('peer counsellor') to discuss maternal and infant healthcare issues; around 60% of eligible women reported having been visited. The visits were timed to coincide with key stages of infant development (the third trimester, and at one week, one month, three and five months after birth). Each visit focused on a specific set of topics for discussion, with special attention paid to nutrition practices including exclusive breastfeeding, and complementary feeding. Peer counsellors were literate local women aged 23-50 years with breastfeeding experience, who each covered a population of about 1000 people.

The intervention began in December 2004, with an initial establishment period until June 2005. The trial was on-going at the time of the quantitative data collection. Following the end of the trial period, peer counsellors continued to receive mentorship and supervision support from government CHWs and the local implementing NGO. In 2015, at the time of qualitative data

For peer review only - http://bmjopen.bmj.com/site/about/guidelines.xhtml

collection, approximately 1/3 of the volunteer counsellors were still active in delivering the intervention.

Quantitative Analysis:

Data and Sample Selection:

A baseline census was conducted in all clusters in 2004, prior to the start of the intervention. All women aged between 10 and 49 were enumerated and a random sample of 104 women aged between 17 and 43 per cluster was then drawn to be interviewed for two follow-up quantitative surveys as part of this secondary study. Sampled women (`main respondent' hereon) were visited to complete the first follow-up in November 2008-March 2009, and a second follow-up in October 2009 –January 2010.

Each follow-up survey contained questions about the size of the extended family of the main respondent and her husband (those alive and those in the village), the health of all household members, food and liquid intake of children aged under 6 years, knowledge about child nutrition, intervention participation (in treatment clusters), and socio-economic variables such as adult work. The height of the main respondent and the height and weight of children under 6 years were also collected by trained enumerators.

The main outcome for our analysis is the child height-for-age z-score (HAZ score), which is a long-term indicator of health that reflects nutrition and morbidity since birth, and should be sensitive to any effects of intervention exposure in early life. It is calculated by comparing the height of the child with the median height in the World Health Organization reference population of children of the same gender and age in months [35].

The sample was balanced between treatment and control clusters along a range of variables collected at baseline (see Fitzsimons et al (2016), Table 1 [17]). The baseline characteristics of the

For peer review only - http://bmjopen.bmj.com/site/about/guidelines.xhtml

two groups remained similar even after accounting for attrition between the baseline and first endline survey, indicating that randomisation was not jeopardised.

For this analysis we use a sample of children who were born since July 2005; and whose mothers are married main respondents in the follow-up surveys (80% of the sample). This sample selection ensures that we measure effects on children whose mothers were eligible to receive visits from a peer counsellor; and allows us to compare effects of the mothers' relatives with those of her husband. Children in the estimation sample were aged between 0 and 53 months at the time of the endline surveys. Appendix 1 provides a timeline of the original trial and the quantitative data collection, and of our sample inclusion criteria.

Table 1 presents the means of basic demographic and socio-economic characteristics for women in the analysis sample living in control clusters at baseline, the differences in the means between the control and treatment groups, and the p-value of this difference. The last two columns allow us to assess whether the randomisation holds in our selected sample. Women assigned to the control group were 24.6 years on average. 71.8% were married and while 70.1% had completed at least primary education, only 7.6% had completed secondary education. In line with the general profile of communities in Mchinji, 95.4% of sampled women were Chewa ethnicity and 98.3% were Christian. The average household size was 5.6 members and all households were engaged in agricultural activity.

Table 2 displays statistics on the size of extended family networks of the children in our analysis sample. Most children have their grandmothers alive (87.3% have maternal grandmothers alive and 80.7% have paternal grandmothers) and their parents have a relatively large number of siblings, with an average of more than two brothers and two sisters each.

<u>Model Specification and Estimation</u>

For peer review only - http://bmjopen.bmj.com/site/about/guidelines.xhtml

The quantitative analysis aims to determine how different family members influence the effectiveness of the infant feeding intervention. Before estimating the main model, we study the relationship between baseline characteristics of mothers and their households and measures of the extended family using linear regression. Table 3 reports these results. It indicates that children whose grandmothers are alive have on average younger mothers, who are more likely to have completed at least primary education, less likely to be working as farmers in 2004, and are from more socioeconomically advantaged households, as measured by a composite wealth index constructed using principal components analysis as recommended by [36].

Our main specification is the following linear multivariate regression:

$$HAZ_{ij} = \alpha + \beta T_j + \beta_2 Maternal\_grandmother_{ij} + \beta_3 Maternal\_grandmother_{ij}{}^{*}T_j$$
$$+ \beta_4 Paternal\_grandmother_{ij} + \beta_5 Paternal\_grandmother_{ij}{}^{*}T_j$$
$$+ \beta_6 Total\_mothers\_siblings_{ij} + \beta_7 Total\_mothers\_siblings_{ij}{}^{*}T_j$$
$$+ \beta_8 Total\_fathers\_siblings_{ij} + \beta_9 Total\_fathers\_siblings_{ij}{}^{*}T_j + X_{ij}\gamma$$
$$+ Z_j\gamma_2 + t + \epsilon_{ij}$$

where HAZ_{ij} is the height-for-age z-score of child i in cluster j. T_j is a treatment exposure indicator which captures whether the child was born to a mother living in 2004 (pre-intervention) in a cluster that was assigned to receive the program. We therefore use an intent-to-treat estimator. $Maternal\_grandmother_{ij}$ and $Paternal\_grandmother_{ij}$ are binary variables indicating, respectively, whether the maternal and paternal grandmother is alive. $Total\_mothers\_siblings_{ij}$ ($Total\_fathers\_siblings_{ij}$) captures the total number of siblings of the child's mother (father) who are alive. We use two definitions of this variable in different specifications of the model: (i) Brothers and sisters (separately) of each parent, and (ii) the total siblings of each parent. X_{ij} and Z_j are vectors of control variables at the individual and cluster level respectively. These include all baseline characteristics where significant differences between households with different

For peer review only - http://bmjopen.bmj.com/site/about/guidelines.xhtml

extended family members alive were detected, and interview month and year indicators to account for month-year-specific shocks. We do not adjust the data for missing information.

We fitted three models, one crude model with the intervention term only, and two full models as specified in the equation each treating parent siblings differently.

The coefficient β captures the effect of the program for children whose maternal and paternal grandmothers are dead, and whose parents are only children. The coefficients β_3, β_5, β_7 and β_9, associated with interaction terms between variables capturing extended family relations and the indicator for program allocation, estimate the additional effect of the program for children with different types and numbers of extended family members. A positive (or negative) significant interaction provides evidence that the intervention effect is enhanced (or diminished) in the presence of that particular family member. Through the coefficients β_2, β_4, β_6 and β_8, the specification also accounts for the possibility that, independently of the intervention, the HAZ score might be different depending on what family members are alive.

Errors ε_{ij} are assumed to be uncorrelated between individuals in different clusters but are allowed an unrestricted correlation structure within clusters. To account for correlation within clusters, standard errors must be adjusted to prevent downward bias, and incorrect inference. Given the small number of clusters in the study (12 intervention and 12 control clusters), we adopt wild cluster bootstrap methods as recommended in Cameron et al. (2008) [37]. Associated 95% confidence intervals can be calculated using a computationally intensive method suggested in [38]. The bootstrap adjustment applied here was studied in detail by Fitzsimons et al (2016) and was found to perform well [17]. Data from both follow-up surveys is pooled to improve statistical power.

The extended family network is defined according to which members of the family are alive, rather than which ones live in the same village or household. This is in case treatment exposure

For peer review only

affected decisions over where to live, which would cause a measure of family network size based on residence to be correlated with the intervention, and thereby bias estimates. The benefit of defining the size of the family network according to which members are alive is that this is almost certain to be invariant to program exposure.

We choose to define T_j by exposure to the intervention rather than actual participation since participation in the program was voluntary and also relied on the ability of peer counsellors to locate eligible women. Women who peer counsellors did not manage to trace or who chose not to take part in the program may be different from those who did participate. The existence of such systematic differences would potentially introduce some unobserved correlation between the treatment interaction variables and HAZ scores if T_j were defined on the basis of actual participation. Indeed, Fitzsimons et al. (2016) report that women who received the visits tend to be poorer [17]. Defining treatment based on residence at baseline rather than at the time of the follow-up interviews also alleviates concerns of bias in case there was purposeful migration into treated areas by control-group assigned households.

Qualitative Analysis

Following the findings from the quantitative analysis, we conducted focus group discussions (FGDs) with grandmothers, mothers and peer counsellors, and semi-structured interviews with fathers, CHWs and village chiefs to gain a more in-depth understanding of family roles and how grandmothers might influence child health.

Recruitment

Participants were recruited from 11 of 24 intervention and control clusters across the district in late 2015. Mothers, grandmothers and fathers were purposively selected by CHWs and chiefs to represent those households who had actively received the intervention or had children under-5 years in control clusters. Volunteer peer counsellors were contacted directly, to represent a range

For peer review only - http://bmjopen.bmj.com/site/about/guidelines.xhtml

of ages and years' experience as counsellors. Chiefs and CHWs were purposively selected and contacted directly to represent clusters with a range of engagement with the intervention. We planned to conduct a total of five FGDs and 10 interviews, rather than collecting data until saturation was reached.

Data Collection:

FGDs and interviews used topic guides, based on the quantitative findings and wider literature on infant feeding behaviours, covering: household decision making around feeding, infant feeding practices, feeding knowledge and sources of information about infant feeding. We asked about all household members, and specifically probed about the role of grandmothers. All discussions were facilitated by two local trained qualitative researchers in Chichewa. Participants were reimbursed for their travel expenses and given refreshments. All discussions were audio recorded, and then verbatim transcribed in Chichewa. Transcripts were translated into English as a group, with ambiguous terms or phrases debated until a consensus meaning was reached. Data collection, transcription and translation were conducted by EK, HC, TP and FM – female Malawian researchers who are fluent in English.

Analysis

The English transcripts were coded using an inductive framework approach based on the following steps: familiarisation, coding, developing and applying the framework, charting and interpretation [39]. All transcripts were double-coded, as a group by TP, EK, HC and FB and independently by CK – a female British researcher with five years' work experience in Malawi. Coding was done on paper and the coding matrix developed in Microsoft Excel. A round-table discussion was then conducted by all five researchers to compare the codes and agree on themes; disagreements in coding were discussed until an agreement on the interpretation was reached.

Ethics

For peer review only - http://bmjopen.bmj.com/site/about/guidelines.xhtml

All participants gave informed written consent. Ethical approval to conduct this study was obtained from the National Health Sciences Research Committee in Malawi [Protocol Numbers: 491; 15/9/1483].

Results

Quantitative Analysis:

Table 4 displays the results of the quantitative analysis on child HAZ scores. Model 1 shows that overall exposure to the program raised HAZ scores by 0.296 (95% CI: 0.116, 0.484) standard deviations (SD). Models 2 and 3 present the results for the regressions that test whether different family members influenced the effectiveness of the intervention on child HAZ scores. Model 2 presents the results where we allow for brothers and sisters of the child's parents to have different effects, while Model 3 displays those including (separately) the total siblings of each of the child's parents.

For children whose parents have no living mothers or siblings, the effect of the intervention on HAZ scores is between 0.441 (95% CI: -0.335, 1.028) and 0.467 (95% CI: -0.344, 1.050) SD. However, for those children with a living paternal grandmother, the intervention effect was reduced by between 0.235 (95% CI: -0.493, 0.039) and 0.253 (95% CI: -0.529, 0.029) SD. The results also suggest that, independently of whether they are treated by the intervention or not, children whose maternal grandmothers are alive have lower HAZ scores. Finally, the results uncover no association between the number of parents' siblings on HAZ scores, or of differential effects of the intervention by these.

Qualitative Analysis:

We conducted 5 FGDs, with 37 participants of 48 invited (mothers=16; grandmothers=15; peer counsellors=6), and 10 semi-structured interviews (village chiefs=4, fathers=4; CHW=2). We defined the following emergent themes in relation to grandmothers and their role in infant

For peer review only - http://bmjopen.bmj.com/site/about/guidelines.xhtml

feeding and growth: decision-making roles, knowledge and information, traditional practices and intervention successes and challenges around behaviour change.

Decision-Making Roles: Across the respondents there was agreement that the father is responsible for resource allocation and mobilisation, while the mother's role is to manage and prepare food for the household. When there is a lack of food or resources, extended family members or neighbours can provide assistance, for example "*maybe you have found you don't even have flour, our sister in law or mother in law gives it to you saying that it's only for the child, prepare porridge so that it should eat"* (Mother 5, Control). Within the household, grandmothers, both maternal and paternal, were generally viewed as the secondary caregivers, providing support by cooking for and feeding infants.

Information and Knowledge: Sources of information about infant feeding included: antenatal clinics and other healthcare, family members, village chiefs and community meetings, the peer counsellor intervention and other NGOs and civil society education programmes. Interestingly, the peer counsellors were reported as a source of information by participants from control areas, likely reflecting contamination following the end of the trial period. Although grandmothers report giving similar advice as that given by healthcare workers:

"*now we are afraid, so we provide the same advice they give at the clinic, so we tell them the same things*" (Grandmother 7, Intervention),

However, reports from peer counselors cast doubt on this. They instead mentioned encountering difficulties with grandmothers when disseminating their advice: "*frequently the grandmothers mislead, mislead them as they say what they were doing before in their time*" (Peer Counselor 5).

Peer counselors, however, also noticed a change over time in attitudes among grandmothers, with increased acceptance of the intervention messages: "*the group of relatives which gives the most problems is the grandparents because they tell the woman that 'aaah [the counselors] are just*

For peer review only - http://bmjopen.bmj.com/site/about/guidelines.xhtml

cheating you, they want this child to be crying' [...] but we have seen that the grandmothers now have understood" (Peer Counselor 4).

Despite extended family members not being the target group of the intervention, breastfeeding, weaning and complementary feeding messages appear to have disseminated, with fathers ("*advice about breast feeding, I know a lot; when a child is born he should breast feed exclusively, very frequently*" – Father 1, Intervention) and grandmothers ("*so they say breastfeed frequently these days, that's the modern way of childbirth, so you also say breastfeed the child*" – Grandmother 2, Intervention) demonstrating accurate knowledge.

Traditional Practices: Several different traditional practices and beliefs about infant feeding were mentioned by all respondent types, including: adding medicinal herbs to infants' porridge; believing children become "foolish" is breastfed for too long; and smaller portions making children "smart". However, mothers and grandmothers in both intervention and control areas commented that while these practices and beliefs are known to exist, they are no longer commonplace ("*most of this generation do not follow [these practices]*" – Mother 8, Control) or rituals and the giving of herbal medicines is done in hiding. This was confirmed by one of the CHW who commented that: "*while the grandmothers and the other people have their own beliefs, our role is to get rid of those beliefs […] little by little people change*" (CHW 2, Intervention).

Behaviour Change: Community members reported sustained behaviour change relating to exclusive breastfeeding and facility-based deliveries: "*behaviour these days has changed in that delivering at home is no longer there [...] we say go to the hospital*" (Grandmother 5, Intervention). However, CHW and peer counselors noted that these changes were not seen immediately, and that barriers such as lack of engagement, lack of understanding and cultural issues (e.g. urban women 'looking down' on the counselors) were present.

Discussion:

For peer review only - http://bmjopen.bmj.com/site/about/guidelines.xhtml

Our mixed-methods evaluation of the effect of extended family members on the impact of a peer-led home education intervention in rural Malawi suggests that living grandmothers can be a barrier to intervention dissemination and behaviour change. The qualitative findings complement the quantitative results, and suggest the mechanism through which grandmothers may influence the effectiveness of the peer intervention.

The apparently negative influence of paternal grandmothers on intervention success may be due to a conflict between their views on infant feeding from the recommendations of peer counsellors. The qualitative findings offer some support for this hypothesis by providing evidence that grandmothers are proponents of 'traditional' views of child feeding that differ from standard recommendations, supported by previous studies [4], [12]. They indicate that grandmothers persist in their traditional beliefs for longer, and as providers of both financial and childcare support, exert influence towards their own beliefs of child feeding rather than towards the information provided by the intervention. Reassuringly though, our data suggest that grandmothers eventually adjust their practices to be in line with the information provided by the intervention.

Interestingly, the qualitative data did not distinguish between paternal and maternal grandmothers, despite other evidence that, at least in Malawi, it is paternal grandmothers who command the most influence [12]. This may explain why we do not find a similar negative effect on intervention success associated with maternal grandmothers in the quantitative estimation. However, our ability to speculate on different mechanisms of action between maternal and paternal grandmothers is limited.

The delays seen in attitude change amongst grandmothers suggest that there may be potential to increase the intervention's impact further by engaging extended family members in the information exchange process. A growing body of evidence underscores the benefits of more inclusive approaches to health education [40], [12], [13], [14], and cautions against assuming that

For peer review only - http://bmjopen.bmj.com/site/about/guidelines.xhtml

new information will necessarily be incorporated into knowledge and behaviour. Actual response will, in general, depend on the mode of transmission. Approaches that treat users of information as passive are less likely to be effective than those that foster dialogue within the target communities.

In contexts where older women exert particular influence, there are clear grounds for designing interventions that respect and acknowledge their seniority. The rationale behind the work of organisations like the Grandmother Project is that elder women can act as powerful agents for change if they are mobilised and empowered to support intervention aims [4], [12]. We consider our findings to provide support for this agenda. However, it must be noted that involving senior women in interventions might not be sufficient to improve child health, particularly in contexts where poor nutrition is not the only cause for poor health. Evidence from the evaluation of an integrated agriculture and nutrition and health behaviour change communication programme indicates that senior women can be effective in changing knowledge, but this improved knowledge might still fail to yield improvements in child growth [14].

We uncover a negative association of maternal grandmothers in the absence of the intervention. However, this cannot be taken as evidence of a causal effect, because of the presence of confounders such as a higher competition for resources in families with living maternal grandmothers in matrilineal societies [41].

The qualitative findings also raise the importance of the role of men as key providers and resource mobilisers. Previous quantitative evidence Fitzsimons et al (2016) [17] supports the critical role of males in ensuring adoption of the information provided. Therefore, integrating these influential figures with the peer counsellor intervention may help improve uptake and reduce the time to intervention acceptance we currently observe.

For peer review only - http://bmjopen.bmj.com/site/about/guidelines.xhtml

In this study there were several limitations. Firstly, it is impossible to ascertain whether the estimated quantitative detrimental effect of grandmothers is due to their presence and not because households in which the paternal grandmother is alive are different in some characteristic that is omitted from the regression and that affects the effectiveness of the intervention. The survey and qualitative data may be subject to social desirability bias, with respondents providing answers which they think will please the researchers. As the qualitative and quantitative data triangulated, and respondents were not aware of our hypothesis, we do not feel this considerably biased our conclusions. Finally, the mixed data were collected sequentially rather than concurrently, with the qualitative data collection conducted five years after the quantitative survey. This may have resulted in recall bias in the qualitative data, and the culture and behaviours around infant feeding may have shifted between the two study phases. This is somewhat supported by the FGDs and interviews from control areas being exposed to the peer counsellors and their messages, posing a challenge to integrating the results. However, as the qualitative data was planned to provide a more in-depth understanding and triangulation of the quantitative findings, rather than comment on causality, we do not feel this detracts from our interpretation.

We found that grandmothers play an important role in shaping responses to an information campaign targeting infant health. In order to increase the impact of information campaigns, our findings suggest that excluding influential older women, who act as both important sources of advice and childcare support, can weaken intervention impact by exposing a divergence between traditional views and new information. Inclusive health education approaches that respect the need to tackle existing traditional beliefs and the roles that grandmothers play, may overcome this friction and improve the effectiveness of the intervention.

For peer review only - http://bmjopen.bmj.com/site/about/guidelines.xhtml

Acknowledgements: We thank Andrew Mganga, Nicholas Mbwana, Christopher Kamphinga, Sonia Lewycka, Mikey Rosato and Julia Behrman for advice and assistance with quantitative data collection. We also thank Ravi Somani for his assistance in coding the program to obtain the confidence intervals.

Funding: Funding from the ESRC/DFID grant ES/J009253/1 is gratefully acknowledged.

Conflict of interest: We declare that we have no conflicts of interest.

Authors contributions: All authors provided final approval of the version to be published. Concept and design of quantitative analysis: MS, BM and MV; analysis and interpretation of quantitative findings: MS, MV and BM; drafting the article and revising it critically: MS, BM, CK and MV. Concept and design of the qualitative study: BM, MVH, CK and TP. Qualitative data collection and analysis: TP, HC, EK, FB. Qualitative analysis was independently checked and coded: CK. CK, TP, EK, HC and FB discussed and agreed the final interpretation.

Data sharing: The raw quantitative data is available for download from the UK Data Archive.

For peer review only

References

[1] D. LaFave and D. Thomas, “Extended Families and Child Well-being,” *Journal of Development Economics,* vol. 126, no. C, pp. 52-65, 2017.

[2] P. Sheppard and R. Sear, “Do grandparents compete with or support their grandchildren? In Guatemala, paternal grandmothers may compete, and maternal grandmothers may cooperate,” *Royal Society Open Science,* vol. 3, no. 160069, 2016.

[3] B. Strassman and W. Garrard, “Alternatives to the grandmother hypothesis: A meta-anaalysis of the association between grandparental and grandchild survival in patrilineal populations,” *Human Nature,* vol. 22, no. 1, p. 201, 2011.

[4] J. Aubel, I. Touré and M. Diagne, “Senegalese grandmothers promote improved maternal and child nutrition practices: the guardians of traditional are not averse to change.,” *Social Science & Medicine, 59(5),* pp. 945-959, 2004.

[5] M. Gupta, R. Aborigo, P. Adongo, S. Rominski, A. Hogdson, C. Engmann and C. Moyer, “Grandmothers as gatekeepers? The role of grandmothers in influencing health-seeking for mothers and newborns in rural Ghana,” *Global Public Health,* vol. 10, no. 9, pp. 1078-1091, 2015.

[6] S. B. Hrdy, Mother nature: A history of mothers, infants, and natural selection., New York: Pantheon Books, 1999.

[7] P. K. Ivey, “Cooperative Reproduction in Ituri Forest Hunter-Gatherers: Who Cares for Efe Infants? 1.,” *Current Anthropology, 41.5,* pp. 856-866, 2000.

[8] M. A. Gibson and R. Mace, “Helpful Grandmothers in rural Ethiopia: A study of the effect of kin on child survival and growth,” *Evolution and Human Behaviour 26(6),* pp. 469-482, 2005.

[9] R. Lee and K. Kramer, “Children's Economic Roles in the Maya Family Life Cycle: Cain, Caldwell, & Chayanov Revisited,” *Population and Development Review, Vol. 28, No. 3,* pp. 475-499, 2002.

[10] M. Angelucci, G. De Giorgi, M. Rangel and I. Rasul, “Family networks and school enrolment: Evidence from a randomised social experiment.,” *Journal of Public Economics, 94(3),* pp. 197-221, 2010.

[11] E. Duflo, “Grandmothers and Granddaughters: Old-Age Pensions and Intrahousehold Allocation in South Africa,” *World Bank Economic Review,* vol. 17, no. 1, pp. 1-25, 2003.

[12] R. Bezner-Kerr, L. Dakishoni, L. Shumba, R. Msachi and M. Chirwa, “"We grandmothers know plenty": breastfeeding, complementary feeding and the multifaceted role of grandmothers in Malawi.,” *Social Science & Medicine, 66(5),* pp. 1095-1105, 2008.

For peer review only - http://bmjopen.bmj.com/site/about/guidelines.xhtml

[13] J. Aubel and D. Sihalathavong, "Participatory communication to strengthen to role of grandmothers in child health: an alternative paradigm for health education and health communication.," *Journal of International Communication, 7(2),* pp. 76-97, 2001.

[14] D. K. Olney, A. Pedehombga, M. T. Ruel and A. Dillon, "A 2-Year Integrated Agriculture and Nutrition and Health Behavior Change Communication Program Targeted to Women in Burkina Faso Reduces Anemia, Wasting and Diarrhea in Children 3-12.9 Months of Age at Baseline: A Cluster-Randomized Controlled Trial," *The Journal of Nutrition,* vol. 145, no. 6, pp. 1317 - 1324, 2015.

[15] O. Agunbiade and O. Ogunleye, "Constraints to exclusive breastfeeding practice among breastfeeding mothers in Southwest Nigeria: implications for scaling up," *International Breastfeeding Journal,* vol. 7, no. 1, p. 5, 2012.

[16] National Statistical Office, Malawi, Macro ORC, "Malawi Demographic and Health Survey," Zomba: Malawi National Statistical Office, Macro ORC, 2004.

[17] E. Fitzsimons, B. Malde, A. Mesnard and M. Vera-Hernandez, "Nutrition, Information, and Household Behaviour: Experimental Evidence from Malawi," *Journal of Development Economics,* vol. 122, pp. 113-126, 2016.

[18] B. F. Kalanda, F. H. Verhoeff and B. J. Brabin, "Breast and complementary feeding practices in relation to morbidity and growth in Malawian infants.," *European journal of clinical nutrition, 60(3),* pp. 401-407, 2006.

[19] A. L. Morrow, M. L. Guerrero, J. Shults, J. Calva, C. Lutter, J. Bravo, G. Ruiz-Palacios, R. Morrow and F. Butterfoss, "Efficacy of home-based peer counselling to promote exclusive breastfeeding: a randomised controlled trial.," *The Lancet, 353(9160),,* pp. 1226-1231, 1999.

[20] G. Guldan, H. M. X. Fan, Z. Ni, X. Xiang and M. Tang, "Culturally appropriate nutrition education improves infant feeding and growth in rural Sichuan, China," *Journal of Nutrition,* vol. 130, no. 5, pp. 1204-11, 2000.

[21] N. Bhandari, S. Mazumder, R. Bahl, J. Martines, R. Black, M. Bhan and I. F. S. Group, "An educational intervention to promote appropriate complementary feeding practices and physical growth in infants and young children in rural Haryana, India," *Journal of Nutrition,* vol. 134, no. 9, pp. 2342-8, 2004.

[22] M. Penny, H. Creed-Kanashiro, R. Robert, M. Narro, L. Caulfield and R. Black, "Effectiveness of an educational intervention delivered through the health services to improve nutrition in young children: a cluster-randomised controlled trial," *The Lancet,* vol. 365, no. 9474, pp. 1863-72, 2005.

[23] I. Engebretsen, D. Jackson, L. Fadnes, V. Nankabirwa, A. Diallo, T. Doherty, C. Lombard, S. Swanvelder, J. Nankunda, V. Ramokolo, D. Sanders, H. Wamani, N. Meda, J. Tumwine, E. Ekstrom, P. Van de Perre, C. Kankasa, H. Sommerfelt and T. Tylleksar, "Growth effects of

For peer review only - http://bmjopen.bmj.com/site/about/guidelines.xhtml

exclusive breastfeeding promotion by peer counsellors in sub-Saharan Africa: the cluster-randomised PROMISE EBF trial," *BMC Public Health,* vol. 14, no. 1, p. 633, 2014.

[24] N. Bhandari, R. Bahl, B. Nayyar, P. Khokhar, J. Rohde and M. Bhan, "Food supplementation with encouragement to feed it to infants from 4 to 12 months of age has a small impact on weight gain," *Journal of Nutrition,* vol. 131, no. 7, pp. 1946-51, 2001.

[25] A. Guyon, V. Quinn, Z. Rambeloson and M. Hainsworth, "Using the Essential Nutrition Actions Approach to Improve the Nutritional Practices of Women and Children at Scale in Antananarivo and Fianarantsoa Provinces of Madagascar: Results and Trends 2000 to 2005," USAID, 2006.

[26] A. Mukuria, S. Martin, T. Egondi, A. Bingham and F. Thuita, "Role of social support in improving infant feeding practices in Western Kenya: A Quasi-Experimental Study," *Global Health, Science and Practice,* vol. 4, no. 1, pp. 55-72, 2016.

[27] A. DeLorme, E. Gavenus, C. Salmen, G. Benard, B. Mattah, E. Bukusi and K. Fiorella, "Nourishing networks: A socio-ecological analysis of a network intervention for improving household nutrition in Western Kenya," *Social Science and Medicine,* vol. 197, pp. 95-103, 2018.

[28] L. Nunes, E. Giugliani, L. Santo and L. de Oliveira, "Reduction of unnecessary intake of water and herbal teas on breast-fed infants: a randomized clinical trial with adolescent mothers and grandmothers," *Journal of adolescent health,* vol. 49, no. 3, pp. 258-64, 2011.

[29] C. da Silva, L. Nunes, R. Schwartz and E. Giugliani, "Effect of a pro-breastfeeding intervention on the maintenance of breastfeeding for 2 years or more: randomized clinical trial with adolescent mothers and grandmothers," *BMC Pregnancy and Childbirth,* vol. 16, no. 3, p. 97, 2016.

[30] S. Lewycka, C. Mwansambo, P. Kazembe, T. Phiri, A. Mganga, M. Rosato, H. Chapota, F. Malamba, S. Vergnano, M. Newell, D. Orsin and A. Costello, "A cluster randomised controlled trial of the community effectiveness of two interventions in rural Malawi to improve health care and to reduce maternal, newborn and infant mortality," *Trials, 11(1), 88,* 2010.

[31] S. Lewycka, C. Mwansambo, M. Rosato, P. Kazembe, T. Phiri, A. Mganga, H. Chapota, F. Malamba, E. Kainja, M. Newell, G. Greco, A. Pulkki-Brännström, J. Skordis-Worrall, S. Vergnano, D. Osrin and A. Costello, "Effect of women's groups and volunteer peer counselling on rates of mortality, morbidity, and health behaviours in mothers and children in rural Malawi (MaiMwana): a factorial, cluster-randomised controlled trial.," *The Lancet, 381(9879),* pp. 1721-1735, 2013.

[32] National Statistical Office, Malawi, "National Population and Housing Census: Analytical Report," 1998.

[33] G. Reniers and R. Tfaily, "Polygyny and HIV in Malawi.," *Demographic Research, 19(53),* p. 1811, 2008.

[34] T. Takane, "Tobacco and smallholders in Malawi: village case studies in the Mchinji and

For peer review only - http://bmjopen.bmj.com/site/about/guidelines.xhtml

Mangochi Districts.," *African Research Series, 93-119.,* 2005.

[35] WHO Anthro for personal computers, version 3.2.2, *Software for assessing growth and development of the world's children.,* Geneva: WHO, 2011.

[36] D. Filmer and L. Pritchett, "Estimating wealth effects without expenditure data - or tears: An application to educational enrollments in states of India," *Demography,* vol. 38, no. 1, pp. 115-132, 2001.

[37] A. C. Cameron, J. B. Gelbach and D. L. Miller, "Bootstrap-based improvements for inference with clustered errors.," *The Review of Economics and Statistics, 90(3),* pp. 414-427, 2008.

[38] A. Cameron and D. Miller, "A Practitioner's Guide to Cluster-Robust Inference," *Journal of Human Resources,* vol. 50, no. 2, pp. 317-372, 2015.

[39] N. K. Gale, G. Heath, E. Cameron, S. Rashid and S. Redwood, "Using the Framework Method for the Analysis of Qualitative Data in Multi-Disciplinary Health Research," *BMC Medical Research Methodology,* vol. 13:117, 2013.

[40] R. G. Lee and T. Garvin, "Moving from information transfer to information exchange in health and health care.," *Social science & medicine, 56(3),* pp. 449-464, 2003.

[41] R. Sear, "Kin and child survival in rural Malawi," *Human Nature, 19(3),* pp. 277-293, 2008.

For peer review only

Tables

TABLE 1: Distribution of household and women characteristics in controls and differences with treatment group

	Analysis sample		
	Control group (Mean or proportion)	Difference: treatment - control	p value
**Household characteristics**			
Number of members[a]	5.621	0.114	0.875
Number of sleeping rooms[a]	2.036	0.232	0.034**
Household has electricity?[b]	0.2%	0.0%	0.827
Household has radio?[b]	65.1%	0.4%	0.897
Household has bicycle?[b]	49.9%	1.9%	0.699
Household has motorbike?[b]	0.8%	-0.1%	0.879
Household has paraffin lamp?[b]	93.9%	1.7%	0.815
Household has oxcart?[b]	5.1%	-1.9%	0.198
Agricultural household[b]	100%	-0.2%	0.422
Main flooring material: dirt, sand or dung[b]	92.4%	-1.7%	0.565
Main roofing material: natural material[b]	87.6%	-1.7%	0.697
Piped water[b]	1.5%	2.2%	0.494
Traditional pit toilet[b]	78.3%	4.4%	0.356
Wealth index[a]	-0.087	0.034	0.897
**Woman characteristics**			
Married[b]	71.8%	-4.9%	0.046**
Completed primary education[b]	70.9%	2.8%	0.529
Completed secondary education[b]	7.6%	-2.2%	0.268
Age[a]	24.592	-0.993	0.026**
Chewa[b]	95.4%	-3.9%	0.452
Christian[b]	98.3%	0.5%	0.609
Farmer[b]	70.9%	-4.5%	0.316
Student[b]	16.4%	2.3%	0.380
Small business owner[b]	4.0%	2.1%	0.356
N	411	475	

Notes: Household and mother level characteristics in 2004 corresponding to married main respondent mothers present in the second follow-up survey with children born after the intervention began in July 2005. [a] Continuous variable who which the mean is reported; [b] Binary variable, for which proportions are reported.

*= p<0.1, **=p<0.05, ***=p<0.01. p values are calculated using the wild cluster bootstrap-t procedure outlined in Cameron et al. (2008).

For peer review only - http://bmjopen.bmj.com/site/about/guidelines.xhtml

<u>TABLE 2:</u> Distribution of family networks indicators in controls and differences with treatment group for sampled children.

	Control group	Difference: treatment - control	p value	N
Maternal grandmother[a]	87.3%	1.2%	0.739	2260
Paternal grandmother[a]	80.7%	6.3%	0.132	2252
Mother's sisters[b]	2.835	0.047	0.835	2266
Mother's brothers[b]	2.556	0.207	0.180	2263
Father's sisters[b]	2.336	0.246	0.290	2266
Father's brothers[b]	2.453	0.213	0.288	2267

Notes: [a]Binary variable for which percentages are reported. [b]Discrete, non-binary variables for which mean values are reported. Sample includes all children born since July 2005, who were aged 0-53 months at the time of interview, and whose mothers were married main respondents to the follow up surveys in 2008-09 and 2009-10. A pooled dataset from both follow-up surveys is used to construct means.

For peer review only - http://bmjopen.bmj.com/site/about/guidelines.xhtml

For peer review only

Table 3: Relationship between baseline characteristics and family network size, with p-values

Panel A: Household characteristics

	Number of members	Number of rooms	wealth Index
Maternal grandmother alive (0/1)	0.027	-0.04	0.256**
SE	(0.307)	(0.103)	(0.114)
Cluster wild bootstrap t p-value	{0.929}	{0.779}	{0.032}
Paternal grandmother alive (0/1)	-0.209	-0.02	-0.129
SE	(0.276)	(0.094)	(0.186)
Cluster wild bootstrap t p-value	{0.478}	{0.919}	{0.549}
Parents siblings alive	0.025	0.01	0.016
SE	(0.037)	(0.011)	(0.011)
Cluster wild bootstrap t p-value	{0.627}	{0.400}	{0.144}
N	881	879	881

Panel B: Mother characteristics

	Primary education	Secondary education	Age	Chewa	Christian	Farmer	Student	Small business owner
Maternal grandmother alive	0.124*	-0.028	-4.463***	0.01	-0.007	-0.111*	0.150***	-0.046
SE	(0.057)	(0.033)	(0.687)	(0.039)	(0.010)	(0.054)	(0.033)	(0.029)
Cluster wild bootstrap t p-value	{0.056}	{0.396}	{0.002}	{0.863}	{0.657}	{0.056}	{0.002}	{0.160}
Paternal grandmother alive	0.071**	-0.004	-2.845***	0.005	0.009	-0.01	0.04	-0.024
SE	(0.032)	(0.029)	(0.563)	(0.023)	(0.012)	(0.036)	(0.024)	(0.027)
Cluster wild bootstrap t p-value	{0.028}	{0.871}	{0.002}	{0.853}	{0.569}	{0.739}	{0.104}	{0.370}
Parents siblings alive	-0.004	0	0.044	0.007	0	0.012**	-0.011***	-0.003*
SE	(0.004)	(0.002)	(0.069)	(0.005)	(0.001)	(0.004)	(0.003)	(0.001)
Cluster wild bootstrap t p-value	{0.346}	{0.925}	{0.537}	{0.228}	{0.462}	{0.024}	{0.004}	{0.074}
N	881	881	881	881	881	881	881	881

Notes: OLS regressions of with baseline characteristics gathered in 2004 as the dependent variable and family networks as independent variables. Sample contains married main respondent mothers present in the second follow-up survey with children born after the intervention began in July 2005.. Standard errors computed using the cluster correlated Huber-White estimator are reported in parentheses and p-values in curly brackets. P values are calculated using the wild cluster bootstrap-t procedure outlined in Cameron et al. (2008). The wealth index was calculated using principal components analysis as recommended by [36].
*= p<0.1, **=p<0.05, ***=p<0.01

For peer review only - http://bmjopen.bmj.com/site/about/guidelines.xhtml

TABLE 4: Estimated effects on height-for-age z-scores with 95% confidence intervals from three linear regression models

	Model 1		Model 2		Model 3	
	Coefficient	95% Confidence Interval	Coefficient	95% Confidence Interval	Coefficient	95% Confidence Interval
Treatment	0.296***	[0.116, 0.484]	0.441	[-0.335, 1.028]	0.467	[-0.344, 1.050]
Maternal grandmother			-0.265**	[-0.528, -0.021]	-0.259**	[-0.503, -0.019]
Maternal grandmother*T			0.168	[-0.296, 0.615]	0.145	[-0.324, 0.575]
Paternal grandmother			0.008	[-0.200, 0.236]	0.006	[-0.192, 0.238]
Paternal grandmother*T			-0.253*	[-0.529, 0.029]	-0.235*	[-0.493, 0.039]
Mothers sisters			0.031	[-0.052, 0.103]		
Mothers sisters*T			-0.056	[-0.163, 0.057]		
Fathers sisters			0.002	[-0.140, 0.140]		
Fathers sisters*T			0.042	[-0.110, 0.188]		
Mothers brothers			-0.001	[-0.116, 0.082]		
Mothers brothers*T			0.024	[-0.109,0.170]		
Fathers brothers			0.016	[-0.064, 0.115]		
Fathers brothers*T			-0.034	[-0.148, 0.075]		
Total siblings of mother					0.016	[-0.032, 0.062]
Total siblings of mother*T					-0.019	[-0.069, 0.037]
Total siblings of father					0.01	[-0.038, 0.054]
Total siblings of father*T					0.001	[-0.080, 0.072]
R-Squared	0.19		0.195		0.193	
N	2017		2017		2017	

Table notes: OLS regressions with height for age (HAZ) scores as dependent variable. Model 1 estimates the overall effect of exposure to the program. Models 2 and 3 estimate regressions that allow the program effect to vary with different extended family members. Inference is conducted using the wild cluster bootstrap-t procedure recommended by Cameron et al (2008). 95% confidence intervals calculated according to the method recommended in Cameron and Miller (2015).

All regressions include the following controls: Cluster level controls: Education and Chewa ethnicity in 2004, Household level controls: A wealth index calculated in 2004, Mother level controls: Whether she had completed primary school, was working as a farmer or was a student in 2004, current age, age^2 and logarithmic height. Child level controls: Month of measurement, age, age^2, gender, number of older siblings, number of older $siblings^2$.

Sample includes all children born after the intervention start date in July 2005 to married main respondent mothers, who were aged 0-53 months at the time of measurement. Column 1 indicates the effect of intervention assignment on HAZ scores, for the sample where family networks information is not missing. Models 2-3 indicate how intervention effects on HAZ scores vary with the presence of different extended family members. *= p<0.1, **=p<0.05, ***=p<0.01

For peer review only

Appendix 1

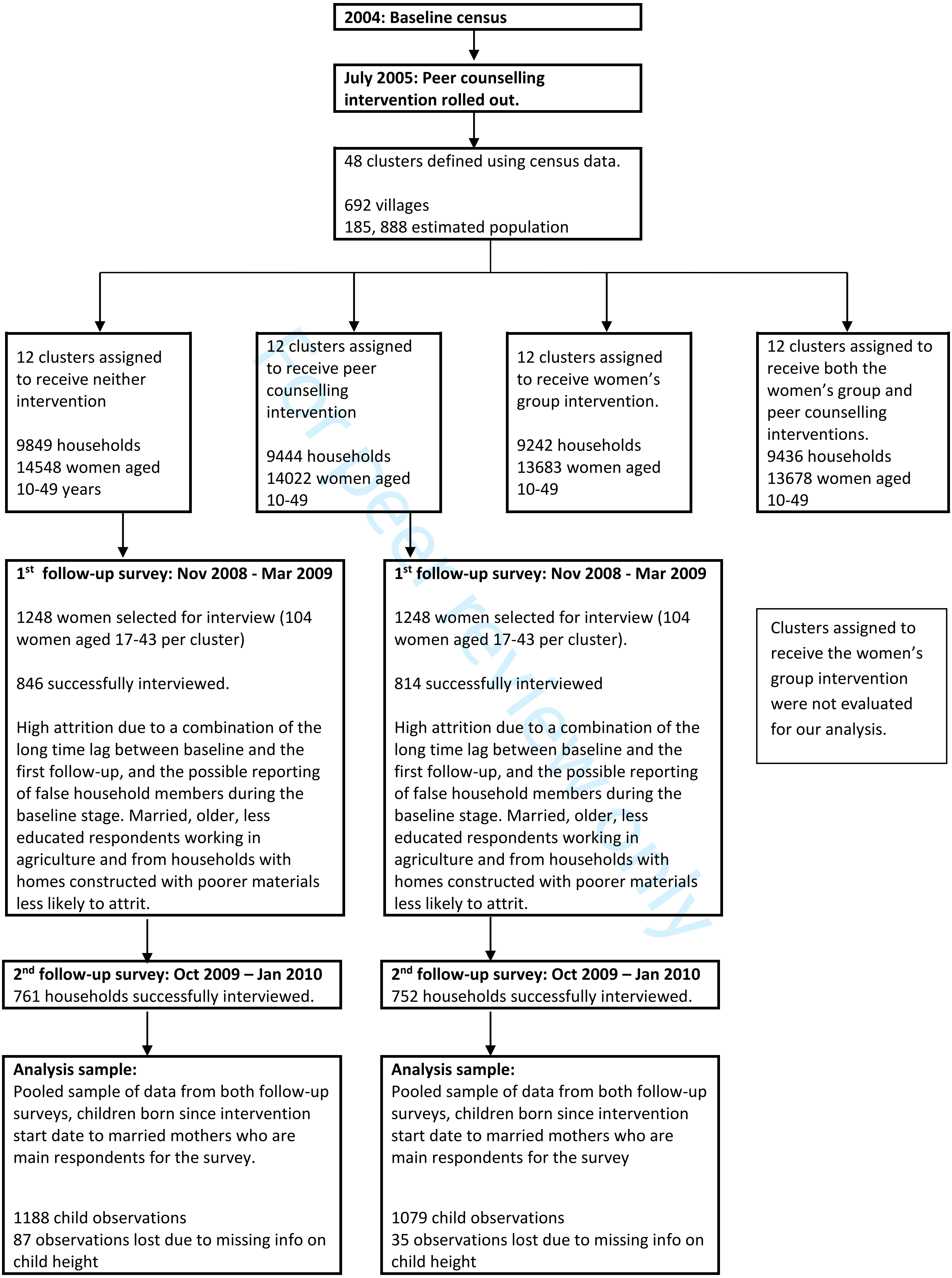

For peer review only

STROBE Statement—Checklist of items that should be included in reports of ***case-control studies***

	Item No	Recommendation	Complete?
**Title and abstract**	1	(*a*) Indicate the study's design with a commonly used term in the title or the abstract	✓
		(*b*) Provide in the abstract an informative and balanced summary of what was done and what was found	✓ (pg 2)
**Introduction**			
Background/rationale	2	Explain the scientific background and rationale for the investigation being reported	✓ (pg 4-5)
Objectives	3	State specific objectives, including any prespecified hypotheses	✓ (pg 6)
**Methods**			
Study design	4	Present key elements of study design early in the paper	✓ (pg 6 – 7)
Setting	5	Describe the setting, locations, and relevant dates, including periods of recruitment, exposure, follow-up, and data collection	✓ (pg 6 - 9, 12 - 13)
Participants	6	(*a*) Give the eligibility criteria, and the sources and methods of case ascertainment and control selection. Give the rationale for the choice of cases and controls	✓ (pg 6-9)
		(*b*) For matched studies, give matching criteria and the number of controls per case	
Variables	7	Clearly define all outcomes, exposures, predictors, potential confounders, and effect modifiers. Give diagnostic criteria, if applicable	✓ (pg 8-9)
Data sources/ measurement	8*	For each variable of interest, give sources of data and details of methods of assessment (measurement). Describe comparability of assessment methods if there is more than one group	✓ (pg 8-9)
Bias	9	Describe any efforts to address potential sources of bias	✓ (pg 9-12)
Study size	10	Explain how the study size was arrived at	
Quantitative variables	11	Explain how quantitative variables were handled in the analyses. If applicable, describe which groupings were chosen and why	✓ (pg 8-9)
Statistical methods	12	(*a*) Describe all statistical methods, including those used to control for confounding	✓ (pg 9-12)
		(*b*) Describe any methods used to examine subgroups and interactions	✓ (pg 9-12)
		(*c*) Explain how missing data were addressed	✓ (pg 8-9)
		(*d*) If applicable, explain how matching of cases and controls was addressed	
		(*e*) Describe any sensitivity analyses	✓ (pg 9-12)
**Results**			
Participants	13*	(a) Report numbers of individuals at each stage of study—eg numbers potentially eligible, examined for eligibility, confirmed eligible, included in the study, completing follow-up, and analysed	✓ (Appendix 1)
		(b) Give reasons for non-participation at each stage	✓ (Appendix 1)
		(c) Consider use of a flow diagram	✓ (Appendix 1)
Descriptive data	14*	(a) Give characteristics of study participants (eg demographic, clinical,	✓ (Table 1)

For peer review only

		social) and information on exposures and potential confounders	
		(b) Indicate number of participants with missing data for each variable of interest	✓ (Appendix 1)
Outcome data	15*	Report numbers in each exposure category, or summary measures of exposure	✓ (Table 1)
Main results	16	(*a*) Give unadjusted estimates and, if applicable, confounder-adjusted estimates and their precision (eg, 95% confidence interval). Make clear which confounders were adjusted for and why they were included	✓ (Table 4)
		(*b*) Report category boundaries when continuous variables were categorized	✓
		(*c*) If relevant, consider translating estimates of relative risk into absolute risk for a meaningful time period	
Other analyses	17	Report other analyses done—eg analyses of subgroups and interactions, and sensitivity analyses	✓ (Table 4)
**Discussion**			
Key results	18	Summarise key results with reference to study objectives	✓ (pg 14 - 16)
Limitations	19	Discuss limitations of the study, taking into account sources of potential bias or imprecision. Discuss both direction and magnitude of any potential bias	✓ (pg 19)
Interpretation	20	Give a cautious overall interpretation of results considering objectives, limitations, multiplicity of analyses, results from similar studies, and other relevant evidence	✓ (pg 16 – 19)
Generalisability	21	Discuss the generalisability (external validity) of the study results	✓ (pg 16-19)
**Other information**			
Funding	22	Give the source of funding and the role of the funders for the present study and, if applicable, for the original study on which the present article is based	✓ (pg 20)

*Give information separately for cases and controls.

Note: An Explanation and Elaboration article discusses each checklist item and gives methodological background and published examples of transparent reporting. The STROBE checklist is best used in conjunction with this article (freely available on the Web sites of PLoS Medicine at http://www.plosmedicine.org/, Annals of Internal Medicine at http://www.annals.org/, and Epidemiology at http://www.epidem.com/). Information on the STROBE Initiative is available at http://www.strobe-statement.org.

For peer review only

For peer review only - http://bmjopen.bmj.com/site/about/guidelines.xhtml

BMJ Open

Family networks and infant health promotion: a mixed-methods evaluation from a cluster randomised controlled trial in rural Malawi

Journal:	*BMJ Open*
Manuscript ID	bmjopen-2017-019380.R2
Article Type:	Research
Date Submitted by the Author:	27-Mar-2018

Complete List of Authors:	Scott, Molly; Oxford Policy Management<br>Malde, Bansi; University of Kent, School of Economics; Insitute for Fiscal Studies, Centre for Evaluation of Development Policies<br>King, C.; UCL, Institute for Global Health<br>Phiri, Tambosi; MaiMwana Project,<br>Chapota, Hilda; MaiMwana Project,<br>Kainja, Esther; MaiMwana Project,<br>Banda, Florida; MaiMwana Project<br>Vera-Hernandez, Marcos; UCL; Insitute for Fiscal Studies, Centre for Evaluation of Development Policies

**Primary Subject Heading**:	Global health
Secondary Subject Heading:	Health policy
Keywords:	Nutrition < TROPICAL MEDICINE, Extended Family, Health promotion, Sub-Saharan Africa, Child Health

SCHOLARONE™
Manuscripts

Family networks and infant health promotion: a mixed-methods evaluation from a cluster randomised controlled trial in rural Malawi

Authors:

Molly Scott[1], Bansi Malde[3,6]*, Carina King[4], Tambosi Phiri[5], Hilda Chapota[5], Esther Kainja[5], Florida Banda[5], Marcos Vera-Hernandez[2,3]

Affiliations:

1. Oxford Policy Management
2. Department of Economics, University College London, UK
3. Centre for Evaluation of Development Policies, Institute for Fiscal Studies, London, UK
4. Institute for Global Health, University College London, UK
5. MaiMwana Project, Mchinji, Malawi
6. School of Economics, University of Kent, UK

***Corresponding author:**

School of Economics, University of Kent, Canterbury, Kent CT2 7NZ, UK. E-mail: b.k.malde@kent.ac.uk; Telephone: (+44)(0)1227816464

Keywords: Extended Family; Child Health; Sub-Saharan Africa; Health Promotion; Child Nutrition

For peer review only - http://bmjopen.bmj.com/site/about/guidelines.xhtml

For peer review only

Abstract:

Objective: Parents may rely on information provided by extended family members when making decisions concerning the health of their children. We evaluate whether extended family members affected the success of an information intervention promoting infant health.

Methods: This is a secondary, sequential mixed-methods study based on a cluster randomised controlled trial of a peer home-education intervention conducted in Mchinji District, Malawi. We used linear multivariate regression to test whether the intervention impact on child height-for-age z-scores (HAZ) was influenced by extended family members. 12 of 24 clusters were assigned to the intervention, in which all pregnant women and new mothers were eligible to receive 5 home visits from a trained peer counsellor to discuss infant care and nutrition. We conducted focus group discussions with mothers, grandmothers and peer counsellors, and key-informant interviews with husbands, chiefs and community health workers to better understand the roles of extended family members in infant feeding.

Results: Exposure to the intervention increased child HAZ scores by 0.296 standard deviations (SD) (95% CI: 0.116, 0.484). However, this effect is smaller in the presence of paternal grandmothers. Compared to an effect size of 0.441 – 0.467 SD (95% CI: -0.344, 1.050) if neither grandmother is alive, the effect size was 0.235 (95% CI: -0.493, 0.039) to 0.253 (95% CI: -0.529, 0.029) SD lower if the paternal grandmother was alive. There was no evidence of an effect of parents' siblings. Maternal grandmothers did not affect intervention impact, but were associated with a lower HAZ score in the control group. Qualitative analysis suggested that grandmothers, who act as secondary caregivers and provide resources for infants, were slower to dismiss traditionally held practices and adopt intervention messages.

Conclusion: The results indicate that the intervention impacts are diminished by paternal grandmothers. Intervention success could be increased by integrating senior women.

Strengths and Limitations:

For peer review only - http://bmjopen.bmj.com/site/about/guidelines.xhtml

- Uses mixed methods to understand how extended family members affected the success of an infant feeding promotion program in Malawi
- Quantitative analysis, using linear multivariate regression, allows estimation of the size of effect different extended family members – including paternal and maternal grandmothers – have on the impact of the intervention
- Focus group discussions and key informant interviews help shed light on the mechanisms through which extended family members might have affected the intervention's success
- The interval between qualitative and quantitative data collection is a limitation, with potential changes to infant feeding practices over time and recall bias.
- Interventions on infant feeding would benefit from understanding and addressing extended family dynamics to improve reach and impact

For peer review only

For peer review only - http://bmjopen.bmj.com/site/about/guidelines.xhtml

Introduction

Child health outcomes are influenced by individuals besides the mother and father, with a rich literature devoted to the contribution of extended family members [1], [2], [3], [4], [5]. In low-income settings, where risk of poor health is high and social welfare nets are minimal, the support of the extended family may be crucial in child-rearing [6]. Relatives can assist by acting as secondary caregivers [7], supplying labour, donating money and providing other in-kind resources to the household [8], [9], [10], [1], [11]. Older women in the family can also affect child outcomes by dispensing infant health and nutrition advice to new mothers [12], [4], [13], [14], [5]. Finally, relatives may exert pressure on mothers, impacting their infant nutrition and health practices [15].

Extended family members may influence the effectiveness of policies and interventions designed to improve child health in low-income settings. On the one hand, they may provide resources and support that complement the intervention making it more effective. On the other hand, they could be resistant to change and reinforce traditional practices, thereby undermining interventions. The latter is particularly relevant in the case of health education and outreach programs, which are widespread across both developed and developing country settings.

Educational programs have the potential to improve child health outcomes by changing widespread misconceptions and traditional behaviours around child feeding and care in low-income settings. In Malawi, for instance, although most infants are breastfed for at least a year, only 40.5% of infants are still exclusively breastfed at 5 months [16]; while diets of children aged over 6 months usually lack sufficient diversity [17], [18]. Beliefs surrounding feeding practices for older infants include the view that the broth of soup is more nourishing than the vegetables or meat inside and that eggs are harmful for children aged 9 months.

For peer review only - http://bmjopen.bmj.com/site/about/guidelines.xhtml

Education campaigns promoting better infant feeding and care to care-givers of infants have shown mixed success in developing country settings. In some cases, they have led to sustained improvements in feeding practices [19], [14] and child physical growth [20], [21], [22]. In others, they have had a negligible effect on child physical growth [23], [24], [25]. Given these mixed findings, there is a need for greater understanding of the factors shaping responses to such interventions across contexts.

Little attention has been paid to the role of extended family members in influencing the success of care-giver focused education interventions in the existing literature, despite their important role in shaping child health. Much of this existing literature is qualitative, with small samples [26], [27]. The small number of quantitative evaluations of education interventions to improve infant feeding that seek to involve extended family members find mixed evidence of effectiveness. Counselling sessions for new adolescent mothers and co-resident grandmothers reduced the un-necessary intake of water and herbal teas within the first 6 months of the child's life in Brazil [28] but failed to maintain breastfeeding of infants at age 2 years [29]; while a behavioural change communication program delivered through older female leaders in Burkina Faso improved infant feeding knowledge but failed to improve child health outcomes [14].

A cluster randomised controlled trial (RCT) conducted in Malawi used a peer home-education strategy to improve rates of exclusive breastfeeding, and reduce infant mortality (see Lewycka et al. 2010, 2013 for further details [30], [31]). The trial achieved a 36% reduction in infant mortality and an improvement in children's height-for-age z-score (HAZ) (increased by 0.271 standard deviations; p=0.022) [17] [31]. This paper uses mixed-methods to investigate whether members of the extended family influenced the success of this peer home visiting intervention, and the possible mechanisms through which their influence might work.

For peer review only - http://bmjopen.bmj.com/site/about/guidelines.xhtml

Methods:

This is a secondary, sequential mixed-methods study based on a cluster RCT of a peer home-education intervention conducted in Mchinji District, Malawi. We investigate family member roles in the success of the intervention, which provided information on healthy infant feeding practices, with quantitative data collected between November 2008 and January 2010, and qualitative data collected in December 2015. Full details of the original trial and methods have previously been published [30] [31]. The quantitative data was collected and analysed first, and used to design the qualitative aspect of the study. The overall interpretation of our findings was integrated following analysis of the qualitative data.

Setting:

Mchinji is a rural district in central Malawi with a population of about 455,000 [32]. Maternal and infant healthcare is delivered at one district hospital, four rural hospitals, nine health centres, private clinics, and in the community through government employed community health workers (CHW – known locally as Health Surveillance Assistants). Much of the healthcare received by pregnant women and infants is in the community setting by CHWs, or at home by kin and other social contacts. However, Malawi has medical pluralism, with traditional practices, beliefs and behaviours such as witchcraft and herbal medicine being commonly used alongside Western medicine. The 2010 Malawi Demographic and Health Survey reported 24% of births in the region occurred in the woman's own home and 2% in someone else's home, and many births are not attended by medically trained healthcare personnel but by traditional birth attendants (14.4%), friends and relatives (8.7%) or no one (2.6%) [16]. Without access to a skilled birth attendant, women are more vulnerable to infection and complications during birth; the infant mortality rate in 2010 was of 66 per 1000 live births [16].

For peer review only - http://bmjopen.bmj.com/site/about/guidelines.xhtml

Traditionally, the main ethnic group in the study area, the Chewa, are a matrilineal and matrilocal group. Matriliny is a system in which land is passed through the female line. Under traditional matrilocal norms, husbands move to their wives' homes after marriage unless they make a special payment. However, following the influence of patrilineal and patrilocal ethnic groups and British colonialists, there is evidence that matrilocality has waned over time, but not completely [33], [34]. As a result, women often remain in close proximity to their own relatives after marriage.

Intervention Description

For the RCT, Mchinji was divided into 48 approximately equal population clusters based on the 1998 Malawi Population and Housing census (the most recent census at the time of trial planning). Within each cluster of around 8000 people, the 3000 individuals living in villages closest to the geographical centre were enumerated as the eligible study population. 12 clusters were assigned to the infant feeding intervention only and 12 served as controls. Full details of the trial set-up and methods are in Lewycka et al., 2010 [30]. All women living in clusters assigned to the infant feeding intervention who became pregnant during the trial period were eligible to receive five home visits from a trained local woman volunteer ('peer counsellor') to discuss maternal and infant healthcare issues; around 60% of eligible women reported having been visited. The visits were timed to coincide with key stages of infant development (the third trimester, and at one week, one month, three and five months after birth). Each visit focused on a specific set of topics for discussion, with special attention paid to nutrition practices including exclusive breastfeeding, and complementary feeding. Peer counsellors were literate local women aged 23-50 years with breastfeeding experience, who each covered a population of about 1000 people.

The intervention began in December 2004, with an initial establishment period until June 2005. The trial was on-going at the time of the quantitative data collection. Following the end of the trial period, peer counsellors continued to receive mentorship and supervision support from government CHWs and the local implementing NGO. In 2015, at the time of qualitative data

For peer review only - http://bmjopen.bmj.com/site/about/guidelines.xhtml

collection, approximately 1/3 of the volunteer counsellors were still active in delivering the intervention.

Quantitative Analysis:

Data and Sample Selection:

A baseline census was conducted in all clusters in 2004, prior to the start of the intervention. All women aged between 10 and 49 were enumerated and a random sample of 104 women aged between 17 and 43 per cluster was then drawn to be interviewed for two follow-up quantitative surveys as part of this secondary study. Sampled women (`main respondent' hereon) were visited to complete the first follow-up in November 2008-March 2009, and a second follow-up in October 2009 –January 2010.

Each follow-up survey contained questions about the size of the extended family of the main respondent and her husband (those alive and those in the village), the health of all household members, food and liquid intake of children aged under 6 years, knowledge about child nutrition, intervention participation (in treatment clusters), and socio-economic variables such as adult work. The height of the main respondent and the height and weight of children under 6 years were also collected by trained enumerators.

The main outcome for our analysis is the child height-for-age z-score (HAZ score), which is a long-term indicator of health that reflects nutrition and morbidity since birth, and should be sensitive to any effects of intervention exposure in early life. It is calculated by comparing the height of the child with the median height in the World Health Organization reference population of children of the same gender and age in months [35].

The sample was balanced between treatment and control clusters along a range of variables collected at baseline (see Fitzsimons et al (2016), Table 1 [17]). The baseline characteristics of the

For peer review only - http://bmjopen.bmj.com/site/about/guidelines.xhtml

two groups remained similar even after accounting for attrition between the baseline and first endline survey, indicating that randomisation was not jeopardised.

For this analysis we use a sample of children who were born since July 2005; and whose mothers are married main respondents in the follow-up surveys (80% of the sample). This sample selection ensures that we measure effects on children whose mothers were eligible to receive visits from a peer counsellor; and allows us to compare effects of the mothers' relatives with those of her husband. Children in the estimation sample were aged between 0 and 53 months at the time of the endline surveys. Appendix 1 provides a timeline of the original trial and the quantitative data collection, and of our sample inclusion criteria.

Table 1 presents the means of basic demographic and socio-economic characteristics for women in the analysis sample living in control clusters at baseline, the differences in the means between the control and treatment groups, and the p-value of this difference. The last two columns allow us to assess whether the randomisation holds in our selected sample. Women assigned to the control group were 24.6 years on average. 71.8% were married and while 70.1% had completed at least primary education, only 7.6% had completed secondary education. In line with the general profile of communities in Mchinji, 95.4% of sampled women were Chewa ethnicity and 98.3% were Christian. The average household size was 5.6 members and all households were engaged in agricultural activity.

Table 2 displays statistics on the size of extended family networks of the children in our analysis sample. Most children have their grandmothers alive (87.3% have maternal grandmothers alive and 80.7% have paternal grandmothers) and their parents have a relatively large number of siblings, with an average of more than two brothers and two sisters each.

Model Specification and Estimation

For peer review only - http://bmjopen.bmj.com/site/about/guidelines.xhtml

For peer review only

The quantitative analysis aims to determine how different family members influence the effectiveness of the infant feeding intervention. Before estimating the main model, we study the relationship between baseline characteristics of mothers and their households and measures of the extended family using linear regression. Table 3 reports these results. It indicates that children whose grandmothers are alive have on average younger mothers, who are more likely to have completed at least primary education, less likely to be working as farmers in 2004, and are from more socioeconomically advantaged households, as measured by a composite wealth index constructed using principal components analysis as recommended by [36].

Our main specification is the following linear multivariate regression:

$$\begin{aligned} HAZ_{ij} = {} & \alpha + \beta T_j + \beta_2 Maternal\_grandmother_{ij} + \beta_3 Maternal\_grandmother_{ij}{}^{*}T_j \\ & + \beta_4 Paternal\_grandmother_{ij} + \beta_5 Paternal\_grandmother_{ij}{}^{*}T_j \\ & + \beta_6 Total\_mothers\_siblings_{ij} + \beta_7 Total\_mothers\_siblings_{ij}{}^{*}T_j \\ & + \beta_8 Total\_fathers\_siblings_{ij} + \beta_9 Total\_fathers\_siblings_{ij}{}^{*}T_j + X_{ij}\gamma \\ & + Z_j\gamma_2 + t + \epsilon_{ij} \end{aligned}$$

where HAZ_{ij} is the height-for-age z-score of child i in cluster j. T_j is a treatment exposure indicator which captures whether the child was born to a mother living in 2004 (pre-intervention) in a cluster that was assigned to receive the program. We therefore use an intent-to-treat estimator. $Maternal\_grandmother_{ij}$ and $Paternal\_grandmother_{ij}$ are binary variables indicating, respectively, whether the maternal and paternal grandmother is alive. $Total\_mothers\_siblings_{ij}$ ($Total\_fathers\_siblings_{ij}$) captures the total number of siblings of the child's mother (father) who are alive. We use two definitions of this variable in different specifications of the model: (i) Brothers and sisters (separately) of each parent, and (ii) the total siblings of each parent. X_{ij} and Z_j are vectors of control variables at the individual and cluster level respectively. These include all baseline characteristics where significant differences between households with different

For peer review only - http://bmjopen.bmj.com/site/about/guidelines.xhtml

extended family members alive were detected, and interview month and year indicators to account for month-year-specific shocks. We do not adjust the data for missing information.

We fitted three models, one crude model with the intervention term only, and two full models as specified in the equation each treating parent siblings differently.

The coefficient β captures the effect of the program for children whose maternal and paternal grandmothers are dead, and whose parents are only children, while the coefficients β_2, β_4, β_6 and β_8, represent the effects of the extended family members on HAZ scores in the control group. The coefficients β_3, β_5, β_7 and β_9, associated with interaction terms between variables capturing extended family relations and the indicator for program allocation, estimate the additional effect of the program for children with different types and numbers of extended family members. A positive (or negative) significant interaction provides evidence that the program effect is enhanced (or diminished) in the presence of that particular family member.

Errors ε_{ij} are assumed to be uncorrelated between individuals in different clusters but are allowed an unrestricted correlation structure within clusters. To account for correlation within clusters, standard errors must be adjusted to prevent downward bias, and incorrect inference. Given the small number of clusters in the study (12 intervention and 12 control clusters), we adopt wild cluster bootstrap methods as recommended in Cameron et al. (2008) [37]. Associated 95% confidence intervals can be calculated using a computationally intensive method suggested in [38]. The bootstrap adjustment applied here was studied in detail by Fitzsimons et al (2016) and was found to perform well [17]. Data from both follow-up surveys is pooled to improve statistical power.

The extended family network is defined according to which members of the family are alive, rather than which ones live in the same village or household. This is in case treatment exposure affected decisions over where to live, which would cause a measure of family network size based

For peer review only - http://bmjopen.bmj.com/site/about/guidelines.xhtml

on residence to be correlated with the intervention, and thereby bias estimates. The benefit of defining the size of the family network according to which members are alive is that this is almost certain to be invariant to program exposure.

We choose to define T_j by exposure to the intervention rather than actual participation since participation in the program was voluntary and also relied on the ability of peer counsellors to locate eligible women. Women who peer counsellors did not manage to trace or who chose not to take part in the program may be different from those who did participate. The existence of such systematic differences would potentially introduce some unobserved correlation between the treatment interaction variables and HAZ scores if T_j were defined on the basis of actual participation. Indeed, Fitzsimons et al. (2016) report that women who received the visits tend to be poorer [17]. Defining treatment based on residence at baseline rather than at the time of the follow-up interviews also alleviates concerns of bias in case there was purposeful migration into treated areas by control-group assigned households.

Qualitative Analysis

Following the findings from the quantitative analysis, we conducted focus group discussions (FGDs) with grandmothers, mothers and peer counsellors, and semi-structured interviews with fathers, CHWs and village chiefs to gain a more in-depth understanding of family roles and how grandmothers might influence child health.

Recruitment

Participants were recruited from 11 of 24 intervention and control clusters across the district in late 2015. Mothers, grandmothers and fathers were purposively selected by CHWs and chiefs to represent those households who had actively received the intervention or had children under-5 years in control clusters. Volunteer peer counsellors were contacted directly, to represent a range of ages and years' experience as counsellors. Chiefs and CHWs were purposively selected and

For peer review only - http://bmjopen.bmj.com/site/about/guidelines.xhtml

contacted directly to represent clusters with a range of engagement with the intervention. We planned to conduct a total of five FGDs and 10 interviews, rather than collecting data until saturation was reached.

Data Collection:

FGDs and interviews used topic guides, based on the quantitative findings and wider literature on infant feeding behaviours, covering: household decision making around feeding, infant feeding practices, feeding knowledge and sources of information about infant feeding. We asked about all household members, and specifically probed about the role of grandmothers. All discussions were facilitated by two local trained qualitative researchers in Chichewa. Participants were reimbursed for their travel expenses and given refreshments. All discussions were audio recorded, and then verbatim transcribed in Chichewa. Transcripts were translated into English as a group, with ambiguous terms or phrases debated until a consensus meaning was reached. Data collection, transcription and translation were conducted by EK, HC, TP and FM – female Malawian researchers who are fluent in English.

Analysis

The English transcripts were coded using an inductive framework approach based on the following steps: familiarisation, coding, developing and applying the framework, charting and interpretation [39]. All transcripts were double-coded, as a group by TP, EK, HC and FB and independently by CK – a female British researcher with five years' work experience in Malawi. Coding was done on paper and the coding matrix developed in Microsoft Excel. A round-table discussion was then conducted by all five researchers to compare the codes and agree on themes; disagreements in coding were discussed until an agreement on the interpretation was reached.

Patient and Public Involvement

For peer review only - http://bmjopen.bmj.com/site/about/guidelines.xhtml

The original trial was conducted with extensive community engagement, including initial planning and dissemination meetings with village, healthcare and local government committees. These groups were involved in the recruitment of participants for interviews and focus group discussions. The quantitative survey instruments were pre-tested on households living in buffer areas.

Ethics

All participants gave informed written consent. Ethical approval to conduct this study was obtained from the National Health Sciences Research Committee in Malawi [Protocol Numbers: 491; 15/9/1483].

Results

Quantitative Analysis:

Table 4 displays the results of the quantitative analysis on child HAZ scores. Model 1 shows that overall exposure to the program raised HAZ scores by 0.296 (95% CI: 0.116, 0.484) standard deviations (SD). Models 2 and 3 present the results for the regressions that test whether different family members influenced the effectiveness of the intervention on child HAZ scores. Model 2 presents the results where we allow for brothers and sisters of the child's parents to have different effects, while Model 3 displays those including (separately) the total siblings of each of the child's parents.

For children whose parents have no living mothers or siblings, the effect of the intervention on HAZ scores is between 0.441 (95% CI: -0.335, 1.028) and 0.467 (95% CI: -0.344, 1.050) SD. However, for those children with a living paternal grandmother, the intervention effect was reduced by between 0.235 (95% CI: -0.493, 0.039) and 0.253 (95% CI: -0.529, 0.029) SD. The results also suggest that, in the control group, children whose maternal grandmothers are alive have HAZ scores that are between 0.259 (95% CI: -0.0503, -0.019) and 0.265 (95% CI: -0.528, -0.021) SD lower. The coefficient on the interaction term between having a living maternal

For peer review only - http://bmjopen.bmj.com/site/about/guidelines.xhtml

grandmother and the indicator for the intervention allocation is positive, but statistically insignificant at the 10% level of significance (magnitude of between 0.145 (95% CI: -0.324, 0.575) and 0.168 (95% CI: -0.296, 0.615) SDs). Finally, the results uncover no association between the number of parents' siblings on HAZ scores, or of differential effects of the intervention by these.

Qualitative Analysis:

We conducted 5 FGDs, with 37 participants of 48 invited (mothers=16; grandmothers=15; peer counsellors=6), and 10 semi-structured interviews (village chiefs=4, fathers=4; CHW=2). We defined the following emergent themes in relation to grandmothers and their role in infant feeding and growth: decision-making roles, knowledge and information, traditional practices and intervention successes and challenges around behaviour change.

Decision-Making Roles: Across the respondents there was agreement that the father is responsible for resource allocation and mobilisation, while the mother's role is to manage and prepare food for the household. When there is a lack of food or resources, extended family members or neighbours can provide assistance, for example "*maybe you have found you don't even have flour, our sister in law or mother in law gives it to you saying that it's only for the child, prepare porridge so that it should eat"* (Mother 5, Control). Within the household, grandmothers, both maternal and paternal, were generally viewed as the secondary caregivers, providing support by cooking for and feeding infants.

Information and Knowledge: Sources of information about infant feeding included: antenatal clinics and other healthcare, family members, village chiefs and community meetings, the peer counsellor intervention and other NGOs and civil society education programmes. Interestingly, the peer counsellors were reported as a source of information by participants from control areas, likely reflecting contamination following the end of the trial period. Although grandmothers report giving similar advice as that given by healthcare workers:

For peer review only - http://bmjopen.bmj.com/site/about/guidelines.xhtml

"*now we are afraid, so we provide the same advice they give at the clinic, so we tell them the same things*" (Grandmother 7, Intervention),

However, reports from peer counselors cast doubt on this. They instead mentioned encountering difficulties with grandmothers when disseminating their advice: "*frequently the grandmothers mislead, mislead them as they say what they were doing before in their time*" (Peer Counselor 5).

Peer counselors, however, also noticed a change over time in attitudes among grandmothers, with increased acceptance of the intervention messages: "*the group of relatives which gives the most problems is the grandparents because they tell the woman that 'aaah [the counselors] are just cheating you, they want this child to be crying' [...] but we have seen that the grandmothers now have understood*" (Peer Counselor 4).

Despite extended family members not being the target group of the intervention, breastfeeding, weaning and complementary feeding messages appear to have disseminated, with fathers ("*advice about breast feeding, I know a lot; when a child is born he should breast feed exclusively, very frequently*" – Father 1, Intervention) and grandmothers ("*so they say breastfeed frequently these days, that's the modern way of childbirth, so you also say breastfeed the child*" – Grandmother 2, Intervention) demonstrating accurate knowledge.

Traditional Practices: Several different traditional practices and beliefs about infant feeding were mentioned by all respondent types, including: adding medicinal herbs to infants' porridge; believing children become "foolish" is breastfed for too long; and smaller portions making children "smart". However, mothers and grandmothers in both intervention and control areas commented that while these practices and beliefs are known to exist, they are no longer commonplace ("*most of this generation do not follow [these practices]*" – Mother 8, Control) or rituals and the giving of herbal medicines is done in hiding. This was confirmed by one of the CHW who commented that: "*while*

For peer review only - http://bmjopen.bmj.com/site/about/guidelines.xhtml

the grandmothers and the other people have their own beliefs, our role is to get rid of those beliefs […] little by little people change" (CHW 2, Intervention).

Behaviour Change: Community members reported sustained behaviour change relating to exclusive breastfeeding and facility-based deliveries: "*behaviour these days has changed in that delivering at home is no longer there [...] we say go to the hospital*" (Grandmother 5, Intervention). However, CHW and peer counselors noted that these changes were not seen immediately, and that barriers such as lack of engagement, lack of understanding and cultural issues (e.g. urban women 'looking down' on the counselors) were present.

Discussion:

Our mixed-methods evaluation of the effect of extended family members on the impact of a peer-led home education intervention in rural Malawi suggests that living paternal grandmothers can be a barrier to intervention dissemination and behaviour change. The qualitative findings complement the quantitative results, and suggest the mechanism through which grandmothers may influence the effectiveness of the peer intervention.

The apparently negative influence of paternal grandmothers on intervention success may be due to a conflict between their views on infant feeding from the recommendations of peer counsellors. The qualitative findings offer some support for this hypothesis by providing evidence that grandmothers are proponents of 'traditional' views of child feeding that differ from standard recommendations, supported by previous studies [4], [12]. They indicate that grandmothers persist in their traditional beliefs for longer, and as providers of both financial and childcare support, exert influence towards their own beliefs of child feeding rather than towards the information provided by the intervention. Reassuringly though, our qualitative data suggest that grandmothers eventually adjust their practices to be in line with the information provided by the intervention.

For peer review only - http://bmjopen.bmj.com/site/about/guidelines.xhtml

Interestingly, the qualitative data was not able to distinguish between paternal and maternal grandmothers, despite other evidence that, at least in Malawi, it is paternal grandmothers who command the most influence [12]. This may explain why we do not find a similar negative effect on intervention success associated with maternal grandmothers in the quantitative analysis. However, our ability to speculate on different mechanisms of action between maternal and paternal grandmothers is limited.

The delays seen in attitude change amongst grandmothers from the qualitative data suggest that there may be potential to increase the intervention's impact further by engaging extended family members in the information exchange process. A growing body of evidence underscores the benefits of more inclusive approaches to health education [40], [12], [13], [14], and cautions against assuming that new information will necessarily be incorporated into knowledge and behaviour. Actual response will, in general, depend on the mode of transmission. Approaches that treat users of information as passive are less likely to be effective than those that foster dialogue within the target communities.

In contexts where older women exert particular influence, there are clear grounds for designing interventions that respect and acknowledge their seniority. The rationale behind the work of organisations like the Grandmother Project is that elder women can act as powerful agents for change if they are mobilised and empowered to support intervention aims [4], [12]. We consider our findings to provide support for this agenda. However, it must be noted that involving senior women in interventions might not be sufficient to improve child health, particularly in contexts where poor nutrition is not the only cause for poor health. Evidence from the evaluation of an integrated agriculture and nutrition and health behaviour change communication programme indicates that senior women can be effective in changing knowledge, but this improved knowledge might still fail to yield improvements in child growth [14].

For peer review only - http://bmjopen.bmj.com/site/about/guidelines.xhtml

We uncover a negative association of maternal grandmothers in the control group. However, this cannot be taken as evidence of a causal effect, because of the presence of confounders such as a higher competition for resources in families with living maternal grandmothers in matrilineal societies [41].

The qualitative findings also raise the importance of the role of men as key providers and resource mobilisers. Previous quantitative evidence Fitzsimons et al (2016) [17] supports the critical role of males in ensuring adoption of the information provided. Therefore, integrating these influential figures with the peer counsellor intervention may help improve uptake and reduce the time to intervention acceptance we currently observe.

In this study there were several limitations. Firstly, it is impossible to ascertain whether the estimated quantitative detrimental effect of grandmothers is due to their presence and not because households in which the paternal grandmother is alive are different in some characteristic that is omitted from the regression and that affects the effectiveness of the intervention. The survey and qualitative data may be subject to social desirability bias, with respondents providing answers which they think will please the researchers. As the qualitative and quantitative data triangulated, and respondents were not aware of our hypothesis, we do not feel this considerably biased our conclusions. Finally, the mixed data were collected sequentially rather than concurrently, with the qualitative data collection conducted five years after the quantitative survey. This may have resulted in recall bias in the qualitative data, and the culture and behaviours around infant feeding may have shifted between the two study phases. This is somewhat supported by the FGDs and interviews from control areas being exposed to the peer counsellors and their messages, posing a challenge to integrating the results. However, as the qualitative data was planned to provide a more in-depth understanding and triangulation of the quantitative findings, rather than comment on causality, we do not feel this detracts from our interpretation.

For peer review only - http://bmjopen.bmj.com/site/about/guidelines.xhtml

We found that paternal grandmothers play an important role in shaping responses to an information campaign targeting infant health. In order to increase the impact of information campaigns, our findings suggest that excluding influential older women, who act as both important sources of advice and childcare support, can weaken intervention impact by exposing a divergence between traditional views and new information. Inclusive health education approaches that respect the need to tackle existing traditional beliefs and the roles that grandmothers play, may overcome this friction and improve the effectiveness of the intervention.

For peer review only

For peer review only - http://bmjopen.bmj.com/site/about/guidelines.xhtml

Acknowledgements: We thank Andrew Mganga, Nicholas Mbwana, Christopher Kamphinga, Sonia Lewycka, Mikey Rosato and Julia Behrman for advice and assistance with quantitative data collection. We also thank Ravi Somani for his assistance in coding the program to obtain the confidence intervals.

Funding: Funding from the ESRC/DFID grant ES/J009253/1 is gratefully acknowledged.

Conflict of interest: We declare that we have no conflicts of interest.

Authors contributions: All authors provided final approval of the version to be published. Concept and design of quantitative analysis: MS, BM and MV; analysis and interpretation of quantitative findings: MS, MV and BM; drafting the article and revising it critically: MS, BM, CK and MV. Concept and design of the qualitative study: BM, MVH, CK and TP. Qualitative data collection and analysis: TP, HC, EK, FB. Qualitative analysis was independently checked and coded: CK. CK, TP, EK, HC and FB discussed and agreed the final interpretation.

Data sharing: The raw quantitative data is available for download from the UK Data Archive.

For peer review only

References

[1] D. LaFave and D. Thomas, “Extended Families and Child Well-being,” *Journal of Development Economics,* vol. 126, no. C, pp. 52-65, 2017.

[2] P. Sheppard and R. Sear, “Do grandparents compete with or support their grandchildren? In Guatemala, paternal grandmothers may compete, and maternal grandmothers may cooperate,” *Royal Society Open Science,* vol. 3, no. 160069, 2016.

[3] B. Strassman and W. Garrard, “Alternatives to the grandmother hypothesis: A meta-anaalysis of the association between grandparental and grandchild survival in patrilineal populations,” *Human Nature,* vol. 22, no. 1, p. 201, 2011.

[4] J. Aubel, I. Touré and M. Diagne, “Senegalese grandmothers promote improved maternal and child nutrition practices: the guardians of traditional are not averse to change.,” *Social Science & Medicine, 59(5),* pp. 945-959, 2004.

[5] M. Gupta, R. Aborigo, P. Adongo, S. Rominski, A. Hogdson, C. Engmann and C. Moyer, “Grandmothers as gatekeepers? The role of grandmothers in influencing health-seeking for mothers and newborns in rural Ghana,” *Global Public Health,* vol. 10, no. 9, pp. 1078-1091, 2015.

[6] S. B. Hrdy, Mother nature: A history of mothers, infants, and natural selection., New York: Pantheon Books, 1999.

[7] P. K. Ivey, “Cooperative Reproduction in Ituri Forest Hunter-Gatherers: Who Cares for Efe Infants? 1.,” *Current Anthropology, 41.5,* pp. 856-866, 2000.

[8] M. A. Gibson and R. Mace, “Helpful Grandmothers in rural Ethiopia: A study of the effect of kin on child survival and growth,” *Evolution and Human Behaviour 26(6),* pp. 469-482, 2005.

[9] R. Lee and K. Kramer, “Children's Economic Roles in the Maya Family Life Cycle: Cain, Caldwell, & Chayanov Revisited,” *Population and Development Review, Vol. 28, No. 3,* pp. 475-499, 2002.

[10] M. Angelucci, G. De Giorgi, M. Rangel and I. Rasul, “Family networks and school enrolment: Evidence from a randomised social experiment.,” *Journal of Public Economics, 94(3),* pp. 197-221, 2010.

[11] E. Duflo, “Grandmothers and Granddaughters: Old-Age Pensions and Intrahousehold Allocation in South Africa,” *World Bank Economic Review,* vol. 17, no. 1, pp. 1-25, 2003.

[12] R. Bezner-Kerr, L. Dakishoni, L. Shumba, R. Msachi and M. Chirwa, “"We grandmothers know plenty": breastfeeding, complementary feeding and the multifaceted role of grandmothers in Malawi.,” *Social Science & Medicine, 66(5),* pp. 1095-1105, 2008.

For peer review only - http://bmjopen.bmj.com/site/about/guidelines.xhtml

For peer review only

[13] J. Aubel and D. Sihalathavong, “Participatory communication to strengthen to role of grandmothers in child health: an alternative paradigm for health education and health communication.,” *Journal of International Communication, 7(2),* pp. 76-97, 2001.

[14] D. K. Olney, A. Pedehombga, M. T. Ruel and A. Dillon, “A 2-Year Integrated Agriculture and Nutrition and Health Behavior Change Communication Program Targeted to Women in Burkina Faso Reduces Anemia, Wasting and Diarrhea in Children 3-12.9 Months of Age at Baseline: A Cluster-Randomized Controlled Trial,” *The Journal of Nutrition,* vol. 145, no. 6, pp. 1317 - 1324, 2015.

[15] O. Agunbiade and O. Ogunleye, “Constraints to exclusive breastfeeding practice among breastfeeding mothers in Southwest Nigeria: implications for scaling up,” *International Breastfeeding Journal,* vol. 7, no. 1, p. 5, 2012.

[16] National Statistical Office, Malawi, Macro ORC, “Malawi Demographic and Health Survey,” Zomba: Malawi National Statistical Office, Macro ORC, 2004.

[17] E. Fitzsimons, B. Malde, A. Mesnard and M. Vera-Hernandez, “Nutrition, Information, and Household Behaviour: Experimental Evidence from Malawi,” *Journal of Development Economics,* vol. 122, pp. 113-126, 2016.

[18] B. F. Kalanda, F. H. Verhoeff and B. J. Brabin, “Breast and complementary feeding practices in relation to morbidity and growth in Malawian infants.,” *European journal of clinical nutrition, 60(3),* pp. 401-407, 2006.

[19] A. L. Morrow, M. L. Guerrero, J. Shults, J. Calva, C. Lutter, J. Bravo, G. Ruiz-Palacios, R. Morrow and F. Butterfoss, “Efficacy of home-based peer counselling to promote exclusive breastfeeding: a randomised controlled trial.,” *The Lancet, 353(9160),,* pp. 1226-1231, 1999.

[20] G. Guldan, H. M. X. Fan, Z. Ni, X. Xiang and M. Tang, “Culturally appropriate nutrition education improves infant feeding and growth in rural Sichuan, China,” *Journal of Nutrition,* vol. 130, no. 5, pp. 1204-11, 2000.

[21] N. Bhandari, S. Mazumder, R. Bahl, J. Martines, R. Black, M. Bhan and I. F. S. Group, “An educational intervention to promote appropriate complementary feeding practices and physical growth in infants and young children in rural Haryana, India,” *Journal of Nutrition,* vol. 134, no. 9, pp. 2342-8, 2004.

[22] M. Penny, H. Creed-Kanashiro, R. Robert, M. Narro, L. Caulfield and R. Black, “Effectiveness of an educational intervention delivered through the health services to improve nutrition in young children: a cluster-randomised controlled trial,” *The Lancet,* vol. 365, no. 9474, pp. 1863-72, 2005.

[23] I. Engebretsen, D. Jackson, L. Fadnes, V. Nankabirwa, A. Diallo, T. Doherty, C. Lombard, S. Swanvelder, J. Nankunda, V. Ramokolo, D. Sanders, H. Wamani, N. Meda, J. Tumwine, E. Ekstrom, P. Van de Perre, C. Kankasa, H. Sommerfelt and T. Tylleksar, “Growth effects of

For peer review only - http://bmjopen.bmj.com/site/about/guidelines.xhtml

exclusive breastfeeding promotion by peer counsellors in sub-Saharan Africa: the cluster-randomised PROMISE EBF trial," *BMC Public Health,* vol. 14, no. 1, p. 633, 2014.

[24] N. Bhandari, R. Bahl, B. Nayyar, P. Khokhar, J. Rohde and M. Bhan, "Food supplementation with encouragement to feed it to infants from 4 to 12 months of age has a small impact on weight gain," *Journal of Nutrition,* vol. 131, no. 7, pp. 1946-51, 2001.

[25] A. Guyon, V. Quinn, Z. Rambeloson and M. Hainsworth, "Using the Essential Nutrition Actions Approach to Improve the Nutritional Practices of Women and Children at Scale in Antananarivo and Fianarantsoa Provinces of Madagascar: Results and Trends 2000 to 2005," USAID, 2006.

[26] A. Mukuria, S. Martin, T. Egondi, A. Bingham and F. Thuita, "Role of social support in improving infant feeding practices in Western Kenya: A Quasi-Experimental Study," *Global Health, Science and Practice,* vol. 4, no. 1, pp. 55-72, 2016.

[27] A. DeLorme, E. Gavenus, C. Salmen, G. Benard, B. Mattah, E. Bukusi and K. Fiorella, "Nourishing networks: A socio-ecological analysis of a network intervention for improving household nutrition in Western Kenya," *Social Science and Medicine,* vol. 197, pp. 95-103, 2018.

[28] L. Nunes, E. Giugliani, L. Santo and L. de Oliveira, "Reduction of unnecessary intake of water and herbal teas on breast-fed infants: a randomized clinical trial with adolescent mothers and grandmothers," *Journal of adolescent health,* vol. 49, no. 3, pp. 258-64, 2011.

[29] C. da Silva, L. Nunes, R. Schwartz and E. Giugliani, "Effect of a pro-breastfeeding intervention on the maintenance of breastfeeding for 2 years or more: randomized clinical trial with adolescent mothers and grandmothers," *BMC Pregnancy and Childbirth,* vol. 16, no. 3, p. 97, 2016.

[30] S. Lewycka, C. Mwansambo, P. Kazembe, T. Phiri, A. Mganga, M. Rosato, H. Chapota, F. Malamba, S. Vergnano, M. Newell, D. Orsin and A. Costello, "A cluster randomised controlled trial of the community effectiveness of two interventions in rural Malawi to improve health care and to reduce maternal, newborn and infant mortality," *Trials, 11(1), 88,* 2010.

[31] S. Lewycka, C. Mwansambo, M. Rosato, P. Kazembe, T. Phiri, A. Mganga, H. Chapota, F. Malamba, E. Kainja, M. Newell, G. Greco, A. Pulkki-Brännström, J. Skordis-Worrall, S. Vergnano, D. Osrin and A. Costello, "Effect of women's groups and volunteer peer counselling on rates of mortality, morbidity, and health behaviours in mothers and children in rural Malawi (MaiMwana): a factorial, cluster-randomised controlled trial.," *The Lancet, 381(9879),* pp. 1721-1735, 2013.

[32] National Statistical Office, Malawi, "National Population and Housing Census: Analytical Report," 1998.

[33] G. Reniers and R. Tfaily, "Polygyny and HIV in Malawi.," *Demographic Research, 19(53),* p. 1811, 2008.

[34] T. Takane, "Tobacco and smallholders in Malawi: village case studies in the Mchinji and

For peer review only - http://bmjopen.bmj.com/site/about/guidelines.xhtml

Mangochi Districts.," *African Research Series, 93-119.,* 2005.

[35] WHO Anthro for personal computers, version 3.2.2, *Software for assessing growth and development of the world's children.,* Geneva: WHO, 2011.

[36] D. Filmer and L. Pritchett, "Estimating wealth effects without expenditure data - or tears: An application to educational enrollments in states of India," *Demography,* vol. 38, no. 1, pp. 115-132, 2001.

[37] A. C. Cameron, J. B. Gelbach and D. L. Miller, "Bootstrap-based improvements for inference with clustered errors.," *The Review of Economics and Statistics, 90(3),* pp. 414-427, 2008.

[38] A. Cameron and D. Miller, "A Practitioner's Guide to Cluster-Robust Inference," *Journal of Human Resources,* vol. 50, no. 2, pp. 317-372, 2015.

[39] N. K. Gale, G. Heath, E. Cameron, S. Rashid and S. Redwood, "Using the Framework Method for the Analysis of Qualitative Data in Multi-Disciplinary Health Research," *BMC Medical Research Methodology,* vol. 13:117, 2013.

[40] R. G. Lee and T. Garvin, "Moving from information transfer to information exchange in health and health care.," *Social science & medicine, 56(3),* pp. 449-464, 2003.

[41] R. Sear, "Kin and child survival in rural Malawi," *Human Nature, 19(3),* pp. 277-293, 2008.

For peer review only

For peer review only - http://bmjopen.bmj.com/site/about/guidelines.xhtml

Tables

<u>TABLE 1</u>: Distribution of household and women characteristics in controls and differences with treatment group

	Analysis sample		
	Control group (Mean or proportion)	Difference: treatment - control	p value
**Household characteristics**			
Number of members[a]	5.621	0.114	0.875
Number of sleeping rooms[a]	2.036	0.232	0.034**
Household has electricity?[b]	0.2%	0.0%	0.827
Household has radio?[b]	65.1%	0.4%	0.897
Household has bicycle?[b]	49.9%	1.9%	0.699
Household has motorbike?[b]	0.8%	-0.1%	0.879
Household has paraffin lamp?[b]	93.9%	1.7%	0.815
Household has oxcart?[b]	5.1%	-1.9%	0.198
Agricultural household[b]	100%	-0.2%	0.422
Main flooring material: dirt, sand or dung[b]	92.4%	-1.7%	0.565
Main roofing material: natural material[b]	87.6%	-1.7%	0.697
Piped water[b]	1.5%	2.2%	0.494
Traditional pit toilet[b]	78.3%	4.4%	0.356
Wealth index[a]	-0.087	0.034	0.897
**Woman characteristics**			
Married[b]	71.8%	-4.9%	0.046**
Completed primary education[b]	70.9%	2.8%	0.529
Completed secondary education[b]	7.6%	-2.2%	0.268
Age[a]	24.592	-0.993	0.026**
Chewa[b]	95.4%	-3.9%	0.452
Christian[b]	98.3%	0.5%	0.609
Farmer[b]	70.9%	-4.5%	0.316
Student[b]	16.4%	2.3%	0.380
Small business owner[b]	4.0%	2.1%	0.356
N	411	475	

Notes: Household and mother level characteristics in 2004 corresponding to married main respondent mothers present in the second follow-up survey with children born after the intervention began in July 2005. [a] Continuous variable who which the mean is reported; [b] Binary variable, for which proportions are reported.

*= p<0.1, **=p<0.05, ***=p<0.01. p values are calculated using the wild cluster bootstrap-t procedure outlined in Cameron et al. (2008).

For peer review only - http://bmjopen.bmj.com/site/about/guidelines.xhtml

TABLE 2: Distribution of family networks indicators in controls and differences with treatment group for sampled children.

	Control group	Difference: treatment - control	p value	N
Maternal grandmother[a]	87.3%	1.2%	0.739	2260
Paternal grandmother[a]	80.7%	6.3%	0.132	2252
Mother's sisters[b]	2.835	0.047	0.835	2266
Mother's brothers[b]	2.556	0.207	0.180	2263
Father's sisters[b]	2.336	0.246	0.290	2266
Father's brothers[b]	2.453	0.213	0.288	2267

Notes: [a]Binary variable for which percentages are reported. [b]Discrete, non-binary variables for which mean values are reported. Sample includes all children born since July 2005, who were aged 0-53 months at the time of interview, and whose mothers were married main respondents to the follow up surveys in 2008-09 and 2009-10. A pooled dataset from both follow-up surveys is used to construct means.

For peer review only

Table 3: Relationship between baseline characteristics and family network size, with p-values

Panel A: Household characteristics

	Number of members	Number of rooms	wealth Index
Maternal grandmother alive (0/1)	0.027	-0.04	0.256**
SE	(0.307)	(0.103)	(0.114)
Cluster wild bootstrap t p-value	{0.929}	{0.779}	{0.032}
Paternal grandmother alive (0/1)	-0.209	-0.02	-0.129
SE	(0.276)	(0.094)	(0.186)
Cluster wild bootstrap t p-value	{0.478}	{0.919}	{0.549}
Parents siblings alive	0.025	0.01	0.016
SE	(0.037)	(0.011)	(0.011)
Cluster wild bootstrap t p-value	{0.627}	{0.400}	{0.144}
N	881	879	881

Panel B: Mother characteristics

	Primary education	Secondary education	Age	Chewa	Christian	Farmer	Student	Small business owner
Maternal grandmother alive	0.124*	-0.028	-4.463***	0.01	-0.007	-0.111*	0.150***	-0.046
SE	(0.057)	(0.033)	(0.687)	(0.039)	(0.010)	(0.054)	(0.033)	(0.029)
Cluster wild bootstrap t p-value	{0.056}	{0.396}	{0.002}	{0.863}	{0.657}	{0.056}	{0.002}	{0.160}
Paternal grandmother alive	0.071**	-0.004	-2.845***	0.005	0.009	-0.01	0.04	-0.024
SE	(0.032)	(0.029)	(0.563)	(0.023)	(0.012)	(0.036)	(0.024)	(0.027)
Cluster wild bootstrap t p-value	{0.028}	{0.871}	{0.002}	{0.853}	{0.569}	{0.739}	{0.104}	{0.370}
Parents siblings alive	-0.004	0	0.044	0.007	0	0.012**	-0.011***	-0.003*
SE	(0.004)	(0.002)	(0.069)	(0.005)	(0.001)	(0.004)	(0.003)	(0.001)
Cluster wild bootstrap t p-value	{0.346}	{0.925}	{0.537}	{0.228}	{0.462}	{0.024}	{0.004}	{0.074}
N	881	881	881	881	881	881	881	881

Notes: OLS regressions of with baseline characteristics gathered in 2004 as the dependent variable and family networks as independent variables. Sample contains married main respondent mothers present in the second follow-up survey with children born after the intervention began in July 2005.. Standard errors computed using the cluster correlated Huber-White estimator are reported in parentheses and p-values in curly brackets. P values are calculated using the wild cluster bootstrap-t procedure outlined in Cameron et al. (2008). The wealth index was calculated using principal components analysis as recommended by [36].

*= p<0.1, **=p<0.05, ***=p<0.01

For peer review only - http://bmjopen.bmj.com/site/about/guidelines.xhtml

TABLE 4: Estimated effects on height-for-age z-scores with 95% confidence intervals from three linear regression models

	Model 1		Model 2		Model 3	
	Coefficient	95% Confidence Interval	Coefficient	95% Confidence Interval	Coefficient	95% Confidence Interval
Treatment	0.296***	[0.116, 0.484]	0.441	[-0.335, 1.028]	0.467	[-0.344, 1.050]
Maternal grandmother			-0.265**	[-0.528, -0.021]	-0.259**	[-0.503, -0.019]
Maternal grandmother*T			0.168	[-0.296, 0.615]	0.145	[-0.324, 0.575]
Paternal grandmother			0.008	[-0.200, 0.236]	0.006	[-0.192, 0.238]
Paternal grandmother*T			-0.253*	[-0.529, 0.029]	-0.235*	[-0.493, 0.039]
Mothers sisters			0.031	[-0.052, 0.103]		
Mothers sisters*T			-0.056	[-0.163, 0.057]		
Fathers sisters			0.002	[-0.140, 0.140]		
Fathers sisters*T			0.042	[-0.110, 0.188]		
Mothers brothers			-0.001	[-0.116, 0.082]		
Mothers brothers*T			0.024	[-0.109,0.170]		
Fathers brothers			0.016	[-0.064, 0.115]		
Fathers brothers*T			-0.034	[-0.148, 0.075]		
Total siblings of mother					0.016	[-0.032, 0.062]
Total siblings of mother*T					-0.019	[-0.069, 0.037]
Total siblings of father					0.01	[-0.038, 0.054]
Total siblings of father*T					0.001	[-0.080, 0.072]
R-Squared	0.19		0.195		0.193	
N	2017		2017		2017	

Table notes: OLS regressions with height for age (HAZ) scores as dependent variable. Model 1 estimates the overall effect of exposure to the program. Models 2 and 3 estimate regressions that allow the program effect to vary with different extended family members. Inference is conducted using the wild cluster bootstrap-t procedure recommended by Cameron et al (2008). 95% confidence intervals calculated according to the method recommended in Cameron and Miller (2015).

All regressions include the following controls: Cluster level controls: Education and Chewa ethnicity in 2004, Household level controls: A wealth index calculated in 2004, Mother level controls: Whether she had completed primary school, was working as a farmer or was a student in 2004, current age, age^2 and logarithmic height. Child level controls: Month of measurement, age, age^2, gender, number of older siblings, number of older $siblings^2$.

Sample includes all children born after the intervention start date in July 2005 to married main respondent mothers, who were aged 0-53 months at the time of measurement. Column 1 indicates the effect of intervention assignment on HAZ scores, for the sample where family networks information is not missing. Models 2-3 indicate how intervention effects on HAZ scores vary with the presence of different extended family members. *= p<0.1, **=p<0.05, ***=p<0.01

For peer review only

For peer review only - http://bmjopen.bmj.com/site/about/guidelines.xhtml

Appendix 1

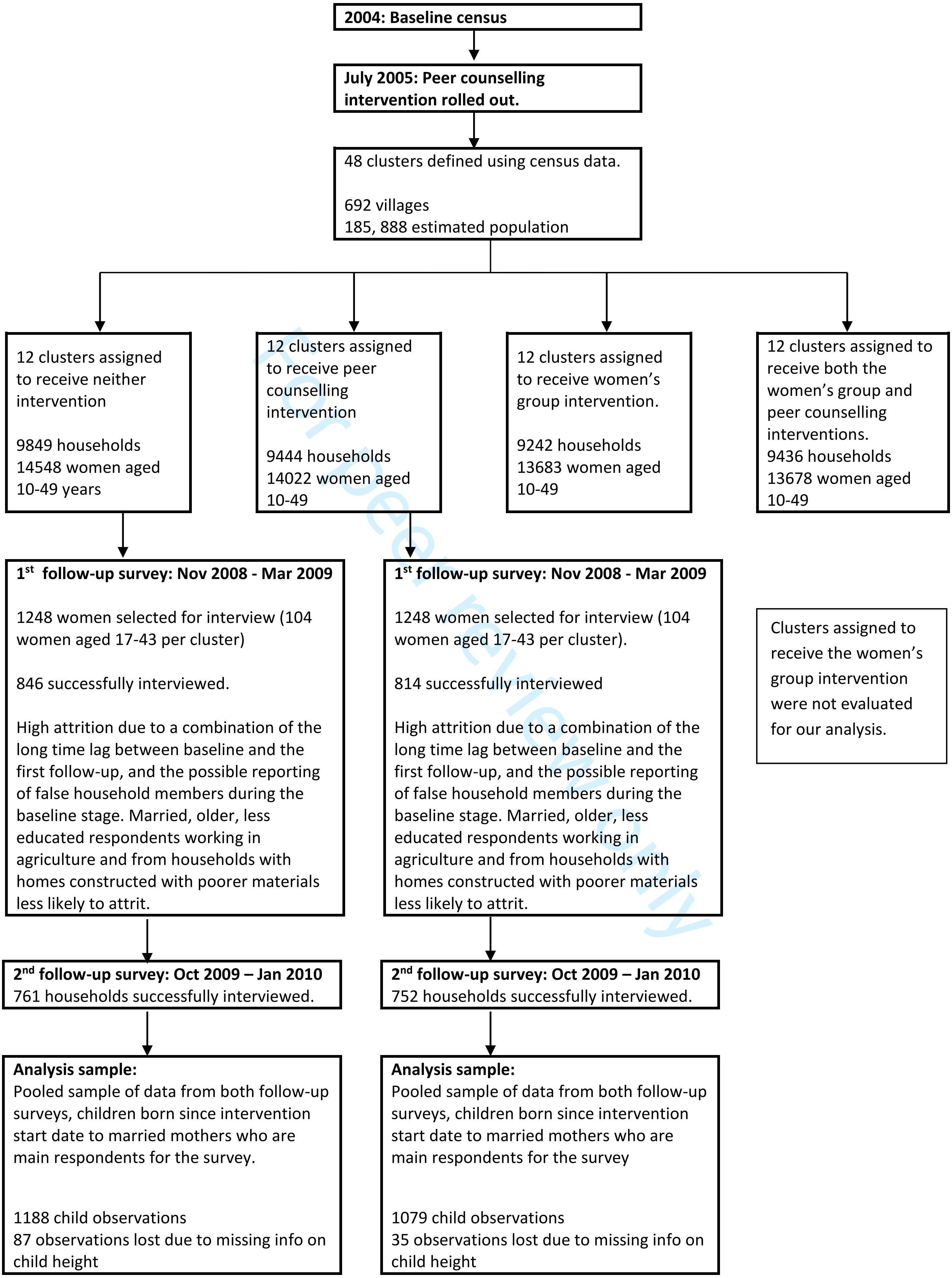

For peer review only

STROBE Statement—Checklist of items that should be included in reports of ***case-control studies***

	Item No	Recommendation	Complete?
**Title and abstract**	1	(*a*) Indicate the study's design with a commonly used term in the title or the abstract	✓
		(*b*) Provide in the abstract an informative and balanced summary of what was done and what was found	✓ (pg 2)
**Introduction**			
Background/rationale	2	Explain the scientific background and rationale for the investigation being reported	✓ (pg 4-5)
Objectives	3	State specific objectives, including any prespecified hypotheses	✓ (pg 6)
**Methods**			
Study design	4	Present key elements of study design early in the paper	✓ (pg 6 – 7)
Setting	5	Describe the setting, locations, and relevant dates, including periods of recruitment, exposure, follow-up, and data collection	✓ (pg 6 - 9, 12 - 13)
Participants	6	(*a*) Give the eligibility criteria, and the sources and methods of case ascertainment and control selection. Give the rationale for the choice of cases and controls	✓ (pg 6-9)
		(*b*) For matched studies, give matching criteria and the number of controls per case	
Variables	7	Clearly define all outcomes, exposures, predictors, potential confounders, and effect modifiers. Give diagnostic criteria, if applicable	✓ (pg 8-9)
Data sources/ measurement	8*	For each variable of interest, give sources of data and details of methods of assessment (measurement). Describe comparability of assessment methods if there is more than one group	✓ (pg 8-9)
Bias	9	Describe any efforts to address potential sources of bias	✓ (pg 9-12)
Study size	10	Explain how the study size was arrived at	
Quantitative variables	11	Explain how quantitative variables were handled in the analyses. If applicable, describe which groupings were chosen and why	✓ (pg 8-9)
Statistical methods	12	(*a*) Describe all statistical methods, including those used to control for confounding	✓ (pg 9-12)
		(*b*) Describe any methods used to examine subgroups and interactions	✓ (pg 9-12)
		(*c*) Explain how missing data were addressed	✓ (pg 8-9)
		(*d*) If applicable, explain how matching of cases and controls was addressed	
		(*e*) Describe any sensitivity analyses	✓ (pg 9-12)
**Results**			
Participants	13*	(a) Report numbers of individuals at each stage of study—eg numbers potentially eligible, examined for eligibility, confirmed eligible, included in the study, completing follow-up, and analysed	✓ (Appendix 1)
		(b) Give reasons for non-participation at each stage	✓ (Appendix 1)
		(c) Consider use of a flow diagram	✓ (Appendix 1)
Descriptive data	14*	(a) Give characteristics of study participants (eg demographic, clinical,	✓ (Table 1)

For peer review only

For peer review only - http://bmjopen.bmj.com/site/about/guidelines.xhtml

		social) and information on exposures and potential confounders	
		(b) Indicate number of participants with missing data for each variable of interest	✓ (Appendix 1)
Outcome data	15*	Report numbers in each exposure category, or summary measures of exposure	✓ (Table 1)
Main results	16	(*a*) Give unadjusted estimates and, if applicable, confounder-adjusted estimates and their precision (eg, 95% confidence interval). Make clear which confounders were adjusted for and why they were included	✓ (Table 4)
		(*b*) Report category boundaries when continuous variables were categorized	✓
		(*c*) If relevant, consider translating estimates of relative risk into absolute risk for a meaningful time period	
Other analyses	17	Report other analyses done—eg analyses of subgroups and interactions, and sensitivity analyses	✓ (Table 4)
**Discussion**			
Key results	18	Summarise key results with reference to study objectives	✓ (pg 14 - 16)
Limitations	19	Discuss limitations of the study, taking into account sources of potential bias or imprecision. Discuss both direction and magnitude of any potential bias	✓ (pg 19)
Interpretation	20	Give a cautious overall interpretation of results considering objectives, limitations, multiplicity of analyses, results from similar studies, and other relevant evidence	✓ (pg 16 – 19)
Generalisability	21	Discuss the generalisability (external validity) of the study results	✓ (pg 16-19)
**Other information**			
Funding	22	Give the source of funding and the role of the funders for the present study and, if applicable, for the original study on which the present article is based	✓ (pg 20)

*Give information separately for cases and controls.

Note: An Explanation and Elaboration article discusses each checklist item and gives methodological background and published examples of transparent reporting. The STROBE checklist is best used in conjunction with this article (freely available on the Web sites of PLoS Medicine at http://www.plosmedicine.org/, Annals of Internal Medicine at http://www.annals.org/, and Epidemiology at http://www.epidem.com/). Information on the STROBE Initiative is available at http://www.strobe-statement.org.

For peer review only
