# Supplementary material for: Family networks and infant health promotion: a mixed-methods evaluation from a cluster randomised controlled trial in rural Malawi
Source: BMJ Open. 2018 Jun 7;8(6):e019380. doi: 10.1136/bmjopen-2017-019380 (PMC6009513; doi:10.1136/bmjopen-2017-019380)
Supplement: Reviewer comments [file bmjopen-2017-019380.reviewer_comments.pdf]

PEER REVIEW HISTORY

BMJ Open publishes all reviews undertaken for accepted manuscripts. Reviewers are asked to complete a checklist review form (**http://bmjopen.bmj.com/site/about/resources/checklist.pdf**) and are provided with free text boxes to elaborate on their assessment. These free text comments are reproduced below.

ARTICLE DETAILS

**TITLE (PROVISIONAL)**	Family networks and infant health promotion: a mixed-methods evaluation from a cluster randomised controlled trial in rural Malawi
**AUTHORS**	Scott, Molly; Malde, Bansi; King, C.; Phiri, Tambosi; Chapota, Hilda; Kainja, Esther; Banda, Florida; Vera-Hernandez, Marcos

VERSION 1 – REVIEW

**REVIEWER**	Helen Nabwera<br>Liverpool School of Tropical Medicine, UK
**REVIEW RETURNED**	19-Sep-2017

**GENERAL COMMENTS**	Overall comments<br><br>This study addresses a very important child health problem and the findings will be useful in developing interventions at the community level. The aims of the study are clearly stated. However, until we get the "Discussion", the authors do not outline how the quantitative and qualitative findings are integrated and the two methods therefore appear to be 2 different studies with a time interval of 5 years. It would be useful to outline how these methods were integrated at the "Methods" and "Results" sections. There are a few other areas that need to be addressed to strengthen this paper.<br>Major<br><br>Abstract<br>Page 2, Line 14: Methods: Please describe this accurately as has been stated in the "Methods" section in the main text. i.e. that this was not the primary study.<br>Page 2, Line 36: Results: "However the effect size was between 0.235 (p=0.088) and<br>0.253 (p=0.058) standard deviations lower if the paternal grandmother is still alive."<br>- Is this a 95% confidence interval. It is probably better to present effect size estimates as a confidence interval.<br>Page 2, Line 51: Conclusion should also reflect the important quantitative findings.<br>Linear multivariate regressions test whether the impact of the intervention, which was<br>implemented as a cluster randomised control trial, is influenced by different extended<br>family members<br>Page 3, Line 10: Strengths and limitations<br>• Linear multivariate regressions test whether the impact of the intervention, which was<br>implemented as a cluster randomised control trial, is influenced by different extended

	family members<br>• Quantitative analysis allows different extended family members – paternal grandmothers,<br>maternal grandmothers, among others – to have different impacts on the program's<br>success<br>I would suggest that these 2 points should be merged into one.<br><br>Introduction<br>Page 4, line 6: Child health outcomes are influenced by individuals besides the mother and father, with a rich literature devoted to the contribution of extended family members. Please provide some references for this statement.<br><br>Results<br>Page 12, line 22: However, the effect of the intervention on HAZ scores is between 0.235 (p=0.058) and 0.253 (p=0.088) SD smaller for children whose paternal grandmother is alive. The results suggest that maternal grandmothers are associated with lower child health outcomes in the absence of the intervention. Is it the involvement of the paternal or maternal grandmother that is associated with poor outcomes? Please clarify.<br>Page 12, line 54: After this and all the others quotes, please provide more information about respondent to show that it is not the same respondent with a group that is sharing this information.<br><br>Discussion<br>Page 16, in the section on limitations, please discuss the challenges of integrating data that was collected 5 years apart.<br><br>Tables<br>Page 26: Table 4, consider presenting the effect size of intervention of HAZ as a 95% confidence interval. Also, please consider revising how you have presented the information on this table as it is not very clear. Columns 4-6 appear to be missing.<br><br>Minor<br>Page 16, line 10: Fitzsimons et al (2016) Please provide reference this in line with agreed format.

**REVIEWER**	Alison Talbert<br>Centre for Geographic Medicine Research (Coast)<br>Kenya
**REVIEW RETURNED**	12-Oct-2017

**GENERAL COMMENTS**	The paper looks at the effect of a health promotion intervention delivered to mothers on child growth and how its impact is modified by the presence of living relatives such as grandmothers and parents' siblings. The subject is of interest to those working in public health in Africa and other LMICs where extended families are prevalent. The author presents a secondary analysis of data collected from surveys of families in 2008 -2010 to look at the effects of a peer counselling intervention running from 2004 (to present day?) to mothers of infants under 6 months on child growth, and a new qualitative study on the impact of extended family members that was done in 2015.<br><br>Unfortunately the results are presented in such a way that it is confusing for the reader and I feel that a major revision of the tables is needed, preferably with a statistician's input.

	Abstract.<br>1. It should be clearly stated here that this is a secondary data analysis of a previous RCT with an additional qualitative study.<br>2. Line 42 - change "reduction in height" to "reduction in height-for age z score"<br><br>Introduction<br>There is no mention of the normal extended family living arrangements for this population. It is important to describe if they are patrilineal and/or patrilocal because this might explain the greater impact of the paternal grandmother if she has a high status and is a resident member of the household rather than the maternal grandmother who would be living elsewhere.<br><br>Strengths and limitations<br>Line 16 needs rephrasing, I suggest: "quantitative analysis allows estimation of the size of effect different extended family members have on the impact..."<br><br>Quantitative analysis<br>Page 7 line 6 At what ages was height of the children measured and by whom? How were Z scores calculated (WHO MGRS?)<br>Page 7 line 41 "Balance was preserved" - wording needs changing as it suggests an active process of balancing. Need to describe the baseline characteristics of the 2 groups as being similar<br>Page 9 line 24. I think that the analysis would have been improved if the model tested the impact of a grandmother or other family member who lived in the same household or nearby. In this case the grandmother would be more likely to give advice than one who was alive but not likely to be in face-to-face contact. If you have data on this you should test the model including this variable.<br><br><br>Qualitative analysis<br>Page 10 line The FGDs with peer counsellors and others were conducted a long time after the intervention started so there might be recall bias. Also the peer counsellors had a high attrition rate which might affect the findings.<br>Page 10 line 18 purposively is the more common term<br>Page 13 line 48 Language is confusing - it reads as if breastfeeding, weaning and complementary feeding messages were not the focus (when they were) when you mean the extended members were not the target of the messages<br>References<br>Page 19 line 6 The first reference needs information on name of publishers<br><br>Tables need to be presented differently - I suggest an expert statistical review. Layout is confusing for the reader and numbers are not explained clearly - some are proportions, others averages and others regression coefficients.<br>Table 1 Change the title to something like Household and mother's characteristics at baseline survey<br>Table 1 Only the control group's characteristics are shown but both should be shown. No need for p values<br>Table 1 it is not clear what the numbers in the 1st column represent - some are averages others are proportions. For the characteristics better to put e.g. how many households in each group have electricity (n) then percentages

	Table 1 - maybe put in a row for the composite wealth index which is mentioned in table 3<br>Table 2 - as in table 1 show the results for each group (control and intervention) in separate columns rather than only for control and second column showing difference from control. No need for p values for baseline characteristics.<br>Table 2 - better to label numbers as n and percentages for grandmothers, and others as average numbers.<br>Table 3 - needs laying out differently. Give 95% confidence intervals for the regression coefficients rather than standard errors and p values<br>Table 4 needs major revision. What do the column headings mean? 1 seems to be crude estimate and 2 and 3 are adjusted estimates according to different models but the rationale for using the different models is not explained in the methods section of the paper<br>Table 4 notes: it mentions columns 2-6 but there are only 3 columns in the table. This looks like copy and pasted notes from elsewhere.<br>Appendix 1 Number of households interviewed in the peer counselling clusters follow up survey of Oct 2009 -Jan 2010 differs from that in Fitzsimon's paper (753 vs 752)

**REVIEWER**	Peter Rohloff<br>Brigham and Women's Hospital (USA)<br>Maya Health Alliance (Guatemala)
**REVIEW RETURNED**	12-Oct-2017

**GENERAL COMMENTS**	I really enjoyed reading this important study an a topic that often goes neglected - the impact of extended family members on behavior change interventions for improving child growth.<br><br>Abstract<br>-I would recommend reporting confidence intervals rather than p values in the abstract.<br><br><br>Strengths/Limitations boxes are bit generic, and generally just repeat text already evident from the abstract. Would be explicit about limitations here, and also use these bullets as an opportunity to make policy or programming points and really emphasize the importance of the findings<br><br>Introduction:<br><br>Theoretical framework in the introduction could use a bit of refining. This is especially evident in the paragraph starting on page 4, line 47 - this brief literature review brings together citations from what really comprise a quite wide range of different kinds of programs and strategies (e.g, home based one on one caregiver counseling vs primary school programs). I'd suggest focusing the introduction more narrowly on just on the type of behavior change/education strategies that are relevant to the described intervention (e.g., interventions for caregivers of young children), and then describe in a bit more detail how evidence for efficacy within that narrower group is "mixed"<br><br>Page 5, Line 8 - this is true, there isn't a lot of literature on this topic, but there is some - including several studies that the authors cite a few paragraphs earlier. I'd expand this section a bit and engage what literature is available, so that readers have a better sense of the state of the field. This is especially important, because the author's work here involves a really large data set and therefore has

	something very important to add to what amount to group of smaller, mostly qualitative studies.<br><br>Methods:<br><br>A few details of the qualitative approaches could be clarified. “All translations agreed between the researchers” - does this mean that transcripts were initially into Chichewa and then translated into English? How was agreement determined? Were the FDG facilitators different individuals than the authors here who conducted the transcribing and coding? If coding was conducted independently by at least two authors, does this mean that all transcripts were double-coded and are values for inter-coder reliability available? If not, and instead authors worked together to achieve complete coding consensus, then what procedures were used to identify coding discrepancies and resolve differences?<br><br>In the methods (and also results) the pooling procedure used for data raises a few points for me that could be clarified. Most generally, what was the rational for two followup surveys? Was the plan to analyze certain variables longitudinally, or was this just an effort to capture data missed on the first round? I ask in part because in Tables 2-3 it says only the second survey was used. Does this mean that no data was missing or that data from the first survey wasn’t used to complement the second? If data was pooled in some way then obvious that raises other questions, such as two conflicting answers to the same question by one subject between the two surveys, or family members dying between survey points.<br><br>Results:<br><br>Throughout the results section and Tables I would find reporting of confidence intervals more informative than P values, and this would also be in line with more typically writing practices for randomized trials. Adding standard deviations to continuous variables (or IQR and media of not parametric) would also aid the reader. This is not a major point however and I would defer to the editors.<br><br>Table 1- the description about the sample containing children born after 2005, second survey, etc i s not pertinent to this table which contains only the caregiver and household level data from the 2004 baseline, correct?<br><br>Table 2: The 0/1 indicates that for grandparents, this is the proportion of families with a living grandparent? This is proportion vs means for the other variables in the tables?<br><br>Appendix 1 - drop off at followup survey. I understand that randomization was preserved (Table 1) despite drop off, but it would also be very interesting to see characteristics of subjects/families who dropped off vs those who were successfully interviewed (perhaps in the Appendix)<br><br>For Table 3, the line about restricted data to one eligible child is not pertinent, correct, because this is again all caregiver/household level data? Can the wealth index reference be added to the manuscript?<br><br>For Table 4 (and corresponding methods) I have the same question about how the data from the surveys was pooled. HAZ tends to

	decline most around 2-3 yrs and then recover some. So, were children potentially measured twice and, if so, which HAZ was used. Also for this Table and corresponding text, it would be nice to see estimates of each model's performance (e.g R2)<br><br>Qualitative results: I kept hoping for some data that would help differentiate between maternal and paternal grandmothers, and I assume that such data was not available to the team?<br><br>Conclusions:<br><br>- Some additional context on the study sites, and local cultural practices might also help to explain or better interpret the results. I find myself wanting much more explanation (potentially even speculation) from the authors about this differential impact. Why do paternal (and not maternal grandmother's command so much more influence? In cultures I'm more familiar with (in Mexico and Central America) we see exactly the same thing, and this is because new families usually take up patrilocal residence with the father's parents - hence the grandmother who is available and constantly present to exert influence is the paternal grandmother. Some details of this nature would be really helpful<br><br>- Also it would be nice to have some interpretation of the findings from the regression analysis of the negative impact of maternal grandmother presence (independent of the intervention) on growth, since this is the other major element of the regression analysis.

**REVIEWER**	Dr Janet Smylie<br>St. Michael's Hospital and University of Toronto, Canada
**REVIEW RETURNED**	26-Oct-2017

**GENERAL COMMENTS**	Major concerns:<br>1. lack of clarity with respect to what is meant by "traditional" practices and assumption that these practices undermine interventions. If the authors are referring to pre-colonial health practices, these are extremely complex and interwoven with colonial influences<br>2. Qualitative methods lack of specification of theoretical frame, analytic lens, and social location of research team who did the analysis - given the cross-cultural context this is essential<br>3. brevity of presentation of qualitative findings - does not allow for an assessment of whether or not saturation appears to have been reached, lacks contrasting findings, makes it hard to understand context of presented findings and assess truthworthiness of findings<br>4. the interpretation of the qualitative findings seem to be hinged on the use of "traditional" views on childrearing by grandparents interfering with "standard practice" however the results on "traditional practices" only cite on example and state that these practices are uncommon or hidden<br>Minor concerns:<br>Quantitative methods appear sound but should be reviewed by a statistician - seems like underpowering may have been an issue

**REVIEWER**	Francesco Sera<br>Department of Social & Environmental Health Research (SEHR)<br>Faculty of Public Health and Policy<br>London School of Hygiene and Tropical Medicine
**REVIEW RETURNED**	21-Nov-2017

**GENERAL COMMENTS**	This paper present the analysis of a cluster randomized trial examining how family network modify the outcome of an infant health promotion in rural Malawi.<br>The paper is clear and well structured, the analysis plan is coherent with the study design and with the aim of the study. In particular the interaction between the family network and the treatment is evaluated at individual level through a general linear model with an interaction term between the dummy variable representing the treatment and the indicators for the family network. Given the small number of clusters, the authors have used the cluster bootstrap method (Cameron et al 2008) to take into account possible non-independence of the outcome within the clusters.<br>I have only some doubt on the models that evaluate the relationship between some covariates (household and mother characteristics) and the possible modifiers (family network indicators). Some covariates are categorical or even dichotomous and these variables have been considered as outcome in OLS models with family network indicators as possibly explanatory variables. It would have been more appropriate using logistic or polytomous logistic regression models for this analysis.<br>Tables can be improved.<br>Table 1. The title should be changed in something like “distribution of household and women characteristics in controls and differences with treatment group”. For categorical variables the percentage should be reported for absolute values and differences.<br>Table 2. Also here the title should be changed in “distribution of family networks indicators in controls and differences with treatment group”. For dummy variables the percentage should be reported for absolute values and differences. There is no need to report the coding (0/1).<br>Table 3. The numbers in brackets should be indexed as SE and p values in the rows. In panel B the symbol “#” need to be replaced by “Numbers of”.<br>Table 4. The title need to be changed. Something like “Coefficients and (SE) of the multivariate linear models evaluating the interaction between treatment and family network indicators”. The number in brackets should be indexed as (SE), perhaps building two columns one for the coefficient and one for the SE. (1) (2) and (3) should be replaced by Model 1, Model 2 and Model 3.

**REVIEWER**	Scott R Walter<br>Macquarie University
**REVIEW RETURNED**	04-Dec-2017

**GENERAL COMMENTS**	The following comments relate only to the statistical methods used in this paper. In general, the proposed model seems appropriate to answer the main research question and the sampling strategy seems sound. However, there are some areas where clarity could be improved. There are also issues with model interpretation that have implications for the study’s conclusions.<br><br>1. In the results section of the abstract the authors make the claim that there was “no effect of parents’ siblings.” A non-significant result does not indicate that there is ‘no effect’, but rather that there is ‘no evidence of an effect’. This may seem like a small distinction, but the former claims something concrete about an effect (that there is none), whereas the latter indicates a lack of evidence on which to make a claim.

	2. Methods: Model Specification and Estimation<br>The description of the model would be enhanced by more explicit description of how the model relates to the research question. Specifically, that the interaction terms assess whether certain family members modify the intervention effect. A positive (or negative) negative significant interaction indicates a greater change in HAZ score than would be expected due to the intervention alone and provides evidence that the intervention effect is enhanced (or diminished) in the presence of that particular family member.<br><br>3. Results: Table 3 displays an extensive set of results derived from analyses that appear non-trivial. Where the methods are sketched out in a footnote of the table, perhaps it makes more sense to describe the methods in the methods section itself and provide a little more detail. This would also allow space to give some justification for this part of the analysis and how it supports the main research question. As it is now, the results Table 3 pop up a little unexpectedly and its relevance only becomes clear after some scrutiny by the reader.<br><br>4. Results: Table 4 represents the main findings relative to the study aim, but would benefit from some additional labelling to make it easier to interpret.<br>a. At first it wasn't obvious what the three columns of results represented. I could work out that 1 was a crude model, and 2 and 3 were adjusted models with parent siblings in different formats but some kind of column headings would help to make this more immediately apparent. In retrospect I saw that this had been described in the methods but the description hadn't made sense until I got to Table 4 and went back to the methods specifically looking for it. So clarifying the three models in the methods would be a big help too. The fact that the equation comes first in the methods gives the impression that there is one model, then the language used to describe the three models doesn't do much to change that first impression. Perhaps something like this would be more explicit: "We fitted three models, one crude model with the intervention term only, and two full models as specified in the equation each treating parent siblings differently…"<br>b. It was not immediately apparent that the numbers in brackets were p-values. It is mentioned in the footnote, but it might be clearer to put it in the title, e.g. "Table 4. Estimated height-for-age scores {with p-values} from three linear regression models".<br><br>5. The interpretation of the model estimates seems to be the key to addressing the question of whether family members influence (i.e. are associated with) the intervention efficacy, so it is important to get this right.<br><br>a. Results: In paragraph 4 of the Results, there is an initial reporting of the overall intervention effect (from the crude model) of 0.296, which all seems fine. Then later in the paragraph there is a claim that the intervention effect is 0.235-0.253 which doesn't make sense as these are the estimates for the interaction term 'paternal grandmother*T'. First order terms, e.g. 'maternal grandmother', indicate the pre-intervention difference in HAZ score between settings where the is the MGm versus settings where there is not. So in Table 4, having the MGm present pre-intervention is associated with reduced child height compared to families with no MGm present (using model 2: difference in HAZ = -0.265, p=0.05) .

	For the paternal grandmother, however, there is no evidence of a difference if the PGm is present or not pre-intervention (difference in HAZ = 0.008, p=0.99).<br>For the interaction terms, e.g. 'MGm*T', this indicates changes over time where a family member is present in addition to the intervention effect. So where the MGm was not present, HAZ was associated with an increase of 0.441 with the intervention. When the MGm was present this increase in HAZ was augmented by 0.168, resulting in an increase of 0.441+0.168=0.609. In contrast, when the PGm was present the intervention effect was reduced by -0.253 resulting in a smaller increase of 0.441-0.253=0.188 with the intervention.<br>Having spelled out how the interaction terms are interpreted in the methods (comment 2), this kind of reporting of results will hopefully be easier for readers to follow.<br><br>b. Some of the issues in the comment above carry over into the discussion where the model results are interpreted and discussed. Currently the text claims that grandmothers can be a barrier to the intervention and behaviour change. This is not quite what the results show. Rather the main findings seem to be:<br>i. Prior to the intervention, maternal grandmothers were associated with lower HAZ scores. It is important not to overstate the direction of causality here. It may not mean that the maternal grandmothers were causing the children to be undersized. Could it be that grandmothers are more likely to be present when children are undersized in the sense of offering support? Or are there other possible explanations? In contrast paternal grandmothers did not seem to be associated with child size pre-intervention, and the authors may have some plausible explanation for this.<br>ii. The intervention-related increase in HAZ score was amplified in the presence of maternal grandmothers but reduced in the presence of paternal grandmothers. This suggests only paternal grandmothers may be a barrier to the intervention, while maternal grandmothers increased its success. In revising the discussion, it will be important to address potential reasons for the differential effects on the intervention between maternal and paternal grandmothers (the 'in-law' effect?!) in a way that is consistent with interpretation of the differential pre-intervention results mentioned in 5.b.i.<br>iii. There was no evidence of an association with HAZ score for other family members, either pre or post intervention. This lack of evidence bolsters the case for focusing on inclusion of grandmothers in the design of interventions as discussed in paragraphs 4 and 5 of the discussion.<br><br>In light of this, particularly the differential pre and post effects of grandmothers, much of the discussion needs reworking to more closely align with the results of the model.

VERSION 1 – AUTHOR RESPONSE

Many thanks for your encouraging comments, and for the time you have taken to read the paper. Your comments have been extremely useful in improving the paper.

In what follows, we provide a detailed reply to each comment, one by one. We reproduce the original comments and provide our response and reproduce new text from the revised version of the paper beneath each comment. We hope this is clear but do please come back to us if anything is unclear.

Reviewer 1: Helen Nabwera
This study addresses a very important child health problem and the findings will be useful in developing interventions at the community level. The aims of the study are clearly stated. However, until we get the “Discussion”, the authors do not outline how the quantitative and qualitative findings are integrated and the two methods therefore appear to be 2 different studies with a time interval of 5 years. It would be useful to outline how these methods were integrated at the “Methods” and “Results” sections. There are a few other areas that need to be addressed to strengthen this paper.
We thank the reviewer for this comment, and agree that the mixed-methods approach could be presented in a more integrated way. We have added some text in the methods section to explain more how we integrated the two datasets. The new text is (p.6):
"The quantitative data was collected and analysed first, and used to design the qualitative aspect of the study. The overall interpretation of our findings was integrated following an initial analysis of the qualitative data."

Page 2, Line 14: Methods: Please describe this accurately as has been stated in the “Methods” section in the main text. i.e. that this was not the primary study.
We have changed the text to make it clear that this is a secondary, sequential mixed-methods study.

Page 2, Line 36: Results: “However the effect size was between 0.235 (p=0.088) and 0.253 (p=0.058) standard deviations lower if the paternal grandmother is still alive.” - Is this a 95% confidence interval. It is probably better to present effect size estimates as a confidence interval.
Since the study has a small number of clusters (24), we use a wild cluster bootstrap-t procedure recommended by Cameron, Gelbach and Miller (2008) for inference in this paper. This method provides us with a p-value, which we report in the draft. To calculate the associated confidence intervals, we apply a method suggested by Cameron and Miller (2015). This method relies on simulations, and is thus very computationally intensive, and also requires a number of adjustments to the simulation parameters. Moreover, this method is not available in standard statistical packages, and so we have had to write an ad-hoc program to compute the confidence intervals. Given that this is very computationally intensive (it has taken us around 2 days to obtain the confidence intervals for Table 4, once the program was written), we hope that it’s fine that we have only calculated these for Table 4 only.
As a separate point, Cameron and Miller (2015) indicate that the confidence intervals will usually be asymmetric, which is what we find in our case as well.

Page 2, Line 51: Conclusion should also reflect the important quantitative findings.
We have amended the conclusion accordingly.

Page 3, Line 10: Strengths and limitations
•Linear multivariate regressions test whether the impact of the intervention, which was implemented as a cluster randomised control trial, is influenced by different extended family members
• Quantitative analysis allows different extended family members – paternal grandmothers, maternal grandmothers, among others – to have different impacts on the program’s success I would suggest that these 2 points should be merged into one.
We have followed the reviewer’s suggestion and merged the two points. The new text is:
Quantitative analysis, using linear multivariate regression, allows estimation of the size of effect different extended family members – including paternal and maternal grandmothers – have on the impact of the intervention

Page 4, line 6: Child health outcomes are influenced by individuals besides the mother and father, with a rich literature devoted to the contribution of extended family members. Please provide some references for this statement.

We have now included some references, which were reported further down in the paragraph in the earlier draft, to support this statement.

Page 12, line 22: However, the effect of the intervention on HAZ scores is between 0.235 (p=0.058) and 0.253 (p=0.088) SD smaller for children whose paternal grandmother is alive. The results suggest that maternal grandmothers are associated with lower child health outcomes in the absence of the intervention. Is it the involvement of the paternal or maternal grandmother that is associated with poor outcomes? Please clarify.
We recognise now that our explanation of the results was not as clear as it could have been. In particular, the sentences above were commenting on two different results obtained in the quantitative analysis. In particular, we find a negative coefficient on the 'Maternal Grandmother Alive' coefficient and on the interaction term 'Paternal Grandmother Alive*Tj'. The former indicates a negative association between living maternal grandmothers and child HAZ scores independent of the intervention. This cannot, however, be interpreted as a causal effect due to the presence of confounders (e.g. increased competition for resources among families with living maternal grandmothers in matrilineal societies). The negative coefficient on the interaction term 'Paternal Grandmother Alive*Tj' term indicates that living paternal grandmothers diminishes the effect of the intervention on child HAZ scores. We have now expanded the explanation of the methodology and results, which we hope clarifies the quantitative findings.

We had hoped to obtain explanation for the different effects of paternal and maternal grandmothers through the qualitative study. However, despite probing questions on any potential differences, participants did not discuss or acknowledge any differences in the roles or influence between parental and maternal grandmothers. Moreover, as the manuscript is already lengthy, hypothesizing about the maternal/paternal nuance was deemed to require too much additional explanation.

Page 12, line 54: After this and all the others quotes, please provide more information about respondent to show that it is not the same respondent with a group that is sharing this information.
We have added participant numbers to the quotes throughout the text.

Discussion
Page 16, in the section on limitations, please discuss the challenges of integrating data that was collected 5 years apart.
This is a good point, and we have now added text to the limitations to include this. While the time lag is likely to mean that some cultural beliefs or behaviours around infant feeding may be different between the context of the quantitative and qualitative data collection, the themes which emerged as important were related to household structure, decision making, and resource allocation. These are likely to be relatively stable over a 5-year period in a rural low-income setting which has had little development over that time period.
New text (p.19):
"Finally, the qualitative and quantitative mixed data were collected sequentially rather than concurrently, making interpretation more challenging with the qualitative data collection conducted five years after the quantitative survey. This may have resulted in recall bias in the qualitative data, and the culture and behaviours around infant feeding may have shifted between the two study phases. This is somewhat supported by the FGDs and interviews from control areas being exposed to the peer counsellors and their messages, posing a challenge to integrating the results. However, as Tthe qualitative data was planned to provide a more in-depth understanding and triangulation of the quantitative findings, rather than drawing conclusions comment on causality, therefore we do not feel this detracts from the our interpretation."

Page 26: Table 4, consider presenting the effect size of intervention of HAZ as a 95% confidence interval. Also, please consider revising how you have presented the information on this table as it is not very clear. Columns 4-6 appear to be missing.
We have now included 95% confidence intervals to Table 4 (see also the response to point 2 above). We have revised Table 4 in line with the suggestions of the statisticians who reviewed the paper. We hope that these changes have made it easier to read. We apologise for the typo in the notes which seemed to indicate that there should be 3 additional columns in the table. The Table should only have 3 columns as presented.

Page 16, line 10: Fitzsimons et al (2016) Please provide reference this in line with agreed format.
We have corrected this to ensure that the reference is in the agreed format.

Reviewer 2: Alison Talbert
The paper looks at the effect of a health promotion intervention delivered to mothers on child growth and how its impact is modified by the presence of living relatives such as grandmothers and parents' siblings. The subject is of interest to those working in public health in Africa and other LMICs where extended families are prevalent. The author presents a secondary analysis of data collected from surveys of families in 2008 -2010 to look at the effects of a peer counselling intervention running from 2004 (to present day?) to mothers of infants under 6 months on child growth, and a new qualitative study on the impact of extended family members that was done in 2015.
We thank the reviewer for acknowledging this is a subject of interest. The peer counselling intervention trial closed in 2010. However as the peer counsellors are volunteers many continued to work after this time, with support from their communities, and many were still active at the time of the qualitative data collection in 2015, as mentioned in the paper.

Unfortunately the results are presented in such a way that it is confusing for the reader and I feel that a major revision of the tables is needed, preferably with a statistician's input.
We have amended the tables following the suggestions from the statisticians who reviewed the paper. We hope that they are now much clearer and easier to follow.

It should be clearly stated here that this is a secondary data analysis of a previous RCT with an additional qualitative study.
We have included a sentence in the abstract that makes clear that this is a secondary, sequential mixed-methods study based on a cluster RCT. The new text (p.2) is:
This is a secondary, sequential mixed-methods study based on a cluster randomised controlled trial of a peer home-education intervention conducted in Mchinji District, Malawi.

Line 42 - change "reduction in height" to "reduction in height-for age z score"
We have incorporated this change in the text.

Introduction
There is no mention of the normal extended family living arrangements for this population. It is important to describe if they are patrilineal and/or patrilocal because this might explain the greater impact of the paternal grandmother if she has a high status and is a resident member of the household rather than the maternal grandmother who would be living elsewhere.
Following the referee's suggestion, we have included more detail about the extended family living arrangements in Mchinji when describing the study setting. Though the referee suggested including this in the Introduction, we felt that it was better placed when describing the setting. The new text on p.7 is:
"Traditionally, the main ethnic group in the study area, the Chewa, are a matrilineal and matrilocal group. Matriliny is a system in which land is passed through the female line. Under traditional matrilocal norms, husbands move to their wives’ homes after marriage unless they make a special

payment. However, following the influence of patrilineal and patrilocal ethnic groups and British colonialists, there is evidence that matrilocality has waned over time, but not completely [26] [27]. As a result, women often remain in close proximity to their own relatives after marriage.

Strengths and limitations
Line 16 needs rephrasing, I suggest: "quantitative analysis allows estimation of the size of effect different extended family members have on the impact..."
We have amended the text to incorporate this suggestion.

Page 7 line 6 At what ages was height of the children measured and by whom? How were Z scores calculated (WHO MGRS?)
Children were aged between 0 and 53 months when they were measured. Our sample was designed so as to measure all children aged under 6 years at the time of the follow-up surveys in 2008-09 and 2009-10, rather than to measure all children at specific ages. Children were measured by trained enumerators. The z-scores were calculated using the WHO MGRS. This is now detailed in the "Data and Sample Selection" section (p.8):
"The height of the main respondent and the height and weight of children under 6 years were also collected by trained enumerators.
The main outcome for our analysis is the child height-for-age z-score (HAZ score), which is a long term indicator of health that reflects nutrition and morbidity since birth, and should be sensitive to any effects of intervention exposure in early life. It is calculated by comparing the height of the child with the median height in the World Health Organization reference population of children of the same gender and age in months [26]."

Page 7 line 41 "Balance was preserved" - wording needs changing as it suggests an active process of balancing. Need to describe the baseline characteristics of the 2 groups as being similar
We have changed the phrasing as suggested to "The baseline characteristics of the two groups remained similar even after accounting for attrition between the baseline and first endline survey, indicating that randomisation was not jeopardised." (p. 8)

Page 9 line 24. I think that the analysis would have been improved if the model tested the impact of a grandmother or other family member who lived in the same household or nearby. In this case the grandmother would be more likely to give advice than one who was alive but not likely to be in face-to-face contact. If you have data on this you should test the model including this variable.
We thank the referee for this interesting suggestion. While we acknowledge that using variables associated with living extended family members lacks nuance, it is conceptually very challenging to empirically differentiate between grandmothers/extended family members living close by and those living further away, since grandmothers/extended family members could choose to locate close to the child's household because of a child or household characteristic that also relates to child height. For example, grandmothers may choose to move closer to grandchildren who are weaker and require more care. Thus, differentiating effects by geographical distance in this way could lead to misleading conclusions. For this reason, and given that the manuscript is already quite lengthy, it was decided not to include further such analyses in the paper.

Page 10 line The FGDs with peer counsellors and others were conducted a long time after the intervention started so there might be recall bias. Also the peer counsellors had a high attrition rate which might affect the findings.
We agree that there was the potential for recall bias within the qualitative data collection, and have included additional text to the limitations section of the discussion to acknowledge this. In terms of the peer counsellor attrition, we invited counsellors from areas with high and poor engagement with the intervention (as mentioned in the methods section), in order to try and elicit a range of views,

including currently active and inactive counsellors. We agree, targeting currently active counsellors only would have introduced a respondent bias.
"Finally, the qualitative and quantitative mixed data were collected sequentially rather than concurrently, making interpretation more challenging with the qualitative data collection conducted five years after the quantitative survey. This may have resulted in recall bias in the qualitative data, and the culture and behaviours around infant feeding may have shifted between the two study phases. This is somewhat supported by the FGDs and interviews from control areas being exposed to the peer counsellors and their messages, posing a challenge to integrating the results. However, as the qualitative data was planned to provide a more in-depth understanding and triangulation of the quantitative findings, rather than drawing conclusions comment on causality, therefore we do not feel this detracts from the our interpretation. (p.19)"

Page 10 line 18 purposively is the more common term
We thank the referee for this clarification. We have amended the text to use this term.

Page 13 line 48 Language is confusing - it reads as if breastfeeding, weaning and complementary feeding messages were not the focus (when they were) when you mean the extended members were not the target of the messages

-Thank you for picking this up - we have amended the text to make it clear. The new text is:
"Despite extended family members not being the target group focus of the intervention, breastfeeding, weaning and complementary feeding messages appear to have disseminated (p.16)"

References Page 19 line 6 The first reference needs information on name of publishers
We have included this.

Tables need to be presented differently - I suggest an expert statistical review. Layout is confusing for the reader and numbers are not explained clearly - some are proportions, others averages and others regression coefficients

-Thank you for pointing this out. We have amended the tables in line with suggestions from two statisticians who reviewed them.

Table 1 Change the title to something like Household and mother's characteristics at baseline survey
Table 1 Only the control group's characteristics are shown but both should be shown. No need for p values
Table 1 it is not clear what the numbers in the 1st column represent - some are averages others are proportions. For the characteristics better to put e.g. how many households in each group have electricity (n) then percentages Table 1 - maybe put in a row for the composite wealth index which is mentioned in table 3.

-The reviewers offered mixed views on how we could improve the Tables. We chose to follow the suggestions of the two statisticians who reviewed the paper. Following their suggestions, we have amended the title to the table to better reflect the information it is presenting.
This Table serves two key purposes: First, it allows us to provide an overview of the sample, by analysing characteristics of the control group sample at baseline; and second, it allows us to assess whether the analysis sample in the treatment and control groups had similar baseline characteristics, given the encountered attrition and modified sample selection criteria.
We have also made clear in Table 1 (using a footnote and the % symbol) which of the reported numbers are means and which are proportions. Finally, we have included the composite wealth index in the Table.

Table 2 - as in table 1 show the results for each group (control and intervention) in separate columns rather than only for control and second column showing difference from control. No need for p values for baseline characteristics. Table 2 - better to label numbers as n and percentages for grandmothers, and others as average numbers.
-As above, we followed the suggestions from the statisticians to improve the table. In addition, we have made it easier to see which variables are binary and which are continuous.

Table 3 - needs laying out differently. Give 95% confidence intervals for the regression coefficients rather than standard errors and p values.

-As above, we followed the suggestions from the statisticians to improve the clarity of the Table. Since the study has a small number of clusters (24), we use a wild cluster bootstrap-t procedure recommended by Cameron, Gelbach and Miller (2008) for inference in this paper. This method provides us with a p-value, which we report in the draft. Calculating the associated confidence intervals is not straightforward but can be done using a method suggested by Cameron and Miller (2015). This method relies on simulations, and is thus very computationally intensive. It is also not available in standard statistical packages, and so we have had to write an ad-hoc program to compute the confidence intervals. Given that this process is very computationally intensive (it has taken us around 2 days to obtain the confidence intervals for Table 4, once the program was written), we have only calculated these for Table 4.

Table 4 needs major revision. What do the column headings mean? 1 seems to be crude estimate and 2 and 3 are adjusted estimates according to different models but the rationale for using the different models is not explained in the methods section of the paper. Table 4 notes: it mentions columns 2-6 but there are only 3 columns in the table. This looks like copy and pasted notes from elsewhere.

-We apologise that the Table and the associated methods and results sections were not very clear or easy to follow. We have revised the methods and results sections to better explain the methodology, and how it links with the results reported in Table 4. In particular, we have added more detail about the models presented in Table 4. In Table 4, we have renamed the columns in the table to link them to the models presented in the methodology section. Finally, there was a typo in the Table notes which suggested that there ought to be 6 columns rather than the 3 presented. We have corrected this. We hope these changes have made the table clearer, and easier to follow.

Appendix 1 Number of households interviewed in the peer counselling clusters follow up survey of Oct 2009 -Jan 2010 differs from that in Fitzsimon's paper (753 vs 752).
-This was a typo. The number of households interviewed is as in Fitzsimons' paper. This has been amended in Appendix 1.

Reviewer: Peter Rohloff
I really enjoyed reading this important study an a topic that often goes neglected - the impact of extended family members on behavior change interventions for improving child growth.

-Thank you for the kind comment.

Abstract
-I would recommend reporting confidence intervals rather than p values in the abstract.
-We have now reported the 95% confidence intervals. Since the study has a small number of clusters (24), we use a wild cluster bootstrap-t procedure recommended by Cameron, Gelbach and Miller (2008) for inference in this paper. Calculating the associated confidence intervals is not

straightforward but can be done using a computationally intensive method suggested by Cameron and Miller (2015). We use this method to calculate the confidence intervals.

Strengths/Limitations boxes are bit generic, and generally just repeat text already evident from the abstract. Would be explicit about limitations here, and also use these bullets as an opportunity to make policy or programming points and really emphasize the importance of the findings

-Thank you for this suggestion. We have amended this section by merging similar points into one bullet point, adding a limitation related to the difference in timing between the quantitative and qualitative data collection, and adding a more policy/programming specific point.

Theoretical framework in the introduction could use a bit of refining. This is especially evident in the paragraph starting on page 4, line 47 - this brief literature review brings together citations from what really comprise a quite wide range of different kinds of programs and strategies (e.g, home based one on one caregiver counseling vs primary school programs). I'd suggest focusing the introduction more narrowly on just on the type of behavior change/education strategies that are relevant to the described intervention (e.g., interventions for caregivers of young children), and then describe in a bit more detail how evidence for efficacy within that narrower group is "mixed"

-We thank the referee for this comment. We have now made the introduction and literature review more focused by including only literature on education strategies targeting infant feeding and care in developing country settings.
The new text is (p.5):
"Education campaigns promoting better infant feeding and care to care-givers of infants have shown mixed success in developing country settings. In some cases, they have led to sustained improvements in feeding practices (Morrow, et al. 1999), (Olney, et al. 2015) and child physical growth (Guldan, et al. 2000), (Bhandari, Mazumder, et al. 2004), (Penny, et al. 2005). In others, they have had a negligible effect on child physical growth (Engebretsen, et al. 2014), (Bhandari, Bahl, et al. 2001), (Guyon, et al. 2006). Given these mixed findings, there is a need for greater understanding of the factors shaping responses to such interventions across contexts."

Page 5, Line 8 - this is true, there isn't a lot of literature on this topic, but there is some - including several studies that the authors cite a few paragraphs earlier. I'd expand this section a bit and engage what literature is available, so that readers have a better sense of the state of the field. This is especially important, because the author's work here involves a really large data set and therefore has something very important to add to what amount to group of smaller, mostly qualitative studies.

-We thank the referee for this comment. We have expanded the discussion of the literature on the role of extended family members in influencing the success of care-giver focused education interventions in low income settings.
The new text (p.5) is:
"Little attention has been paid to the role of extended family members in influencing the success of care-giver focused education interventions in the existing literature, despite their important role in shaping child health. Much of this existing literature is qualitative, with small samples (Mukuria, et al. 2016), (DeLorme, et al. 2018). The small number of quantitative evaluations of education interventions to improve infant feeding that seek to involve extended family members find mixed evidence of effectiveness. Counselling sessions for new adolescent mothers and co-resident grandmothers reduced the un-necessary intake of water and herbal teas within the first 6 months of the child's life in Brazil (Nunes, et al. 2011) but failed to maintain breastfeeding of infants at age 2 years (da Silva, et al. 2016); while a behavioural change communication program delivered through

older female leaders in Burkina Faso improved infant feeding knowledge but failed to improve child health outcomes (Olney, et al. 2015)."

A few details of the qualitative approaches could be clarified. "All translations agreed between the researchers" - does this mean that transcripts were initially into Chichewa and then translated into English? How was agreement determined? Were the FDG facilitators different individuals than the authors here who conducted the transcribing and coding? If coding was conducted independently by at least two authors, does this mean that all transcripts were double-coded and are values for inter-coder reliability available? If not, and instead authors worked together to achieve complete coding consensus, then what procedures were used to identify coding discrepancies and resolve differences?

-We have now included more detail to the qualitative data collection and analysis sections - which we agree were not as detailed as they could have been. The transcripts were all double coded, and then the overall framework and themes were agreed through discussion over a two day period in Malawi.

In the methods (and also results) the pooling procedure used for data raises a few points for me that could be clarified. Most generally, what was the rational for two followup surveys? Was the plan to analyze certain variables longitudinally, or was this just an effort to capture data missed on the first round? I ask in part because in Tables 2-3 it says only the second survey was used. Does this mean that no data was missing or that data from the first survey wasn't used to complement the second? If data was pooled in some way then obvious that raises other questions, such as two conflicting answers to the same question by one subject between the two surveys, or family members dying between survey points.

-We fielded two follow-ups in order to analyse some other outcomes (published elsewhere) longitudinally. The outcomes studied in Table 3 are fixed over time, so we chose to focus on one round of data only. Including both rounds together wouldn't change any of the statistics.
As mentioned by the referee, respondents could have provided different responses to the number of extended family members in the two follow-ups, due to either family members dying, or a change in marital status (e.g. divorce or marriage/re-marriage) or because of misreporting. For respondents for whom there is no change in marital status, or location between the two follow-ups, we attribute any change in the reported number of siblings of the main respondent and her spouse to misreporting, and take the average value of the two reported values as the measure of the extended family network. Using the average value of the two potentially misreported values rather than the potentially misreported value itself will provide coefficient estimates that are subject to a lower bias when the misreporting is random (which we believe to be the case here). For mothers, and mothers-in-law of the main respondent, which are the key indicators of the extended family network, we encountered fewer misreporting issues (which were corrected where possible). We thus use the response from each round for the associated HAZ score for that round.

Throughout the results section and Tables I would find reporting of confidence intervals more informative than P values, and this would also be in line with more typically writing practices for randomized trials. Adding standard deviations to continuous variables (or IQR and media of not parametric) would also aid the reader. This is not a major point however and I would defer to the editors.

-Since the study has a small number of clusters (24), we use a wild cluster bootstrap-t procedure recommended by Cameron, Gelbach and Miller (2008) for inference in this paper. This method provides us with a p-value, which we report in the draft. To calculate the associated confidence intervals, we apply a method suggested by Cameron and Miller (2015). This method relies on

simulations, and is thus very computationally intensive, and also requires a number of adjustments to the simulation parameters. Moreover, this method is not available in standard statistical packages, and so we have had to write an ad-hoc program to compute the confidence intervals. Given that this is very computationally intensive (it has taken us around 2 days to obtain the confidence intervals for Table 4, once the program was written), we hope that it's fine that we have only calculated these for Table 4 only.
We have amended the Tables in line with suggestions from the statisticians who reviewed the paper. We note also that amending the layout as you suggest would increase substantially the number of columns in the Tables, making them more complex and difficult to follow.

Table 1- the description about the sample containing children born after 2005, second survey, etc i s not pertinent to this table which contains only the caregiver and household level data from the 2004 baseline, correct?

-We thank the referee for pointing out the confusing nature of the table note. We have amended it to make it easier to identify the sample used to construct the table.

Table 2: The 0/1 indicates that for grandparents, this is the proportion of families with a living grandparent? This is proportion vs means for the other variables in the tables?

-We have amended Table 2 to make it easier to follow. In particular, we have reported the 0/1 variables as percentages, and made clear which variables are binary and which are discrete.

Appendix 1 - drop off at followup survey. I understand that randomization was preserved (Table 1) despite drop off, but it would also be very interesting to see characteristics of subjects/families who dropped off vs those who were successfully interviewed (perhaps in the Appendix)

-An analysis of the characteristics of those who attrited and those who didn't has been conducted and reported in Fitzsimons et al (2016) (see Table A1, Supplementary Appendix A). They find that respondents who were married, older, less educated, and working in agriculture at baseline were less likely to attrit. Similarly, respondents who were from households constructed with poor materials, and agricultural households were less likely to attrit. We have added some text on this to Figure 1 in Appendix 1.

For Table 3, the line about restricted data to one eligible child is not pertinent, correct, because this is again all caregiver/household level data? Can the wealth index reference be added to the manuscript?

-The Table note was confusing and didn't explain clearly the sample used in constructing the Table. We have amended the note to make it less confusing. We have also added a reference to the manuscript of the wealth index.

For Table 4 (and corresponding methods) I have the same question about how the data from the surveys was pooled. HAZ tends to decline most around 2-3 yrs and then recover some. So, were children potentially measured twice and, if so, which HAZ was used. Also for this Table and corresponding text, it would be nice to see estimates of each model's performance (e.g R2)

-In pooling the data from both surveys, we include (where available) both observations of a child's HAZ, each of them as a separate observation. The inference method we use (wild cluster bootstrap-t) allows for arbitrary dependence in observations of the same child as well as amongst observations from different children in the same cluster.

Qualitative results: I kept hoping for some data that would help differentiate between maternal and paternal grandmothers, and I assume that such data was not available to the team?

-We too were hoping for more distinction between maternal and paternal grandparents to be elicited from the qualitative data. Unfortunately, despite probing questions on any potential differences, participants did not discuss or acknowledge any differences in the roles or influence between parental and maternal grandmothers. We highlight this disparity between the quantitative and qualitative results in the discussion.

Some additional context on the study sites, and local cultural practices might also help to explain or better interpret the results. I find myself wanting much more explanation (potentially even speculation) from the authors about this differential impact. Why do paternal (and not maternal grandmother's command so much more influence? In cultures I'm more familiar with (in Mexico and Central America) we see exactly the same thing, and this is because new families usually take up patrilocal residence with the father's parents - hence the grandmother who is available and constantly present to exert influence is the paternal grandmother. Some details of this nature would be really helpful

-We have now included additional background information on the matrilineal nature of the Chewa tribe, to provide this context. We agree that this was lacking to help the reader interpret the results. We were hesitant however to speculate on the differences seen in the quantitative results between paternal and maternal grandparents, in part (related to your comment above) as we expected to elicit this from the qualitative data. As the manuscript is already lengthy, hypothesizing about the maternal/paternal nuance was deemed to require too much additional explanation.

Also it would be nice to have some interpretation of the findings from the regression analysis of the negative impact of maternal grandmother presence (independent of the intervention) on growth, since this is the other major element of the regression analysis.

-We need to be cautious when interpreting the negative coefficient associated with maternal grandmothers since it is not a causal estimate, and could reflect other confounders (e.g. increased competition for resources in matrilineal societies). Any interpretation would thus be purely speculative, which we wanted to refrain from doing. We have included some text in the Discussion section outlining this.

Reviewer: Janet Smylie
lack of clarity with respect to what is meant by "traditional" practices and assumption that these practices undermine interventions. If the authors are referring to pre-colonial health practices, these are extremely complex and interwoven with colonial influences

-This is a good point - by traditional we mean the non-Western health beliefs, practices and behaviours which are common across much of sub-Saharan Africa. In this setting specifically, they include rituals to fend off witchcraft and the use of herbal medicines. We agree that this is a complex belief system, and not necessarily always at odds with Western medicine, however it is outside of the scope of this paper to explore this in more detail. We included a statement in the 'Settings' section of the methods to describe this.
The new text is as follows (p.6):
"However, Malawi has medical pluralism, with traditional practices, beliefs and behaviours such as witchcraft and herbal medicine use being commonly used alongside Western medicine."

Qualitative methods lack of specification of theoretical frame, analytic lens, and social location of research team who did the analysis - given the cross-cultural context this is essential

-We have rewritten much of the qualitative methods section to include more detail about the analysis approach and the research team who collected the data and analysed it. We took a pragmatic approach to the qualitative analysis and presentation, suitable for a multidisciplinary research team and more generalist readership.

brevity of presentation of qualitative findings - does not allow for an assessment of whether or not saturation appears to have been reached, lacks contrasting findings, makes it hard to understand context of presented findings and assess truthworthiness of findings

-Presenting the manuscript as a single mixed-methods study was important to us, in order to strengthen the interpretation of both sets of data. However, this has limited the space available to present the qualitative findings in a more in-depth manner. We therefore presented a narrower descriptive set of results which we deemed relevant for the triangulation of the quantitative findings. We have added more detail to the methods to improve intrepretation of both sets of data.

the interpretation of the qualitative findings seem to be hinged on the use of "traditional" views on childrearing by grandparents interfering with "standard practice" however the results on "traditional practices" only cite on example and state that these practices are uncommon or hidden
-Based on the integrated interpretation of the quantitative and qualitative data, we proposed that tension between traditional and Western medicine practices in infant feeding may have been important. We have tried to keep the presentation of the qualitative findings succinct, however agree that this section, given its importance in our interpretation, could be expanded.

Quantitative methods appear sound but should be reviewed by a statistician - seems like underpowering may have been an issue
-The paper was reviewed by two statisticians and their comments have been addressed.

Reviewer: Francesco Sera
I have only some doubt on the models that evaluate the relationship between some covariates (household and mother characteristics) and the possible modifiers (family network indicators). Some covariates are categorical or even dichotomous and these variables have been considered as outcome in OLS models with family network indicators as possibly explanatory variables. It would have been more appropriate using logistic or polytomous logistic regression models for this analysis.

-We thank the referee for this suggestion. We estimated the regressions reported in Table 3 using a logistic model for the binary variables, and using a block bootstrap for inference. We obtain qualitatively similar results, with similar coefficient values and p-values in most cases. However, it is not clear whether the wild cluster bootstrap-t method is suitable for inference in non-linear models. Given that we have both continuous and binary dependent variables in Table 3, we prefer to use a linear model so that we can use the wild cluster bootstrap-t throughout for inference.

Tables can be improved.
Table 1. The title should be changed in something like “distribution of household and women characteristics in controls and differences with treatment group”. For categorical variables the percentage should be reported for absolute values and differences.
-We thank the referee for this suggestion. We have amended the table accordingly.

Table 2. Also here the title should be changed in “distribution of family networks indicators in controls and differences with treatment group”. For dummy variables the percentage should be reported for absolute values and differences. There is no need to report the coding (0/1).
-We have amended the table according to the referee's suggestion.

Table 3. The numbers in brackets should be indexed as SE and p values in the rows. In panel B the symbol “#” need to be replaced by “Numbers of”.
-We have amended the table according to the referee's suggestion.

Table 4. The title need to be changed. Something like “Coefficients and (SE) of the multivariate linear models evaluating the interaction between treatment and family network indicators”. The number in brackets should be indexed as (SE), perhaps building two columns one for the coefficient and one for the SE. (1) (2) and (3) should be replaced by Model 1, Model 2 and Model 3.

-Following the suggestions from this and other reviewers, we have revised the title to the Table and the Table to make it easier to follow. We have also now included 95% confidence intervals to this table, and have dropped the p-values.

Reviewer: Scott R Walker
The following comments relate only to the statistical methods used in this paper. In general, the proposed model seems appropriate to answer the main research question and the sampling strategy seems sound. However, there are some areas where clarity could be improved. There are also issues with model interpretation that have implications for the study’s conclusions.
1. In the results section of the abstract the authors make the claim that there was “no effect of parents’ siblings.” A non-significant result does not indicate that there is ‘no effect’, but rather that there is ‘no evidence of an effect’. This may seem like a small distinction, but the former claims something concrete about an effect (that there is none), whereas the latter indicates a lack of evidence on which to make a claim.

-Thank you for pointing this out. We have changed the wording to reflect this nuance.

2. Methods: Model Specification and Estimation The description of the model would be enhanced by more explicit description of how the model relates to the research question. Specifically, that the interaction terms assess whether certain family members modify the intervention effect. A positive (or negative) negative significant interaction indicates a greater change in HAZ score than would be expected due to the intervention alone and provides evidence that the intervention effect is enhanced (or diminished) in the presence of that particular family member.

-This is a very useful suggestion. We have revised and expanded the methodology section to make the connection between the quantitative estimation and research question more explicit. We found your explanation extremely clear, and borrowed some of your wording for the draft. We hope you don't mind!

New text (p.11):
“We fitted three models, one crude model with the intervention term only, and two full models as specified in the equation each treating parent siblings differently.

The coefficient β captures the effect of the program for children whose maternal and paternal grandmothers are dead, and whose parents are only children. The coefficients β_3, β_5, β_7 and β_9, associated with interaction terms between variables capturing extended family relations and the indicator for program allocation, estimate the additional effect of the program for children with different types and numbers of extended family members. A positive (or negative) significant interaction provides evidence that the intervention effect is enhanced (or diminished) in the presence of that particular family member. Through the coefficients β_2, β_4, β_6 and β_8, the specification also accounts for the possibility that, independently of the intervention, the HAZ score might be different depending on what family members are alive. “

3. Results: Table 3 displays an extensive set of results derived from analyses that appear non-trivial. Where the methods are sketched out in a footnote of the table, perhaps it makes more sense to describe the methods in the methods section itself and provide a little more detail. This would also allow space to give some justification for this part of the analysis and how it supports the main research question. As it is now, the results Table 3 pop up a little unexpectedly and its relevance only becomes clear after some scrutiny by the reader.

-We agree that Table 3 seemed to pop-up unexpectedly without warning, and that its purpose was not very clear. We have restructured the data, methodology and results sections to improve the flow of the paper. In particular, we moved the introduction and explanation of the analyses presented in Table 3 to the Methodology section as suggested. Moreover, we have included some more detail on the methodology underlying Table 3 in the main text itself. We hope these changes have made this analysis easier to follow.

Results: Table 4 represents the main findings relative to the study aim, but would benefit from some additional labelling to make it easier to interpret.
a. At first it wasn't obvious what the three columns of results represented. I could work out that 1 was a crude model, and 2 and 3 were adjusted models with parent siblings in different formats but some kind of column headings would help to make this more immediately apparent. In retrospect I saw that this had been described in the methods but the description hadn't made sense until I got to Table 4 and went back to the methods specifically looking for it. So clarifying the three models in the methods would be a big help too. The fact that the equation comes first in the methods gives the impression that there is one model, then the language used to describe the three models doesn't do much to change that first impression. Perhaps something like this would be more explicit: "We fitted three models, one crude model with the intervention term only, and two full models as specified in the equation each treating parent siblings differently…"
b. It was not immediately apparent that the numbers in brackets were p-values. It is mentioned in the footnote, but it might be clearer to put it in the title, e.g. "Table 4. Estimated height-for-age scores {with p-values} from three linear regression models".

-We agree that Table 4 was not very easy to follow. Following your and the other reviewers' suggestions, we have amended the Table to make it easier to follow and link with the methodology section, which has also be expanded and restructured following the comments above. As suggested, we include a sentence in the methodology section to sign-post to readers that we estimate 3 models. We have amended the Table to include 95% confidence intervals instead of p-values. We have updated the Table title to make this apparent.

The interpretation of the model estimates seems to be the key to addressing the question of whether family members influence (i.e. are associated with) the intervention efficacy, so it is important to get this right.
-We absolutely agree, and thank you for your suggestions to help improve this.

Results: In paragraph 4 of the Results, there is an initial reporting of the overall intervention effect (from the crude model) of 0.296, which all seems fine. Then later in the paragraph there is a claim that the intervention effect is 0.235-0.253 which doesn't make sense as these are the estimates for the interaction term 'paternal grandmother*T'. First order terms, e.g. 'maternal grandmother', indicate the pre-intervention difference in HAZ score between settings where the is the MGm versus settings where there is not. So in Table 4, having the MGm present pre-intervention is associated with reduced child height compared to families with no MGm present (using model 2: difference in HAZ = -0.265, p=0.05) . For the paternal grandmother, however, there is no evidence of a difference if the PGm is present or not pre-intervention (difference in HAZ = 0.008, p=0.99).

For the interaction terms, e.g. 'MGm*T', this indicates changes over time where a family member is present in addition to the intervention effect. So where the MGm was not present, HAZ was associated with an increase of 0.441 with the intervention. When the MGm was present this increase in HAZ was augmented by 0.168, resulting in an increase of 0.441+0.168=0.609. In contrast, when the PGm was present the intervention effect was reduced by -0.253 resulting in a smaller increase of 0.441-0.253=0.188 with the intervention.
Having spelled out how the interaction terms are interpreted in the methods (comment 2), this kind of reporting of results will hopefully be easier for readers to follow.

-Many thanks for raising this point. We have revised the methodology section and added text to explain how the different coefficients ought to be interpreted. We have also amended the Results section to make this clearer.

Some of the issues in the comment above carry over into the discussion where the model results are interpreted and discussed. Currently the text claims that grandmothers can be a barrier to the intervention and behaviour change. This is not quite what the results show. Rather the main findings seem to be:
i. Prior to the intervention, maternal grandmothers were associated with lower HAZ scores. It is important not to overstate the direction of causality here. It may not mean that the maternal grandmothers were causing the children to be undersized. Could it be that grandmothers are more likely to be present when children are undersized in the sense of offering support? Or are there other possible explanations? In contrast paternal grandmothers did not seem to be associated with child size pre-intervention, and the authors may have some plausible explanation for this.
ii. The intervention-related increase in HAZ score was amplified in the presence of maternal grandmothers but reduced in the presence of paternal grandmothers. This suggests only paternal grandmothers may be a barrier to the intervention, while maternal grandmothers increased its success. In revising the discussion, it will be important to address potential reasons for the differential effects on the intervention between maternal and paternal grandmothers (the 'in-law' effect?!) in a way that is consistent with interpretation of the differential pre-intervention results mentioned in 5.b.i.
iii. There was no evidence of an association with HAZ score for other family members, either pre or post intervention. This lack of evidence bolsters the case for focusing on inclusion of grandmothers in the design of interventions as discussed in paragraphs 4 and 5 of the discussion.
In light of this, particularly the differential pre and post effects of grandmothers, much of the discussion needs reworking to more closely align with the results of the model.

-Response to (i): Given the limited space in the manuscript, and also that the coefficient on MGm cannot be interpreted in a causal manner, we refrained from speculating on why it was negative. We are still cautious about doing this, but have now added some text in the Discussion section expressing this concern and our reluctance.
Response to (ii): We had hoped to shed light on mechanisms for the different effects of paternal and maternal grandmothers through the qualitative study. Unfortunately, despite probing questions on any potential differences, participants did not discuss or acknowledge any differences in the roles or influence between parental and maternal grandmothers. We note this disparity in the Discussion section. We also note that the interaction term between the intervention and the indicator for Maternal Grandmother is not statistically significant from 0, which makes us very cautious in contrasting the differential interaction effects of paternal and maternal grandmothers.
Response to (iii): We agree with this.

VERSION 2 – REVIEW

**REVIEWER**	Peter Rohloff

	Maya Health Alliance - Guatemala<br>Brigham and Women's Hospital - USA
**REVIEW RETURNED**	06-Feb-2018

**GENERAL COMMENTS**	I think the authors have done a nice job responding to the extensive and detailed commentary for the multiple reviewers and the manuscript is much improved at this point, I think it is acceptable for publication.

**REVIEWER**	Alison Talbert<br>Centre for Geographic Medicine Research - Coast<br>Kenya
**REVIEW RETURNED**	10-Feb-2018

**GENERAL COMMENTS**	The authors have responded to the reviewer's comments fully changing the manuscript to give more information on the family context and clarifying methods of analysis. The tables are presented in a easily understandable format.<br>I am satisfied with their revisions, and their explanations when they have chosen not to revise certain sections.

**REVIEWER**	Francesco Sera<br>London School of Hygiene and Tropical Medicine,<br>London<br>UK
**REVIEW RETURNED**	10-Feb-2018

**GENERAL COMMENTS**	The authors have answered exhaustively to all my comments.<br>Overall, also taking into account the comments from other reviewers, the manuscript has considerably improved from the original submitted version.

**REVIEWER**	Scott R Walter<br>Macquarie University, Australia
**REVIEW RETURNED**	10-Feb-2018

**GENERAL COMMENTS**	The authors have made a commendable effort in revising the paper and in many respects it is considerably improved. There are still some issues with the interpretation of the model coefficients that need to be addressed, but the in terms of changes to the manuscript this should be relatively minor.<br><br>1. In the first line of the Abstract results and also in the start of the Results in the main text, there is an implied causality that may be overstating things. It is not known that for sure that the program raised the HAZ score, but only that HAZ scores were 0.296 SDs higher in the intervention group compared to the control group: a version of the old ‘correlation is not causation’ adage. I would suggest rewording with something as simple as “overall exposure to the program *was associated with* raised HAZ scores …”<br><br>2. The results in the Abstract and main text both claim that having a maternal grandmother alive is associated with lower HAZ scores *independent of* the intervention. The estimates for maternal grandmother (beta 2) do not represent independent effects, as would be the case for a first order term in a model without interaction terms. When the model includes interactions between binary variables beta 2 actually represents the effect of maternal

	grandmothers in the *control group only*. To get a general estimate of the maternal grandmother effect independent of the intervention would require fitting a different model.<br>This point also applies to the added description of model terms in the methods. Betas 2, 4, 6 and 8 are effects for the control group only, i.e. when T=0.<br><br>3. Also in the description of model coefficients, the explanation of betas 3, 5, 7 and 9 would be more complete if it read "the intervention effect is enhanced (or diminished) in the presence of that particular family member, *relative to the family member effect in the control group*".<br><br>4. When reporting results of the models in the main text, it seems a little selective. Why not report all results related to grandmothers (i.e. betas 2, 3, 4 and 5) for completeness as this one of the main aims of the paper? The first order and interaction effects were quite different for maternal vs. paternal grandmothers, and reporting all four estimates gives a clear picture of this.<br><br>5. At the end of the Abstract results and the beginning of the Discussion, claims are made about grandmothers in general. However, as the comment above notes, the results for paternal vs. maternal grandmothers are very different and it doesn't make sense to conflated them. In particular, the strong claims about grandmothers being a barrier to the intervention is not true of maternal grandmothers where the interaction effect indicated an enhancing effect, albeit non-significant. And, related to point 1, these statements imply some causality that can't be known for sure.<br><br>6. As a general comment following on from many of these points, the authors should consider using more circumspect language when interpreting results. The models only indicate associations and it is important not to assume too much about causal pathways. Relatedly, it is also important not to treat non-significant results as evidence, since they are rather a lack of evidence, i.e. we can never accept the null hypothesis.

VERSION 2 – AUTHOR RESPONSE

Many thanks for your encouraging comments, and for the time you have taken to read the paper. Your comments have been extremely useful in improving the paper.

In what follows, we provide a detailed reply to each comment, one by one. For ease of reading, we first reproduce the original comments, before providing our response and outlining any revised text.

Reviewer: Scott R Walker
The authors have made a commendable effort in revising the paper and in many respects it is considerably improved.

Thank you for the kind comment.

There are still some issues with the interpretation of the model coefficients that need to be addressed, but the in terms of changes to the manuscript this should be relatively minor.

1. In the first line of the Abstract results and also in the start of the Results in the main text, there is an implied causality that may be overstating things. It is not known that for sure that the program raised the HAZ score, but only that HAZ scores were 0.296 SDs higher in the intervention group compared to the control group: a version of the old “correlation is not causation” adage. I would suggest rewording with something as simple as “overall exposure to the program was associated with raised HAZ scores”

It is true that we need to be careful about the distinction between correlations and causation when interpreting the findings, particularly the coefficients on the interaction terms as we discuss below. However, since we have a randomised control trial, we can interpret the effect of the overall exposure to the intervention on HAZ scores, estimated using an intention-to-treat estimator, as a causal effect.

2. The results in the Abstract and main text both claim that having a maternal grandmother alive is associated with lower HAZ scores *independent of* the intervention. The estimates for maternal grandmother (beta 2) do not represent independent effects, as would be the case for a first order term in a model without interaction terms. When the model includes interactions between binary variables beta 2 actually represents the effect of maternal grandmothers in the *control group only*. To get a general estimate of the maternal grandmother effect independent of the intervention would require fitting a different model.
This point also applies to the added description of model terms in the methods. Betas 2, 4, 6 and 8 are effects for the control group only, i.e. when T=0.

We apologise for the confusion caused by our use of the phrasing “independent of the intervention”. We have followed your suggestion and changed this to refer to the control group only.

3. Also in the description of model coefficients, the explanation of betas 3, 5, 7 and 9 would be more complete if it read [3]the intervention effect is enhanced (or diminished) in the presence of that particular family member, *relative to the family member effect in the control group*[2].

We have restructured this paragraph, which will make it clearer that these coefficients are also capturing effects that are relative to the family member effects in the control group. The revised paragraph (p. 11) is as follows:

“The coefficient β captures the effect of the program for children whose maternal and paternal grandmothers are dead, and whose parents are only children, while the coefficients β_2, β_4, β_6 and β_8, represent the effects of the extended family members on HAZ scores in the control group. The coefficients β_3, β_5, β_7 and β_9, associated with interaction terms between variables capturing extended family relations and the indicator for program allocation, estimate the additional effect of the program for children with different types and numbers of extended family members. A positive (or negative) significant interaction provides evidence that the program effect is enhanced (or diminished) in the presence of that particular family member.”

4. When reporting results of the models in the main text, it seems a little selective. Why not report all results related to grandmothers (i.e. betas 2, 3, 4 and 5) for completeness as this one of the main aims of the paper? The first order and interaction effects were quite different for maternal vs. paternal grandmothers, and reporting all four estimates gives a clear picture of this.

We thank the referee for this suggestion. We have amended the text to incorporate this.

5. At the end of the Abstract results and the beginning of the Discussion, claims are made about grandmothers in general. However, as the comment above notes, the results for paternal vs. maternal

grandmothers are very different and it doesn¹t make sense to conflated them. In particular, the strong claims about grandmothers being a barrier
to the intervention is not true of maternal grandmothers where the interaction effect indicated an enhancing effect, albeit non-significant. And, related to point 1, these statements imply some causality that can¹t be known for sure.

Thank you for raising this point. While collecting the qualitative data, we were, despite many efforts, unable to obtain much distinction between maternal and paternal grandmothers. As a result, we report qualitative findings from grandmothers as a whole. As highlighted at the start of the `Discussion' section, we collected the qualitative data to learn more about the potential mechanisms for the quantitative result. We noticed that the distinction between the qualitative and quantitative findings was not very clear in the Discussion section. We have amended this to make it clearer.

6. As a general comment following on from many of these points, the authors should consider using more circumspect language when interpreting results. The models only indicate associations and it is important not to assume too much about causal pathways. Relatedly, it is also important not to treat non-significant results as evidence, since they are rather a lack of evidence, i.e. we can never accept the null hypothesis.

We agree with the referee about the need to be careful about the language used in interpreting the findings, particularly those related to the interaction effects. Given that we have an RCT for the intervention, we believe that we can interpret the overall intervention effect in a causal manner. However, as mentioned, the interaction effects need to be interpreted more carefully, since the extended family variables are not randomly assigned. Indeed, we point out this limitation in the discussion (see p. 19). Regarding your point on not treating non-significant results as evidence, we have tried to be careful on this, but do let us know if we have overlooked it somewhere.

VERSION 3 – REVIEW

**REVIEWER**	Scott R Walter<br>Macquarie University
**REVIEW RETURNED**	05-Apr-2018

**GENERAL COMMENTS**	All my comments have been adequately addressed. I thank the authors for their patience with my persistent comments about the quantitative details of this study, and I hope they will agree the paper is all the stronger for it.
